# ﻿Systematic study of *Panaeolus* (*Agaricales*, *Galeropsidaceae*) sensu lato and psilocybin-producing traits of species from China

**DOI:** 10.3897/imafungus.17.167329

**Published:** 2026-01-19

**Authors:** Mao-Qiang He, Wen-Qiang Yang, Dorji Phurbu, Fei Liu, Jia-Xin Li, Bin Cao, Rui-Lin Zhao

**Affiliations:** 1 College of Resource Sciences and Technology, Sichuan Agricultural University, Chengdu 611130, China Chinese Academy of Sciences Beijing China; 2 State Key Laboratory of Microbial Diversity and Innovative Utilization, Institute of Microbiology, Chinese Academy of Sciences, Beijing 100101, China Sichuan Agricultural University Chengdu China; 3 Xizang Key Laboratory of Plateau Fungi, Institute of Plateau Biology of Xizang Autonomous Region, Lhasa 850000, China Institute of Plateau Biology of Xizang Autonomous Region Lhasa China; 4 College of Life Sciences, University of Chinese Academy of Sciences, Huairou District, Beijing, 100408, China University of Chinese Academy of Sciences Beijing China

**Keywords:** *
Agaricineae
*, *
Galeropsidaceae
*, hallucinogenic mushrooms, psychedelic fungi

## Abstract

*Panaeolus* sensu lato is a group of hallucinogenic mushrooms commonly found on dung, in pasture areas, grasslands, and forests. Previous studies indicated that the *Panaeolus* s.l. clade (panaeo-clade) could be ranked as a family (*Galeropsidaceae*), pending further evidence. In this study, based on phylogenomic, multigene phylogenetic, molecular clock, and morphological analyses, the panaeo-clade is demonstrated to be a distinct family, separate from *Bolbitiaceae*. The taxonomic system of *Galeropsidaceae* is revised. The genera accepted in *Galeropsidaceae* are *Panaeolus* and *Staktophyllus*, whereas *Crucispora* and *Panaeolopsis* are synonymized under *Panaeolus*. Three subgenera are accepted in *Panaeolus*: subg. Bresadolomyces, subg. Panaeolina, and subg. Panaeolus. Subgenus Bresadolomyces is roughly equivalent to the traditional circumscription of subg. Copelandia but is extended to include species formerly placed in *Crucispora*. Subgenus Panaeolina comprises most species from China and Anellaria-like species. Subgenus Panaeolus mainly comprises the *P. papilionaceus* species complex and a western Asian clade represented by *P. punjabensis*. In this study, one new subgenus and eight new species are proposed. Species from China are documented with descriptions, photographs, and illustrations. Additionally, the psilocybin-producing traits of 14 species were tested using high-performance liquid chromatography–tandem mass spectrometry (HPLC–MS). Two species are confirmed to possess psilocybin-producing traits, namely the known species *P. cinctulus* and the new species *P. subfoenisecii* proposed in this study. The evolution of the coprophilous lifestyle and psilocybin-producing traits in *Panaeolus* is also discussed based on phylogenetic relationships and divergence times.

## ﻿Introduction

*Panaeolus* sensu lato is a group of small brown mushrooms commonly found on dung, in pastures, grasslands, and forests. They are well known as magic mushrooms because of the psilocybin-producing traits of some species, the most well known being *P. cyanescens* (Berk. & Broome) Sacc. (Stijve 1992). Species of *Panaeolus* s.l. are characterized by a combination of the following features: small basidiomes (pileus typically up to 5 cm in diameter, though reaching 10 cm in *P. semiovatus* (Sowerby) S. Lundell & Nannf.; Stamets 1996), a black spore print, lamellae adnate, black or grayish black, mottled or spotted due to uneven maturation of the basidiospores, dark basidiospores with thick walls and a germ pore, and cystidia including cheilocystidia, pleurocystidia, caulocystidia, and occasionally pileocystidia and pseudocystidia. Some morphological characteristics of *Panaeolus* s.l. exhibit strong plasticity depending on habitat or basidiome developmental stage. For example, Thai samples of *P. antillarum* (Fr.) Dennis form a mottled and streaked pileus that contrasts markedly with specimens from other regions (Desjardin and Perry 2017). This morphological plasticity has resulted in the recognition of many varieties. According to Index Fungorum, there are 199 names under *Panaeolus*, of which 41 are varietal names. Taking the type species *P. papilionaceus* (Bull.) Quél. as an example, there are five variety names, namely P. papilionaceus var. capitatocystis E. Ludw., P. papilionaceus var. microsporus Speg., P. papilionaceus var. papilionaceus (Bull.) Quél., P. papilionaceus var. parvisporus Ew. Gerhardt, and P. papilionaceus var. retirugis (Fr.) Gminder, as well as three widely used synonym names, viz. *P. campanulatus* (L.) Quél., *P. retirugis* (Fr.) Gillet, and *P. sphinctrinus* (Fr.) Quél. Despite nearly 200 published names, the number of accepted species is considerably lower, with 20 species recognized by Ola’h (1969), 29 by Singer (1986), 32 by Gerhardt (1996), and 15 by He et al. (2019). Strauss et al. (2023) accepted 77 legitimate *Panaeolus* s.l. species worldwide.

Traditionally, four subgenera within *Panaeolus* s.l. have been proposed based on phenotypic features: subg. Anellaria P. Karst., characterized by relatively large basidiomes and a white, viscid pileus (Karsten 1879); subg. Copelandia Bres., comprising species primarily from tropical and subtropical regions whose basidiomes turn blue when bruised (Bresadola 1913); subg. Panaeolina Maire, grouping species with roughened spores (Maire 1933); and subg. Panaeolus, encompassing the remaining species. Under the concept of *Panaeolus* sensu stricto, Panaeolus s.s. corresponds to subg. Panaeolus, whereas the other three subgenera are often treated as distinct genera (Singer 1986). The familial placement of *Panaeolus* s.l. based on phenotypic features has long been controversial. Characters used to infer relationships include spore print color, types of pileipellis, hallucinogenic properties, and spore color changes in concentrated sulfuric acid. Based on these characters, *Panaeolus* s.l. has been classified within *Coprinaceae* (Singer 1986), *Psathyrellaceae* (Kirk et al. 2008), or *Strophariaceae* (Hawksworth et al. 1995).

Molecular data have helped clarify the familial relationships of *Panaeolus* s.l. Phylogenetic analyses based on LSU or ITS sequences consistently recover a clade, here termed the panaeo-clade, corresponding to *Panaeolus* s.l., showing a closer affinity to *Bolbitiaceae* than to *Coprinaceae*, *Psathyrellaceae*, or *Strophariaceae* (Hopple and Vilgalys 1999; Moncalvo et al. 2002; Walther et al. 2005; Malysheva et al. 2019). However, a study using six genes revealed a different placement for the panaeo-clade, grouping it with *Inocybeaceae*, *Crepidotaceae*, and *Tubariaceae*, distant from *Bolbitiaceae* (Matheny et al. 2006). Because of the unclear phylogenetic relationships within *Agaricales*, the panaeo-clade has been classified variously as the genus *Panaeolus* (Malysheva et al. 2019), the tribe *Panaeoleae* (Matheny et al. 2006), or the subfamily *Panaeoloideae* (Tóth et al. 2013). Under nomenclatural rules, the appropriate name for the panaeo-clade should not be derived from *Panaeolus*. The type of *Galeropsis* Velen. (*Galeropsidaceae*), *G. desertorum* Velen. & Dvořák, has been shown to be a member of the panaeo-clade (Malysheva et al. 2019). Consequently, the name *Galeropsidaceae*, previously applied only to secotioid taxa (Singer 1962), would be the valid name for this clade if it is recognized at the family rank within *Agaricales* (Kalichman et al. 2020).

Several *Panaeolus* s.l. species, such as *P. cyanescens* and *P. bisporus* (Malençon & Bertault) Ew. Gerhardt, are well known for their confirmed psilocybin-producing capabilities (Stijve 1992; Senn-Irlet et al. 1999). However, the occurrence of psilocybin in other species remains contentious. For example, samples of *P. antillarum* from Poland contained no psilocybin or psilocin (Halama et al. 2014), whereas samples from Taiwan Island did contain psilocybin and psilocin (Wang and Tzean 2015). Another example is *P. foenisecii* (Pers.) J. Schröt., for which the psychoactive properties have long been debated (Guzmán et al. 1998). Chemical analysis provides definitive evidence, suggesting that misidentification may be a primary reason for conflicting reports on the psilocybin-producing traits of *Panaeolus* s.l. species. Current estimates suggest that 10 to 20 species are psychoactive (He et al. 2022; Strauss et al. 2023).

In this study, we present a systematic investigation of *Panaeolus* s.l. based on 104 specimens collected from China, with the oldest dating to 1958. Using these Chinese specimens together with available data from major public sequence repositories (GenBank), this study aims to (1) clarify the phylogenetic position of the panaeo-clade within *Agaricineae*; (2) resolve the phylogenetic relationships among species of *Panaeolus* s.l.; (3) revise the current taxonomic system of *Panaeolus* s.l. based on phylogenetic analyses, morphological characteristics, and divergence times; (4) confirm the psilocybin-producing properties of each species using high-performance liquid chromatography–tandem mass spectrometry (HPLC–MS); and (5) document the species diversity of *Panaeolus* s.l. in China.

## ﻿Materials and methods

### ﻿Morphological examination

All newly collected specimens were photographed *in situ*. Macro-morphological characteristics and biochemical color reactions were recorded from fresh specimens. The specimens were dried in a food dryer at 50 °C. Anatomical and cytological features, including basidiospores, basidia, cystidia, and pileipellis, were observed under an Olympus CX31 microscope. At least 20 measurements were taken. Data were recorded as follows: X = mean of length × width ± SD; Q = quotient of basidiospore length to width; and Qm = mean of Q values ± SD. The protocol for morphological study and chemical reactions followed Largent’s methodology (Largent 1986). Specimens are deposited in the Herbarium Mycologicum Academiae Sinicae (HMAS).

### ﻿Phylogenomic and phylogenetic analyses

Information on reference genomes is presented in Table 1. Information on newly generated and reference sequences used in the multigene phylogenetic analyses is presented in Table 2. In the phylogenomic analysis, genome completeness was assessed using BUSCO v5.2.0 (Simão et al. 2015), employing default parameters and a set of 1,764 predefined orthologs from the basidiomycota_odb10 database. A data matrix was constructed from 1,764 single-copy, full-length BUSCO genes across the analyzed genomes. Each gene was aligned using MAFFT v7.490 (Katoh and Standley 2013), and ambiguous regions were trimmed using trimAl v1.4 (gappyout option). The resulting amino acid alignments, with more than 80% taxon occupancy per gene, were concatenated into a comprehensive data matrix. Phylogenetic analyses were conducted using IQ-TREE v2.0.3, with the best-fit amino acid substitution model selected automatically.

**Table 1.** Genomic information of the samples used in the phylogenomic analysis.

Family	Species	Strain/Accession	Reference
* Agaricaceae *	*Agaricus bisporus*	GCF_000300555.1	Morin et al. 2012
* Agaricaceae *	*Coprinus comatus*	GCA_003316025.1	Li et al. 2018
* Agaricaceae *	*Lepiota venenata*	GCA_004296355.1	Lüli et al. 2019
* Agaricaceae *	*Leucoagaricus* sp.	GCA_001563735.1	unpublished
* Agaricaceae *	*Macrolepiota dolichaula*	GCA_003315915.1	Li et al. 2018
* Agaricaceae *	*Podaxis carcinomalis*	GCA_018524395.1	Conlon et al. 2021
* Amanitaceae *	*Amanita muscaria*	Koide v1.0	Kohler et al. 2015
* Cortinariaceae *	*Cortinarius glaucopus*	GCA_015039465.1	Miyauchi et al. 2020
* Crassisporiaceae *	*Crassisporium funariophilum*	GCA_014925845.1	Steindorff et al. 2021
* Crepidotaceae *	*Crepidotus variabilis*	GCA_015657495.1	Ruiz-Dueñas et al. 2021
* Galeropsidaceae *	*Panaeolus cyanescens*	GCA_002938355.1	Awan et al. 2018
* Galeropsidaceae *	*Panaeolus papilionaceus*	GCA_015501605.1	Ruiz-Dueñas et al. 2021
* Hydnangiaceae *	*Laccaria amethystina*	LaAM-08-1 v2.0	Kohler et al. 2015
* Hydnangiaceae *	*Laccaria bicolor*	GCF_000143565.1	Martin et al. 2008
* Hydnangiaceae *	*Laccaria trichodermophora*	GCA_018417955.1	Ángeles-Argáiz et al. 2024
* Hymenogastraceae *	*Flammula alnicola*	GCA_015499995.1	Ruiz-Dueñas et al. 2021
* Hymenogastraceae *	*Galerina marginata*	GCA_000697645.1	Riley et al. 2014
* Hymenogastraceae *	*Gymnopilus dilepis*	GCA_002938385.1	Reynolds et al. 2018
* Hymenogastraceae *	*Gymnopilus junonius*	GCA_015501075.1	Ruiz-Dueñas et al. 2021
* Hymenogastraceae *	*Hebeloma cylindrosporum*	GCA_000827355.1	Kohler et al. 2015
* Hymenogastraceae *	*Psilocybe azurescens*	GCA_019721835.1	McKernan et al. 2021
* Hymenogastraceae *	Psilocybe cf. subviscida	GCA_013368295.1	Floudas et al. 2020
* Hymenogastraceae *	*Psilocybe cyanescens*	GCA_002938375.1	Yan et al. 2021
* Hymenogastraceae *	*Psilocybe galindoi*	GCA_019721455.1	McKernan et al. 2021
* Hymenogastraceae *	*Psilocybe serbica*	v1.0	Fricke et al. 2017
* Hymenogastraceae *	*Psilocybe tampanensis*	GCA_019904355.1	McKernan et al. 2021
* Inocybaceae *	*Inocybe terrigena*	GCA_003347685.1	Bahram et al. 2018
* Nidulariaceae *	*Crucibulum laeve*	GCA_004379715.1	Varga et al. 2019
* Nidulariaceae *	*Cyathus striatus*	GCA_015501535.1	Xie et al. 2024
* Pluteaceae *	*Pluteus cervinus*	NL-1719 v1.0	Varga et al. 2019
* Psathyrellaceae *	*Candolleomyces aberdarensis*	GCA_004126415.1	unpublished
* Psathyrellaceae *	*Coprinellus angulatus*	GCA_013368325.1	Floudas et al. 2020
* Psathyrellaceae *	*Coprinellus micaceus*	GCA_004369175.1	Varga et al. 2019
* Psathyrellaceae *	*Coprinopsis cinerea*	GCF_000182895.1	Stajich et al. 2010
* Psathyrellaceae *	*Coprinopsis marcescibilis*	GCA_004369085.1	Varga et al. 2019
* Psathyrellaceae *	*Coprinopsis* sp.	GCA_020736565.1	Mesny et al. 2021
* Psathyrellaceae *	*Coprinopsis strossmayeri*	GCA_900156845.1	Banks et al. 2017
* Squamanitaceae *	*Floccularia luteovirens*	GCA_009739215.1	Liu et al. 2021
* Strophariaceae *	*Agrocybe pediades*	GCA_013053245.1	Li et al. 2024
* Strophariaceae *	*Hypholoma fasciculare*	GCA_016801325.1	Al-Salihi et al. 2019
* Strophariaceae *	*Hypholoma sublateritium*	GCA_000827495.1	Kohler et al. 2015
* Strophariaceae *	*Pholiota adiposa*	GCA_009935795.1	He et al. 2025
* Strophariaceae *	*Pholiota conissans*	CIRM-BRFM 674 v1.0	Ruiz-Dueñas et al. 2021
* Strophariaceae *	*Pholiota microspora*	GCA_003314615.1	Li et al. 2018
* Strophariaceae *	*Pholiota molesta*	GCA_014925825.1	Steindorff et al. 2021
* Strophariaceae *	*Stropharia rugosoannulata*	GCA_003314255.1	Li et al. 2018
* Tubariaceae *	*Cyclocybe aegerita*	GCA_902728275.1	Chen et al. 2024
* Tubariaceae *	*Cyclocybe cylindracea*	GCA_013376435.1	Liang et al. 2020
* Tubariaceae *	*Tubaria furfuracea*	GCA_900069095.1	Dentinger et al. 2016

**Table 2.** Sequence information for the samples used in the phylogenetic analyses.

Family name	Species name	Specimen number	Region	ITS	LSU	*Tef*1	*rpb*1	*rpb*2	SSU	Reference
* Agaricaceae *	*Agaricus campestris*	AFTOL-ID 1492	–	DQ486682	DQ110871	–	DQ516068	–	DQ113914	Matheny et al. 2006
* Agaricaceae *	*Calvatia gigantea*	DSH 96-032	Germany, Mecklenburg	AJ617492	AF518603	–	–	–	AF026622	Krüger and Gargas 2008
* Agaricaceae *	*Chlorophyllum agaricoides*	AFTOL-ID 440	Greece	DQ200928	AY700187	–	DQ447889	–	AY657010	Matheny et al. 2006
* Agaricaceae *	*Clarkeinda trachodes*	xml2014104	China	LT716022	KY418837	–	–	KY418989	–	Zhao et al. 2017
* Agaricaceae *	*Coniolepiota spongodes*	png012	Thailand, Chiang Mai Province	HM488756	HM488774	HM488883	–	HM488796	–	Vellinga et al. 2011
* Agaricaceae *	*Coprinus comatus*	AFTOL-ID 626	–	AY854066	AY635772	–	AY857983	AY780934	AY665772	Matheny et al. 2006
* Agaricaceae *	*Eriocybe chionea*	ecv3616 (T)	Thailand, Chiang Mai Province	HM488753	HM488772	–	–	HM488801	–	Vellinga et al. 2011
* Agaricaceae *	*Heinemannomyces* sp.	ZRL185	Thailand	KT951346	KT951527	KT951657	–	–	–	Zhao et al. 2016
* Agaricaceae *	*Hymenagaricus* sp.	AFTOL-ID 1383	–	DQ490633	DQ457680	–	–	–	DQ089016	Matheny et al. 2006
* Agaricaceae *	*Lepiota cristata*	ZRL20151133	China	LT716026	KY418841	KY419048	KY418963	KY418992	KY418910	Zhao et al. 2017
* Agaricaceae *	*Leucocoprinus fragilissimus*	ZRL20151466	China	LT716029	KY418844	KY419049	KY418965	KY418994	KY418913	Zhao et al. 2017
* Agaricaceae *	*Lycoperdon ericaeum*	ZRL20151498	China	LT716030	KY418845	–	KY418966	KY418995	KY418914	Zhao et al. 2017
* Agaricaceae *	*Macrolepiota dolichaula*	xml2013058	China	LT716021	KY418836	KY419044	–	KY418988	–	Zhao et al. 2017
* Agaricaceae *	*Micropsalliota globocystis*	ZRL2013465	China	LT716024	KY418839	KY419046	–	KY418991	–	Zhao et al. 2017
* Agaricaceae *	*Verrucospora flavofusca*	AFTOL-ID 655	–	DQ241779	DQ470825	–	–	–	AY665783	Matheny et al. 2006
* Amanitaceae *	*Amanita brunnescens*	AFTOL-ID 673	–	AY789079	AY631902	AY881021	AY788847	AY780936	AY707096	Matheny et al. 2006
* Amanitaceae *	*Amanita muscaria*	HKAS61888	China, Heilongjiang Province	MH508439	MH486651	MH508908	–	MH486100	–	Cui et al. 2018
* Amanitaceae *	*Catatrama costaricensis*	DAOM 211663	Costa Rica	–	KT833804	KT833834	–	KT833819	–	Yang et al. 2018
* Amanitaceae *	*Limacella delicata*	ZT Myc 55818	Switzerland	–	KT833807	KT833835	–	KT833822	–	Yang et al. 2018
* Amanitaceae *	*Limacellopsis guttata*	MB-100157	Germany	–	KT833813	KT833841	–	KT833828	–	Yang et al. 2018
* Bolbitiaceae *	*Bolbitius subvolvatus*	WU28379	China	JX968248	JX968365	JX968454	–	–	–	Toth et al. 2013
* Bolbitiaceae *	*Bolbitius vitellinus*	AFTOL-ID 730	USA, Washington	DQ200920	AY691807	DQ408148	DQ435802	DQ385878	AY705955	Matheny et al. 2006
* Bolbitiaceae *	*Conobolbitina micheliana*	HMJAU65015 (T)	China	OR995677	OR994080	PP000869	–	–	–	Song and Bau 2024
* Bolbitiaceae *	*Conobolbitina pygmaeoaffinis*	WU16600	China	JX968149	–	JX968382	–	–	–	Toth et al. 2013
* Bolbitiaceae *	*Conocybe lactea*	AFTOL-ID 1675	USA, Massachusetts	DQ486693	DQ457660	–	DQ447893	DQ470834	DQ437683	Matheny et al. 2006
* Bolbitiaceae *	*Conocybe semiglobata*	WU8794	–	JX968188	JX968304	–	–	–	–	Toth et al. 2013
* Bolbitiaceae *	*Conocybe tenera*	SZMC-NL-1615	–	JX968180	JX968296	JX968404	–	–	–	Toth et al. 2013
* Bolbitiaceae *	*Conocybula coprophila*	HMJAU62008	China	OR995662	OR995712	PP000855	–	–	–	Song and Bau 2024
* Bolbitiaceae *	*Conocybula longistipitata*	HMJAU64974	China	OR995664	OR995714	PP000857	–	–	–	Song and Bau 2024
* Bolbitiaceae *	*Descolea antarctica*	NZ5182	–	AF325647	–	–	–	–	–	Peintner et al. 2001
* Bolbitiaceae *	*Descolea quercina*	HMJAU64959	China	OQ780313	OQ758213	OQ758299	–	–	–	Song and Bau 2023
* Bolbitiaceae *	*Galerella nigeriensis*	CNF1/5859	–	JX968251	JX968368	JX968457	–	–	–	Toth et al. 2013
* Bolbitiaceae *	*Panaeolus desertorum*	SZMC-NL-1863	–	JX968154	JX968271	JX968387	–	–	–	Toth et al. 2013
* Bolbitiaceae *	*Pholiotina aporos*	SZMC-NL-1241	–	JX968260	JX968376	JX968462	–	–	–	Toth et al. 2013
* Bolbitiaceae *	*Pholiotina changbaishanensis*	HMJAU65101	China	OR995689	OR994092	PP000881	–	–	–	Song and Bau 2024
* Bolbitiaceae *	*Pholiotina excrescenticystidiata*	HMJAU65021	China	OR995695	OR994098	PP000887	–	–	–	Song and Bau 2024
* Bolbitiaceae *	*Pholiotina intermedia*	HMJAU62014	China	OR995667	OR995717	PP000860	–	–	–	Song and Bau 2024
* Bolbitiaceae *	*Pholiotina serrata*	HMJAU62006	China	OP538570	OQ758217	OQ758301	–	–	–	Song and Bau 2024
* Cortinariaceae *	*Aureonarius kroegeri*	F15952 (T)	–	FJ157053	–	–	–	–	–	Harrower et al. 2011
* Cortinariaceae *	*Calonarius typicus*	H7068029 (T)	USA, Florida	NR173069	–	–	–	–	–	Liimatainen et al. 2022
* Cortinariaceae *	*Cortinarius violaceus*	Moser 74/208 (T)	Sweden	NR173726	–	–	–	–	–	Liimatainen et al. 2022
* Cortinariaceae *	*Cystinarius rubiginosus*	H7072000 (T)	USA, California	NR182475	–	–	–	–	–	Liimatainen et al. 2022
* Cortinariaceae *	*Hygronarius renidens*	Kytovuori 00-021 (T)	Finland, Varsinais–Suomi	NR175772	–	–	–	–	–	Liimatainen et al. 2022
* Cortinariaceae *	*Hygronarius renidens*	OS582	Norway	KC842459	KC842529	–	–	–	–	Stensrud et al. 2014
* Cortinariaceae *	*Mystinarius lustrabilis*	PC0088377 (T)	–	NR131792	–	–	–	–	–	Niskanen et al. 2006
* Cortinariaceae *	*Mystinarius lustrabilis*	TUB011835	–	AY669586	–	–	KJ403766	–	–	Garnica et al. 2005
* Cortinariaceae *	*Phlegmacium saginum*	T30	Norway	KC842448	KC842518	–	–	–	KC171290	Stensrud et al. 2014
* Cortinariaceae *	*Thaxterogaster magellanicus*	EN266	–	MN855079	–	–	–	–	–	Nouhra et al. 2021
* Cortinariaceae *	*Volvanarius chlorosplendidus*	K235086 (T)	Argentina, Bariloche	NR169962	–	–	–	–	–	Liimatainen and Niskanen 2020
* Crassisporiaceae *	*Crassisporium funariophilum*	IB1949/0008 (T)	Austria, Tyrol	NR172227	NG070812	–	–	–	–	Matheny et al. 2015
* Crassisporiaceae *	*Romagnesiella clavus*	PAM06090110 (T)	France	NR171207	NG070809	–	–	–	–	Matheny et al. 2007
* Crepidotaceae *	Crepidotus cf. applanatus	PBM 717	USA, Washington	DQ202273	AY380406	–	AY333303	AY333311	AY705951	Matheny et al. 2006
* Crepidotaceae *	*Crepidotus mollis*	TUB 011566	–	–	DQ071698	–	KF211308	–	–	Garnica et al. 2007
* Crepidotaceae *	*Neopaxillus dominicanus*	MCVE 26928	Dominican Republic	JN033216	JN033217	–	–	–	–	Vizzini et al. 2012
* Crepidotaceae *	*Neopaxillus plumbeus*	F 1068564 (T)	–	NR132860	NG060271	–	–	–	–	Vizzini et al. 2012
* Crepidotaceae *	*Pellidiscus pallidus*	C58178	Ecuador	AY571054	AY571017	–	–	–	–	Bodensteiner et al. 2004
* Crepidotaceae *	*Simocybe serrulata*	AFTOL-ID 970	–	DQ494696	AY745706	–	DQ447940	DQ484053	DQ465343	Matheny et al. 2006
* Galeropsidaceae *	*Panaeolopsis nirimbii*	G1701	Australia	–	MK278427	–	–	–	–	Varga et al. 2019
* Galeropsidaceae *	*Panaeolopsis* sp.	Mushroom Observer 161213	Canada, Saskatchewan	MW183929	–	–	–	–	–	unpublished
* Galeropsidaceae *	*Panaeolus acuminatus*	CBS:270.47	–	MH856251	MH867783	–	–	–	–	Vu et al. 2019
* Galeropsidaceae *	*Panaeolus acuminatus*	CBS:269.47	–	MH856250	MH867782	–	–	–	–	Vu et al. 2019
* Galeropsidaceae *	*Panaeolus acuminatus*	GLM 46071	–	–	DQ071695	–	DQ067964	–	–	Garnica et al. 2007
* Galeropsidaceae *	*Panaeolus acuminatus*	TFB8626	Argentina, Puerto Chucao	KY559329	–	–	–	MF978334	–	unpublished
* Galeropsidaceae *	*Panaeolus alcis*	Mushroom Observer 88085	Sweden	KM982723	–	–	–	–	–	unpublished
* Galeropsidaceae *	*Panaeolus alcis*	SAT-14-239-20	USA	MW597122	–	–	–	–	–	unpublished
* Galeropsidaceae *	*Panaeolus antillarum*	ZRL20191951	–	PP475257	PP472834	PP554377	PP556837	PP852766	PP472859	this study
* Galeropsidaceae *	*Panaeolus antillarum*	HMAS37291	China, Bejing	PP475256	PP472831	–	–	–	PP472835	this study
* Galeropsidaceae *	*Panaeolus antillarum*	HMAS69911	China, Hebei Province	PP475260	PP472753	PP554379	–	–	–	this study
* Galeropsidaceae *	*Panaeolus antillarum*	HMAS52750	China, Xizang Autonomous Region	PP475258	PP472833	PP554378	–	PP852765	–	this study
* Galeropsidaceae *	*Panaeolus antillarum*	HMAS52751	China, Xizang Autonomous Region	PP475259	PP472832	–	–	–	–	this study
* Galeropsidaceae *	*Panaeolus axfordii*	MFLU 19-2367	China, Yunnan Province	NR_169700	–	–	–	–	–	Hu et al. 2020
* Galeropsidaceae *	*Panaeolus bisporus*	KaiR95	Benin	MT110229	–	–	–	–	–	Piepenbring et al. 2020
* Galeropsidaceae *	*Panaeolus bisporus*	MushroomObserver 188954	USA, Ohio	MG966283	–	–	–	–	–	unpublished
* Galeropsidaceae *	Staktophyllus cf. guttulatus	G0217	Hungary	–	MK278432	–	–	–	–	Varga et al. 2019
* Galeropsidaceae *	*Panaeolus cinctulus*	ZRL20191912	China, Inner Mongolia Autonomous Region	–	PP472799	PP556781	PP832222	PP852767	PP472838	this study
* Galeropsidaceae *	*Panaeolus cinctulus*	HMAS63178	China, Ningxia Hui Autonomous Region	PP475250	PP472800	PP556783	–	–	–	this study
* Galeropsidaceae *	*Panaeolus cinctulus*	CBS:331.34	–	MH855554	MH867059	–	–	–	–	Vu et al. 2019
* Galeropsidaceae *	*Panaeolus cinctulus*	NX180911-04	China, Ningxia Hui Autonomous Region	MN960188	–	–	–	–	–	unpublished
* Galeropsidaceae *	*Panaeolus cinctulus*	ZRL20200005	China, Beijing	–	PP472801	PP556782	PP832223	PP852768	PP472839	this study
* Galeropsidaceae *	*Panaeolus cyanescens*	HMAS57723	China,Guizhou Province	PP475202	PP472752	PP556776	–	–	–	this study
* Galeropsidaceae *	*Panaeolus cyanescens*	NBRC-30222	Japan	AB158633	–	–	–	–	–	Maruyama et al. 2006
* Galeropsidaceae *	*Panaeolus cyanescens*	MW-2010	–	HM035085	HM035085	–	–	–	–	unpublished
* Galeropsidaceae *	Panaeolus cyanescens var. bisporus	n. 6576 AQUI	Italy	EU834287	EU834287	–	–	–	–	unpublished
* Galeropsidaceae *	*Panaeolus desertorum*	AH 9993 (paratype)	Spain	MK397543	MK397561	–	–	–	–	Malysheva et al. 2019
* Galeropsidaceae *	*Panaeolus desertorum*	SZMC-NL-1863	–	JX968154	JX968271	JX968387	–	–	–	Toth et al. 2013
* Galeropsidaceae *	*Panaeolus detriticola*	PERTH 08944954 (T)	Australia	NR_199086	–	–	–	–	–	unpublished
* Galeropsidaceae *	*Panaeolus fimicola*	4080	Italy	JF908518	–	–	–	–	–	Osmundson et al. 2013
* Galeropsidaceae *	*Panaeolus fimicola*	CBS:251.37	–	MH855904	MH867411	–	–	–	–	Vu et al. 2019
* Galeropsidaceae *	*Panaeolus fimicola*	iNat72986889	USA, Lane County	OQ383438	–	–	–	–	–	unpublished
* Galeropsidaceae *	*Panaeolus fimicola*	4350	Italy	JF908519	–	–	–	–	–	Osmundson et al. 2013
* Galeropsidaceae *	*Panaeolus foenisecii*	ZRL20210662	–	PP475255	PP472795	PP556786	PP832226	–	PP472861	this study
* Galeropsidaceae *	*Panaeolus foenisecii*	FO 46609	–	–	DQ071696	–	DQ067963	–	–	Garnica et al. 2007
* Galeropsidaceae *	*Panaeolus foenisecii*	CBS:142.40	–	MH856067	MH867557	–	–	–	–	Vu et al. 2019
* Galeropsidaceae *	*Panaeolus foenisecii*	K(M):250281	United Kingdom, Buckinghamshire	MZ159698	–	–	–	–	–	unpublished
* Galeropsidaceae *	*Panaeolus foenisecii*	J152	–	–	AF041537	–	–	–	DQ851578	Hopple and Vilgalys 1999
* Galeropsidaceae *	*Panaeolus foenisecii*	ZRL20210661	China, Xizang Autonomous Region	PP475254	–	–	–	–	–	this study
* Galeropsidaceae *	*Panaeolus foenisecii*	ZRL20220802	China, Xizang Autonomous Region	PP475253	PP472796	PP556787	PP832227	PP852770	PP472862	this study
* Galeropsidaceae *	*Panaeolus fraxinophilus*	MushroomObserver 455364	USA, Kentucky	OL629088	–	–	–	–	–	unpublished
* Galeropsidaceae *	*Panaeolus grandis*	ZRL20220352	–	PP475283	PP472808	PP556823	PP850999	PP852786	PP472878	this study
* Galeropsidaceae *	*Panaeolus grandis*	ZRL20220208 (T)	China, Xizang Autonomous Region	PP475284	PP472809	PP556824	PP850998	PP852787	PP472879	this study
* Galeropsidaceae *	*Staktophyllus guttulatus*	137	Iran	MH592651	–	–	–	–	–	unpublished
* Galeropsidaceae *	Staktophyllus guttulatus var. guttulatus	STA5	Iraq	LC458688	–	–	–	–	–	unpublished
* Galeropsidaceae *	*Panaeolus limoniformisporus*	ZRL20220678	China, Xizang Autonomous Region	PP475289	PP472813	PP556818	PP850987	PP852774	PP472885	this study
* Galeropsidaceae *	*Panaeolus limoniformisporus*	ZRL20180975	China, Gansu Province	PP475285	PP472816	PP556820	PP850989	PP852773	PP472850	this study
* Galeropsidaceae *	*Panaeolus limoniformisporus*	ZRL20181122 (T)	China, Gansu Province	PP475290	PP472814	PP556822	PP850988	PP852776	PP472849	this study
* Galeropsidaceae *	*Panaeolus limoniformisporus*	ZRL20200165	China, Sichuan Province	PP475286	PP472815	PP556817	PP850990	–	PP472841	this study
* Galeropsidaceae *	*Panaeolus limoniformisporus*	ZRL2015390	China, Sichuan Province	PP475287	PP472818	PP556821	PP850991	PP852777	PP472884	this study
* Galeropsidaceae *	*Panaeolus limoniformisporus*	ZRL20152331	China, Xizang Autonomous Region	PP475288	PP472817	PP556819	PP850986	PP852775	PP472898	this study
* Galeropsidaceae *	*Panaeolus limoniformisporus*	ZRL20220678	China, Xizang Autonomous Region	PP475289	PP472813	PP556818	PP850987	PP852774	PP472885	this study
* Galeropsidaceae *	*Panaeolus medogensis*	ZRL20210733 (T)	China, Xizang Autonomous Region	PP475291	PP472812	–	PP850985	–	PP472840	this study
* Galeropsidaceae *	*Panaeolus mexicanus*	ANGE1557	Dominican Republic	MZ856314	–	–	–	–	–	Voto and Angelina 2021
* Galeropsidaceae *	*Panaeolus nigrescens*	ZRL20181924	China, Gansu Province	PP475293	PP472754	PP556836	PP850992	PP852779	PP472846	this study
* Galeropsidaceae *	*Panaeolus nigrescens*	ZRL20180732 (T)	China, Gansu Province	PP475292	PP472757	–	–	PP852778	PP472848	this study
* Galeropsidaceae *	*Panaeolus nigrescens*	ZRL20161807	China, Gansu Province	PP475295	PP472756	PP556833	–	PP852780	PP472847	this study
* Galeropsidaceae *	*Panaeolus nigrescens*	ZRL20161828	China, Gansu Province	PP475296	PP472755	PP556835	–	PP852781	PP472886	this study
* Galeropsidaceae *	*Panaeolus nigrescens*	ZRL20161875	China, Gansu Province	PP475294	PP472758	PP556834	–	–	PP472887	this study
* Galeropsidaceae *	*Panaeolus nigrescens*	ZRL20181924	China, Gansu Province	PP475293	PP472754	PP556836	PP850992	PP852779	PP472846	this study
* Galeropsidaceae *	*Panaeolus pallidus*	ZRL20180988 (T)	China, Gansu Province	PP475282	PP472811	PP556826	PP850984	PP852772	PP472851	this study
* Galeropsidaceae *	*Panaeolus pallidus*	ZRL20190137	China, Beijing	PP475281	PP472810	PP556825	–	PP852771	PP472877	this study
* Galeropsidaceae *	*Panaeolus paludosus*	B2082	Australia	–	MK278434	–	–	–	–	Varga et al. 2019
* Galeropsidaceae *	*Panaeolus pantropicalis*	JBSD 130972 (T)	Dominican Republic	PP590036	–	–	–	–	–	Voto and Angelini 2024
* Galeropsidaceae *	*Panaeolus pantropicalis*	MHHNU 31396	China	OP862800	–	–	–	–	–	unpublished
* Galeropsidaceae *	*Panaeolus pantropicalis*	DNA1940	USA, Florida	KF830093.1	KF830082.1	–	–	KF830065.1	KF830073.1	unpublished
* Galeropsidaceae *	*Panaeolus papilionaceus*	ZRL20210652	China, Xizang Autonomous Region	PP475247	PP472773	PP556811	PP850967	PP852754	PP472893	this study
* Galeropsidaceae *	*Panaeolus papilionaceus*	AFTOL-ID 1499	USA, Washington	DQ182503	DQ470817	–	–	–	DQ459375	Matheny et al. 2006
* Galeropsidaceae *	*Panaeolus papilionaceus*	ZRL20220203	China, Xizang Autonomous Region	PP475238	PP472775	–	–	–	PP472889	this study
* Galeropsidaceae *	*Panaeolus papilionaceus*	ZRL20220153	China, Xizang Autonomous Region	PP475239	PP472787	PP556807	PP850972	–	PP472870	this study
* Galeropsidaceae *	*Panaeolus parvisporus*	ZRL20170602	China, Inner Mongolia Autonomous Region	PP475214	PP472786	PP556793	PP850963	PP852758	PP472864	this study
* Galeropsidaceae *	*Panaeolus parvisporus*	CBS 276.39	–	MH856012	–	–	–	–	–	Vu et al. 2019
* Galeropsidaceae *	*Panaeolus parvisporus*	7070	Italy	JF908521	–	–	–	–	–	Osmundson et al. 2013
* Galeropsidaceae *	*Panaeolus parvisporus*	ZRL20170602	China, Inner Mongolia Autonomous Region	PP475214	PP472786	PP556793	PP850963	PP852758	PP472864	this study
* Galeropsidaceae *	*Panaeolus parvisporus*	ZRL20170603	China, Inner Mongolia Autonomous Region	PP475216	PP472772	PP556794	PP850964	PP852760	PP472865	this study
* Galeropsidaceae *	*Panaeolus parvisporus*	ZRL20170604	China, Inner Mongolia Autonomous Region	PP475212	PP472771	–	–	PP852759	PP472866	this study
* Galeropsidaceae *	*Panaeolus parvisporus*	ZRL20170654	China, Inner Mongolia Autonomous Region	PP475215	PP472784	PP556792	–	–	PP472867	this study
* Galeropsidaceae *	*Panaeolus parvisporus*	HMAS69762	China, Ningxia Hui Autonomous Region	PP475213	PP472783	PP556795	PP850965	–	–	this study
* Galeropsidaceae *	*Panaeolus plantaginiformis*	LE 2862 (lectotype)	Russia	MK397577	MK397599	–	–	–	–	Malysheva et al. 2019
* Galeropsidaceae *	*Panaeolus plantaginiformis*	LE 2863 (holotype)	Uzbekistan	MK397580	MK397602	–	–	–	–	Malysheva et al. 2019
* Galeropsidaceae *	*Panaeolus punjabensis*	LAH36794	Pakistan	–	ON116492	–	–	–	–	Asif et al. 2023
* Galeropsidaceae *	*Panaeolus punjabensis*	LAH36792	Pakistan	–	ON116491	–	–	–	–	Asif et al. 2023
* Galeropsidaceae *	*Panaeolus punjabensis*	LAH36793	Pakistan	MZ823627	ON116490	–	–	–	–	Asif et al. 2023
* Galeropsidaceae *	*Panaeolus punjabensis*	LAH37417	Pakistan	OP681142	–	–	–	–	–	Asif et al. 2023
* Galeropsidaceae *	*Panaeolus punjabensis*	INNASA1	Iraq	MK500858	–	–	–	–	–	unpublished
* Galeropsidaceae *	*Panaeolus ranwuensis*	ZRL20210707 (T)	China, Xizang Autonomous Region	PP475220	PP472785	PP556788	PP850966	PP852735	PP472894	this study
* Galeropsidaceae *	*Panaeolus ranwuensis*	HMAS69910	China, Inner Mongolia Autonomous Region	PP475221	PP472781	PP556789	–	PP852734	–	this study
* Galeropsidaceae *	*Panaeolus rhombisperma*	CWN 11502	Taiwan Island	MZ782082	MZ781504	–	–	–	–	Chou et al. 2023
* Galeropsidaceae *	*Panaeolus semiovatus*	ZRL20181933	–	PP475266	PP472822	PP554381	PP556842	PP852763	PP472857	this study
* Galeropsidaceae *	*Panaeolus semiovatus*	ZRL20201190	China, Sichuan Province	PP475272	PP472829	PP554388	PP556843	PP852764	PP472856	this study
* Galeropsidaceae *	*Panaeolus semiovatus*	ZRL20201261	China, Sichuan Province	PP475261	PP472821	PP554380	–	–	PP472855	this study
* Galeropsidaceae *	*Panaeolus semiovatus*	ZRL20201278	China, Sichuan Province	PP475264	PP472826	PP554382	PP556844	–	PP472895	this study
* Galeropsidaceae *	*Panaeolus semiovatus*	ZRL20210938	China, Xizang Autonomous Region	PP475273	PP472823	PP554385	PP556841	PP852761	PP472860	this study
* Galeropsidaceae *	*Panaeolus semiovatus*	ZRL20210939	China, Xizang Autonomous Region	PP475274	PP472820	PP554386	PP556845	–	PP472888	this study
* Galeropsidaceae *	*Panaeolus semiovatus*	ZRL20220286	China, Xizang Autonomous Region	PP475265	PP472824	PP554387	PP556846	–	PP472900	this study
* Galeropsidaceae *	*Panaeolus* sp.	HMAS72941	China, Guangxi Province	PP475209	PP472751	PP556791	–	–	–	this study
* Galeropsidaceae *	*Panaeolus* sp.	HMAS69946	China, Ningxia Hui Autonomous Region	PP475207	PP472792	PP556778	–	–	–	this study
* Galeropsidaceae *	*Panaeolus* sp.	PBM4141	–	MG773818	–	–	–	–	–	unpublished
* Galeropsidaceae *	*Panaeolus* sp.	HMAS69980	China, Ningxia Hui Autonomous Region	PP475204	PP472791	PP556777	PP832221	PP852733	–	this study
* Galeropsidaceae *	*Panaeolus* sp.	X540	Czech Republic	MW352021	MW352021	–	–	–	–	Gotvaldová et al. 2022
* Galeropsidaceae *	*Panaeolus* sp.	iNAT:99905220	USA, New York	OL584501	–	–	–	–	–	unpublished
* Galeropsidaceae *	*Panaeolus* sp.	204	USA, Arizona	MK627501	–	–	–	–	–	Owen et al. 2019
* Galeropsidaceae *	*Panaeolus* sp.	NY04449017	Colombia	PP590035	–	–	–	–	–	Voto and Angelini 2024
* Galeropsidaceae *	*Panaeolus* sp.	CZ519-3	China	FJ755227	FJ755227	–	–	–	–	unpublished
* Galeropsidaceae *	*Panaeolus* sp.	HMAS57752	China, Guizhou Province	PP475210	–	PP556790	–	–	–	this study
* Galeropsidaceae *	*Panaeolus* sp.	HMAS72941	China, Guangxi Province	PP475209	PP472751	PP556791	–	–	–	this study
* Galeropsidaceae *	*Panaeolus* sp.	N.L. Bougher NLB 1553	Australia, Perth	MT571659	–	–	–	–	–	unpublished
* Galeropsidaceae *	*Panaeolus* sp.	MHHNU31392	China, Hunan Province	MK439503	–	–	–	–	–	unpublished
* Galeropsidaceae *	*Panaeolus* sp.	RA400	Iraq	MH632116	–	–	–	–	–	unpublished
* Galeropsidaceae *	*Panaeolus* sp.	HMAS63187	China, Ningxia Hui Autonomous Region	PP475203	–	PP556779	–	–	–	this study
* Galeropsidaceae *	*Panaeolus* sp.	HMAS69980	China, Ningxia Hui Autonomous Region	PP475204	PP472791	PP556777	PP832221	PP852733	–	this study
* Galeropsidaceae *	*Panaeolus* sp.	HMAS69846	China, Ningxia Hui Autonomous Region	PP475206	PP472794	–	–	–	–	this study
* Galeropsidaceae *	*Panaeolus* sp.	HMAS69959	China, Ningxia Hui Autonomous Region	PP475205	PP472793	PP556780	–	PP852732	–	this study
* Galeropsidaceae *	*Panaeolus subfoenisecii*	ZRL20220801	China, Xizang Autonomous Region	PP475251	PP472797	PP556784	PP832224	PP852769	PP472863	this study
* Galeropsidaceae *	*Panaeolus subfoenisecii*	ZRL20220850 (T)	China, Xizang Autonomous Region	PP475252	PP472798	PP556785	PP832225	–	PP472854	this study
* Galeropsidaceae *	*Panaeolus sylvaticus*	ANGE1393	Dominican Republic	OQ311002	–	–	–	–	–	Angelini and Voto 2019
* Galeropsidaceae *	*Panaeolus tropicalis*	taxon:1104351	China	JF961377	–	–	–	–	–	unpublished
* Galeropsidaceae *	*Panaeolus uliginosus*	DAOM 176594	Canada	AY129363	AY129384	–	–	–	–	Nugent and Saville 2004
* Galeropsidaceae *	*Panaeolus variabilicolor*	ZRL20220735	China, Xizang Autonomous Region	PP475279	PP472805	PP556829	PP850993	PP852784	PP472892	this study
* Galeropsidaceae *	*Panaeolus variabilicolor*	ZRL20210525	China, Xizang Autonomous Region	PP475275	PP472804	PP556827	PP850995	–	PP472897	this study
* Galeropsidaceae *	*Panaeolus variabilicolor*	ZRL20220144	China, Xizang Autonomous Region	PP475276	PP472803	PP556830	PP850996	PP852783	PP472880	this study
* Galeropsidaceae *	*Panaeolus variabilicolor*	ZRL20220096 (T)	China, Xizang Autonomous Region	PP475280	PP472802	PP556831	PP850997	PP852782	PP472881	this study
* Galeropsidaceae *	*Panaeolus variabilicolor*	ZRL20220075	China, Xizang Autonomous Region	PP475278	PP472807	PP556828	PP850994	PP852785	PP472882	this study
* Galeropsidaceae *	*Panaeolus variabilicolor*	ZRL20220205	China, Xizang Autonomous Region	PP475277	PP472806	PP556832	–	–	PP472883	this study
* Galeropsidaceae *	*Panaeolus xiaolanii*	ZRL20220560	China, Xizang Autonomous Region	PP475225	PP472760	PP556799	PP850976	PP852741	PP472891	this study
* Galeropsidaceae *	*Panaeolus xiaolanii*	ZRL20220031 (T)	China, Xizang Autonomous Region	PP475242	PP472769	PP556803	PP850978	PP852742	PP472902	this study
* Galeropsidaceae *	*Panaeolus xiaolanii*	ZRL20220039	China, Xizang Autonomous Region	PP475232	PP472763	PP556800	PP850979	PP852736	PP472852	this study
* Galeropsidaceae *	*Panaeolus xiaolanii*	ZRL20220044	China, Xizang Autonomous Region	PP475244	PP472759	PP556801	PP850980	PP852744	PP472873	this study
* Galeropsidaceae *	*Panaeolus nirimbii*	PERTH7680368	Australia	–	MK278427	–	–	–	–	Varga et al. 2019
* Galeropsidaceae *	*Staktophyllus guttulatus*	137	Iran	MH592651	–	–	–	–	–	unpublished
* Galeropsidaceae *	Staktophyllus guttulatus var. guttulatus	STA5	Iraq	LC458688	–	–	–	–	–	unpublished
* Galeropsidaceae *	Staktophyllus guttulatus var. guttulatus	AMB n. 18101	–	KU725993	–	–	–	–	–	unpublished
* Galeropsidaceae *	Staktophyllus guttulatus var. merrisiani	AMB n. 18102	–	KU725994	–	–	–	–	–	unpublished
* Galeropsidaceae *	*Staktophyllus* sp.	PBM4141	USA, Tennessee	MG773818	MT237467	–	–	–	–	unpublished
* Hydnangiaceae *	*Hydnangium carneum*	Trappe31123	Australia, Capital	KU685741	KU685892	KU686144	–	KU686038	–	Wilson et al. 2017
* Hydnangiaceae *	*Laccaria torosa*	SFC20150902-17 (T)	Korea	MG519561	MG519598	MG551664	–	MG551631	–	Cho et al. 2018
* Hydnangiaceae *	*Podohydnangium australe*	TM1026	Australia	KY073249	–	–	–	–	–	Sheedy et al. 2016
* Hymenogastraceae *	*Anamika indica*	IB19971307 (T)	India	AF407163	AF407164	–	–	–	–	Thomas et al. 2002
* Hymenogastraceae *	*Galerina vittiformis*	CBS:161.46	France	–	MH867673	–	–	–	–	Vu et al. 2019
* Hymenogastraceae *	Hebeloma cf. cavipes	ZRL20151612	China	LT716034	KY418849	KY419053	–	KY418997	–	Zhao et al. 2017
* Hymenogastraceae *	*Hebeloma fastibile*	IB19940036	–	AF325643	AY033139	AF388877	–	–	–	Peintner et al. 2001
* Hymenogastraceae *	*Naucoria escharioides*	PBM 1719	USA, Washington	AJ585430	AY380405	–	AY351840	AY337411		Matheny 2005
* Hymenogastraceae *	*Phaeocollybia lugubris*	14619	Italy	JF908574	–	–	–	–	–	Osmundson et al. 2013
* Hymenogastraceae *	*Psathyloma leucocarpum*	PDD 105593 (T)	–	–	NG059606	–	–	–	–	Soop et al. 2016
* Hymenogastraceae *	*Psathyloma leucocarpum*	PBM3116	New Zealand, North Island	HQ840659	HQ840660	–	–	HQ840662	HQ840661	Matheny et al. 2015
* Hymenogastraceae *	*Psilocybe semilanceata*	CBS 101868	United Kingdom	MH862763	–	–	–	–	–	Vu et al. 2019
* Hymenogastraceae *	*Psathyloma catervatim*	PBM3420	–	HQ840663	HQ840664	–	–	HQ840666	HQ840665	Matheny et al. 2015
* Inocybaceae *	*Auritella aureoplumosa*	PBM 2212	Western Australia	–	AY635765	–	–	AY635781	–	Matheny and Bougher 2006
* Inocybaceae *	*Auritella dolichocystis*	Trappe 24838 (T)	Australia, New South Wales	–	NG075155	–	–	–	–	Matheny and Bougher 2006
* Inocybaceae *	*Inocybe jarrahae*	PBM 2207	Western Australia	–	AY380381	–	AY351806	AY337382	–	Matheny 2005
* Inocybaceae *	*Inocybe relicina*	JV 10258	Finland	–	AY038324	–	AF389546	AY333778	–	Matheny et al. 2002
* Inocybaceae *	*Inosperma calamistratum*	PBM1105	USA, Washington	JQ801386	JQ815409	MK426203	MK415438	JQ846466	MK429958	Kropp et al. 2013
* Inocybaceae *	*Mallocybe terrigena*	JV16431	Sweden	AM882864	AY380401	–	AY333301	AY333309	–	Ryberg et al. 2008
* Inocybaceae *	*Nothocybe distincta*	ZT9250	India	KX171343	EU604546	MK426212	MK415444	EU600904	MK429965	Matheny et al. 2009
* Inocybaceae *	*Pseudosperma sororium*	PBM3901	USA, North Carolina	JQ408772	MH220278	MK426218	MK415447	MH249810	MK429971	Matheny et al. 2019
* Inocybaceae *	*Tubariomyces inexpectatus*	AH20390 (T)	Spain	GU907095	EU569855	–	–	GU907088	MK429973	Alvarado et al. 2010
* Mythicomycetaceae *	*Mythicomyces corneipes*	AFTOL-ID 972	–	DQ404393	AY745707	DQ029197	DQ447929	DQ408110	DQ092917	Matheny et al. 2006
* Mythicomycetaceae *	*Stagnicola perplexa*	AH 25260 (T)	Spain	MK351609	MK353793	–	–	MK359091	–	Vizzini et al. 2019
* Nidulariaceae *	*Crucibulum parvulum*	FLAS-F-66522	USA, Missouri	MT444036	MW600344	MW763092	–	MW646476	–	Kraisitudomsook et al. 2021
* Nidulariaceae *	*Cyathus stercoreus*	FLAS-F-66543	USA, California	MT444060	MW766997	MW763088	–	MW646480	–	Kraisitudomsook et al. 2021
* Nidulariaceae *	*Mycocalia denudata*	CBS-494.85	Canada	MT444107	MW600347	MW763084	–	MW646481	–	Kraisitudomsook et al. 2021
* Nidulariaceae *	*Nidula emodensis*	MES-3354	Chile	MT444079	MW600348	MW763093	–	–	–	Kraisitudomsook et al. 2021
* Nidulariaceae *	*Nidularia pulvinata*	FLAS-F-66545	USA, Ohio	MT444097	MW600354	MW763090	–	MW646486	–	Kraisitudomsook et al. 2021
* Pluteaceae *	*Pluteus romellii*	AFTOL-ID 625	–	AY854065	AY634279	AY883433	AY862187	AY786063	AY657014	Matheny et al. 2006
* Psathyrellaceae *	*Coprinellus curtus*	SZMC-NL-2339	–	FM878016	FM876273	FM897246	–	–	–	Nagy et al. 2010
* Psathyrellaceae *	*Coprinopsis atramentaria*	PBM992	USA, Washington	DQ486694	DQ457661	–	–	–	DQ115781	Matheny et al. 2006
* Psathyrellaceae *	*Cystoagaricus strobilomyces*	30-V-1997	Japan	AY176347	AY176348	–	–	–	–	Vellinga 2004
* Psathyrellaceae *	*Lacrymaria lacrymabunda*	AFTOL ID-478	–	DQ490639	AY700198	–	–	DQ472733	AY654885	Matheny et al. 2006
* Psathyrellaceae *	*Parasola conopilus*	ZRL20151990	China	LT716064	KY418880	–	–	KY419025	KY418946	Zhao et al. 2017
* Psathyrellaceae *	*Psathyrella candolleana*	ZRL20151400	China	LT716063	KY418879	KY419075	KY418978	KY419024	KY418945	Zhao et al. 2017
* Strophariaceae *	*Agrocybe praecox*	AFTOL ID-728	–	AY818348	AY646101	–	DQ516069	DQ385876	AY705956	Yang et al. 2005
* Strophariaceae *	*Bogbodia uda*	G0790	USA	–	MK278210	–	–	–	–	Varga et al. 2019
* Strophariaceae *	*Deconica* sp.	PBM3781	Australia	–	KF830081	–	KC669380	KF830064	KF830076	Ramírez-Cruz et al. 2013
* Strophariaceae *	*Hypholoma sublateritium*	AFTOL-ID 597	–	AY818349	AY635774	–	–	–	AY787215	Matheny et al. 2006
* Strophariaceae *	*Kuehneromyces rostratus*	AFTOL-ID 1676	–	DQ490638	DQ457684	GU187712	DQ447918	DQ472730	DQ457624	Matheny et al. 2006
* Strophariaceae *	*Leratiomyces tesquorum*	SAV F-4052 (T)	USA	MH043618	MH036177	–	–	–	–	Crous et al. 2018
* Strophariaceae *	*Melanotus hartii*	CBS:273.81 (T)	Canada, Ontario	MH861342	MH873101	–	–	–	–	Vu et al. 2019
* Strophariaceae *	*Pholiota squarrosa*	HMJAU37515	China	MN209777	MN251160	–	–	MN329733	–	Tian and Matheny 2021
* Strophariaceae *	*Protostropharia dorsipora*	Mushroom Observer 488159		OP297820	–	–	–	–	–	unpublished
* Strophariaceae *	*Pyrrhulomyces astragalinus*	PBM4330	USA, North Carolina	MT187979	MT228845	–	–	–	–	Tian and Matheny 2021
* Strophariaceae *	*Stropharia ambigua*	AFTOL-ID 726	–	AY818350	AY646102	GU187756	DQ447941	DQ484054	DQ092924	Matheny et al. 2006
* Tubariaceae *	*Flammulaster* sp.	PBM3449	Australia, Tasmania	HQ827176	HQ827177	–	–	–	HQ827178	Matheny et al. 2015
* Tubariaceae *	*Hemistropharia albocrenulata*	G0088	USA	–	MK278139	–	–	–	–	Varga et al. 2019
* Tubariaceae *	*Pachylepyrium fulvidula*	MICH 11636 (T)	Argentina, Tucuman	NR170724	NG073595	–	–	–	–	unpublished
* Tubariaceae *	*Pachylepyrium fulvidula*	T1495	Argentina, Tucuman	KF830091	KF830080	–	–	KF830063	KF830072	unpublished
* Tubariaceae *	*Phaeomarasmius proximans*	AFTOL-ID 979	–	DQ404381	–	DQ028592	–	AY333314	AY752970	Matheny et al. 2006
* Tubariaceae *	*Tubaria confragosa*	AFTOL-ID 498	USA, Washington	DQ267126	AY700190	–	DQ447944	DQ408113	AY665776	Matheny et al. 2007

For the multigene phylogenetic analyses, sequences were first checked in BioEdit v7.0.4 (Hall 2007). Alignments were generated using MUSCLE (Edgar 2004) for each region separately and then adjusted manually to remove ambiguous regions. The multigene matrix of *Agaricineae* included 123 ITS (685 bp), 116 LSU (931 bp), 52 SSU (1,046 bp), 60 *tef*1 (385 bp), 43 *rpb*1 (1,205 bp), and 66 *rpb*2 (705 bp) sequences. ModelFinder v2.2.0 (Kalyaanamoorthy et al. 2017) was used to select the best-fit partition model (edge-unlinked) based on the Akaike information criterion. In the *Agaricineae* multigene matrix, the best-fit models for each gene were SYM+I+G4 (*rpb*1), GTR+F+I+G4 (*tef*1), GTR+F+I+G4 (*rpb*2), GTR+F+I+G4 (ITS), and GTR+F+I+G4 (LSU and SSU). Bayesian inference (BI) analysis was performed in MrBayes v3.1.2. Ten million generations were run with six Markov chains and sampled every 100 generations, resulting in 100,000 trees. Burn-in was determined using Tracer v1.6, with effective sample size values greater than 200 (http://tree.bio.ed.ac.uk/software/tracer). The remaining trees were used to calculate Bayesian posterior probabilities (PP). Maximum likelihood (ML) analysis and bootstrap value estimation were performed in raxmlGUI v1.5b1 using the GTRGAMMA model with 1,000 replicates (Silvestro and Michalak 2012). The phylogenetic tree is presented in Fig. 2.

### ﻿Molecular clock analyses

Divergence time estimates based on the phylogenomic tree were obtained using penalized likelihood analyses with a truncated Newton optimization algorithm implemented in r8s v1.81 (Sanderson 2003). A fossil calibration point for *Nidulariaceae* was applied, with a minimum age constraint of 45 Myr and a maximum age constraint of 90 Myr (Varga et al. 2019). In the six-gene-based maximum clade credibility (MCC) analyses of *Panaeolus* s.l., 38 described species were included. The multigene matrix comprised 127 samples, including 118 ITS (476 bp), 85 LSU (889 bp), 51 SSU (1,032 bp), 56 *tef*1 (376 bp), 64 *rpb*1 (1,322 bp), and 65 *rpb*2 (682 bp) sequences. An XML file was generated using BEAUti 2 (Bouckaert et al. 2014). Site models for each gene were selected using the BEAST Model Test in BEAUti 2. A Yule model was selected as the prior, assuming a constant speciation rate per lineage. A relaxed log-normal clock model was used, specifying a gamma distribution for the ucld.mean parameter with a shape of 1.0, a scale of 0.001, and an offset of 0 (Zhao et al. 2016). A second calibration category was applied, and a normal distribution prior (SD = 1) with a mean age of 87 Myr, inferred from the phylogenomic dating analyses, was assigned to the root height. An independent Markov chain Monte Carlo analysis of 150 million generations was run in BEAST v2.0 (Bouckaert et al. 2014), with log states recorded every 1,000 generations. The ultrametric MCC tree was summarized using TreeAnnotator v2.4.7, discarding 20% of states as burn-in and annotating clades with posterior probabilities ≥ 0.8. SH-aLRT and UFBoot values were estimated using PhyloSuite (Zhang et al. 2020). The MCC tree is presented in Fig. 3.

### ﻿Detecting psilocybin by HPLC–MS

A total of 0.02 g of dried mushroom specimen was used for detection. One milliliter of methanol was added, vortexed to mix, and the mixture was soaked overnight. Ultrasonic extraction was performed for 30 minutes at 25 °C. The mixture was centrifuged at 10,000 rpm for 10 minutes at 4 °C, and the supernatant was collected. An additional 0.5 mL of methanol was added to the residue and vortexed to mix. Ultrasonic extraction was performed again for 15 minutes. The extract was centrifuged at 10,000 rpm for 10 minutes at 4 °C, and the supernatants were combined. After thorough shaking, the combined extract was filtered through a 0.22 μm membrane. A 600 μL aliquot was transferred into liquid-phase vials for analysis. The presence of neurotoxins (psilocybin and baeocystin; purity > 95%, Cayman Chemical, USA) was evaluated by HPLC–MS, which was carried out using a Waters ACQUITY I-Class HPLC system coupled with a Waters Xevo-G2-XS TOF MS system (Waters, USA) under the conditions shown in Table 3. The mass range was set to 50–1,200 Da. The flow rate was maintained at 0.4 mL/min. The mobile phase solvents were water (A) and acetonitrile (B), and gradient elution was performed as follows: 0.0–0.2 min, 5% B; 0.2–5.2 min, 5–10% B; 5.2–12.5 min, 10–100% B; 12.5–13.5 min, 100% B; and 13.5–15.0 min, 100–5% B. The HPLC–MS chromatograms of the detected species are provided in the Suppl. materials 1–9.

**Table 3.** Instrument parameters for the UPLC-MS/MS analyses.

Compound	Q1 mass (Da)	Q2 mass (Da)	RT (min)
Psilocybin	285.1021	205.1326, 58.0650	1.06
Baeocystin	271.0857	–	0.94

### ﻿Phylogenetic results

**Figure 1.**
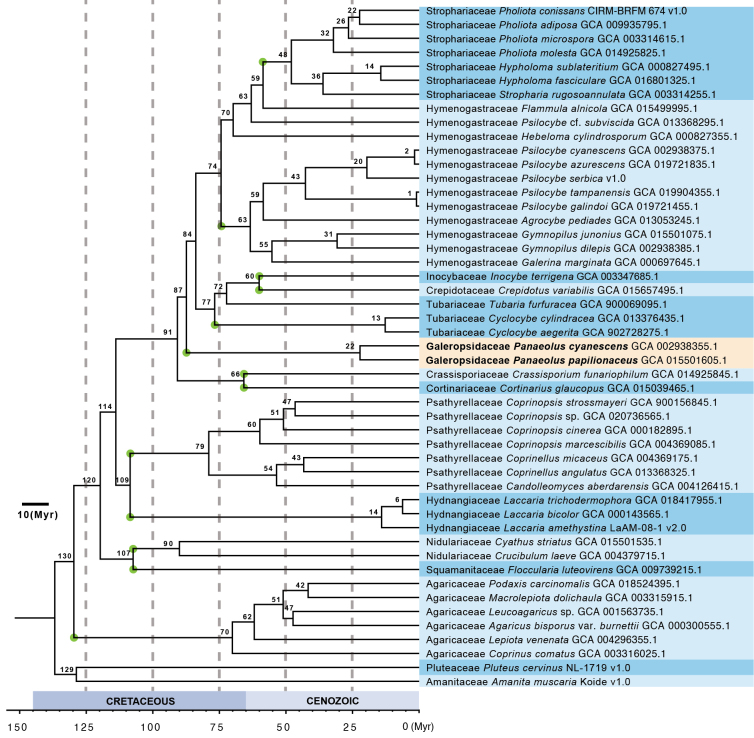
Phylogenomic tree of Agaricineae based on 1,764 single-copy, full-length BUSCO genes. The tree is rooted with *Amanita muscaria* and *Pluteus cervinus*. All nodes are fully supported by bootstrap values. Divergence times are indicated near the nodes. Green points indicate the mean stem ages of the families.

The phylogenomic tree of *Agaricineae* is presented in Fig. 1. The analysis included 49 species: 47 species representing 13 families within *Agaricineae* and two outgroup species from *Pluteineae*, namely *Amanita muscaria* (L.) Lam. and *Pluteus cervinus* (Schaeff.) P. Kumm. Divergence times of *Agaricineae* were estimated using the r8s program based on the maximum likelihood (ML) phylogenomic tree, calibrated at a node within *Nidulariaceae*. All nodes are fully supported by bootstrap values, and divergence times are indicated near the nodes. Familial relationships are largely congruent with previous studies (Zhao et al. 2017; Li et al. 2020; Wang et al. 2023), except for the sister relationship between *Hydnangiaceae* and *Psathyrellaceae* recovered here, which contrasts with Dentinger et al. (2016). Five families (*Agaricaceae*, *Hydnangiaceae*, *Nidulariaceae*, *Psathyrellaceae*, and *Strophariaceae*) were well resolved as monophyletic, with stem ages ranging from 59 to 130 Myr. *Panaeolus cyanescens* and *P. papilionaceus* formed the panaeo-clade. This clade diverged at 87 Myr, contemporaneous with the origins of other *Agaricineae* families.

**Figure 2.**
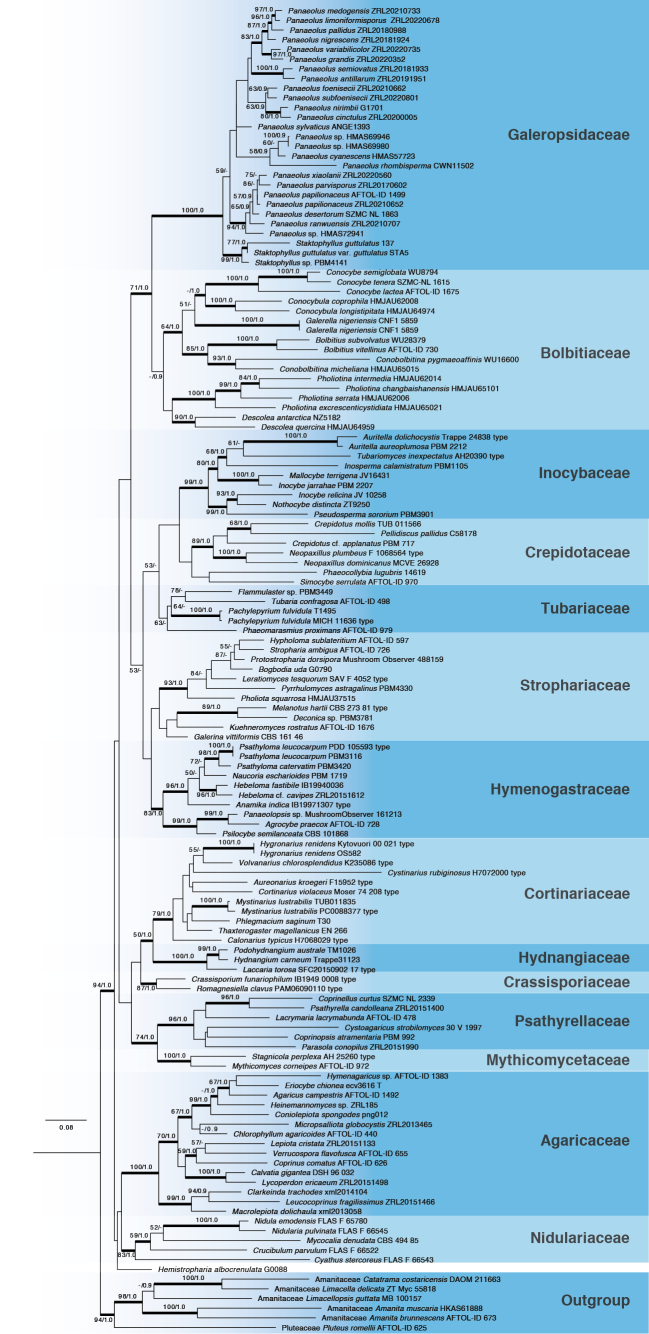
Maximum likelihood (ML) tree of Agaricineae based on six genes. Bootstrap values and Bayesian posterior probabilities greater than 50%/0.9 (BS/PP) are indicated at the nodes. Bold branches indicate PP > 0.95. “T” refers to sequences from type specimens.

An extended sampling was applied in the multigene (ITS, LSU, SSU, *rpb*1, *rpb*2, and *tef*1) ML phylogenetic analysis of *Agaricineae* to further explore the phylogenetic relationships between the panaeo-clade and other families. The analysis included 137 samples representing 125 species from 14 families within *Agaricineae*. The panaeo-clade, comprising 21 species from two genera, formed a clade sister to *Bolbitiaceae*, with statistical support (BS/PP = 71/1.0; see Fig. 2).

**Figure 3.**
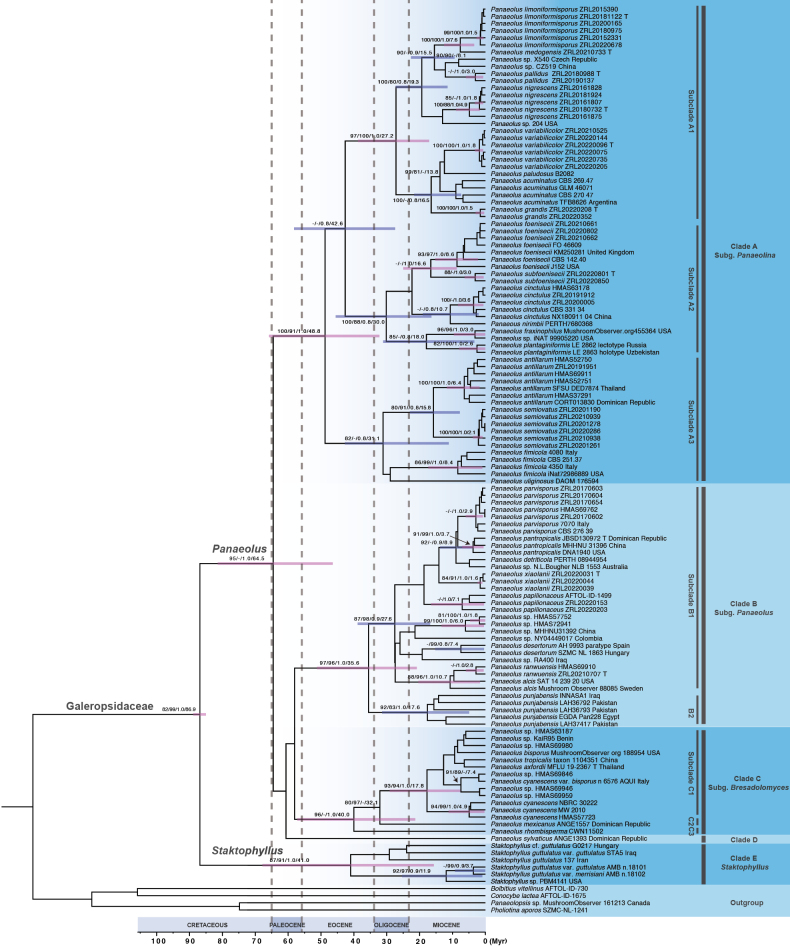
Maximum clade credibility (MCC) tree of *Galeropsidaceae* based on six-gene sequences. Values noted on the branches represent SH-aLRT/UFBoot/PP/divergence time. Pink branch bars indicate clades that are fully supported by Bayesian posterior probability. “T” refers to sequences from type specimens.

The MCC tree for *Panaeolus* s.l. is presented in Fig. 3. Five main clades (A–E) received strong support (SH-aLRT/UFBoot/PP values higher than 80/90/0.95). Three main clades roughly correspond to the traditional circumscription of subgenera in Panaeolus s.l., namely clade A corresponding to subg. Panaeolina, clade B corresponding to subg. Panaeolus, and clade C corresponding to subg. Copelandia. The remaining two clades are clade D, represented by a single sample of *Panaeolus sylvaticus*, and clade E, composed of *Staktophyllus*. Main clades A–D all have a stem age of 64.5 Myr. The divergence time of *Panaeolus* s.l. estimated in this study is roughly the same as that reported by Bradshaw et al. (2023). Subclades were delimited within each main clade to discuss phylogenetic relationships among species. Each subclade is supported by statistical values (SH-aLRT/UFBoot/PP values higher than 80/90/0.95). Thirty-three known species are phylogenetically recognized.

## ﻿Taxonomy

### ﻿ Galeropsidaceae

Taxon classificationAnimaliaAgaricalesGaleropsidaceae

Singer, Boletín de la Sociedad Argentina de Botánica 10: 61 (1962)

B57F6A8E-A22E-51F8-8B9B-CDFC32463717

#### Type.

*Galeropsis* Velen. 1930 [current name: *Panaeolus* (Fr.) Quél.]

#### Description.

Basidiomes small- to medium-sized, agaricoid or sequestrate. Spore print black. Pileus ovoid, parabolic, conic, campanulate, rarely convex and plane; white, grey, black, brown, rarely orange; dry, viscid, or glutinous, hygrophanous, with hygrophanous spots, and streaks, or non- hygrophanous. Lamellae crowded to subdistant, adnate, gray to black, variegated. Stipe equal, smooth, glabrous, pruinose, annulus membranous, rarely remaining still mature. Basidiospores brown, limoniform, ellipsoid to elongate, germ pore distinctive, smooth, punctate, verrucose. Cystidia abundant, this include caulocystidia, cheilocystidia, pleurocystidia and pileocystidia.

#### Habitat.

Grows in fertile areas, including pastures, grasslands, forests, and on dung of herbivores.

#### Genera included.

*Panaeolus*, *Staktophyllus*.

#### Notes.

The panaeo-clade was revealed to be monophyletic in previous studies (Moncalvo 2002; Walther 2005; Matheny et al. 2006) and is also supported as monophyletic in this study. With extended sampling, our study further supports ranking the panaeo-clade as a distinct family within *Agaricineae* based on the following evidence: first, it is monophyletic and occupies a phylogenetic position sister to *Bolbitiaceae* within *Agaricineae* (see Figs 1, 2); second, it diverged at 87 Myr, which is relatively similar to divergence times of other families in *Agaricineae*; third, it differs from other families in *Agaricineae* in a combination of morphological characteristics, including a saprotrophic lifestyle, black spore print, variegated lamellae, and dark brown basidiospores with a distinctive germ pore and a relatively thick wall. According to Art. 11.5, priority of equally published names is established by the first effectively published choice; therefore, the panaeo-clade should be named *Galeropsidaceae* rather than *Panaeolaceae* (Kalichman et al. 2020). Currently, *Galeropsidaceae* contains two genera, *Panaeolus* and *Staktophyllus*.

### ﻿ Panaeolus

Taxon classificationAnimaliaAgaricalesGaleropsidaceae

(Fr.) Quél., Mém. Soc. Émul. Montbéliard, Sér. 2 5: 151 (1872)

CC7B5A68-2898-541B-9B00-5D5238921E9C

#### Basionym.

Agaricus subg. Panaeolus Fr.

#### Type species.

*Panaeolus papilionaceus* (Bull.) Quél.

#### Description.

the same as description of *Galeropsidaceae*.

#### Notes.

The morphological synapomorphy of *Panaeolus* is a relatively small basidiome with variegated lamellae and blackish brown basidiospores. All known species are saprotrophic, and many of them are coprophilous. It is a worldwide genus known from all continents except Antarctica. The psilocybin-producing trait is known to be distributed in subg. Bresadolomyces and subg. Panaeolina. Based on the results of the phylogenetic analyses, *Crucispora* is synonymized with *Panaeolus* and placed in subg. Bresadolomyces. Three subgenera of *Panaeolus* are recognized based on phylogenetic evidence.

#### Subgenera included.

subg. Bresadolomyces, subg. Panaeolina, subg. Panaeolus.

### ﻿ Panaeolus subg. Bresadolomyces

Taxon classificationAnimaliaAgaricalesGaleropsidaceae

M.Q. He & R.L. Zhao subg. nov.

F3B206B9-078C-5A35-9054-195CB4B655EA

860264

#### Diagnosis.

Species of this subgenus have relatively thin-fleshed, dirty whitish or gray to grayish-brown pigmented basidiome, some species turning blue or bluish-gray when get injured; basidiospores smooth, ellipsoid, limoniform, cruciform-rhomboid.

#### Type species.

*Panaeolus cyanescens* Sacc.

#### Etymology.

Named in honor of Giacomo Bresàdola (1847–1929) for his research contributions to agarics taxonomy and introduction of *Copelandia*.

#### Synonyms.

*Copelandia* Bres., Hedwigia 53(1–2): 51 (1912) [1913] (nom. illeg., Shenzhen Code, Art. 52.1); Panaeolus subgen. Copelandia (Bres.) Ew. Gerhardt, Biblioth. Bot. 147: 32 (1996) (nom. illeg., Art. 52.1). – Type: *Copelandia papilionacea* (Bull.) Bres., Hedwigia 53(1–2): 51 (1912) [1913] (nom. inval., Art. 35.1); *Crucispora* E. Horak, New Zealand J. Bot. 9(3): 489 (1971).

#### Description.

Basidiome relatively thin-fleshed, dirty whitish or gray to grayish-brown pigmented, some species turning blue or bluish-gray when cut or bruised; the cap surface not slimy; basidiospores smooth, ellipsoid, limoniform, or cruciform-rhomboid; the hymenium always contains thick-walled pseudocystidia (metuloids) that often secrete crystals at the tip.

#### Notes.

The synonym name subg. Copelandia was proposed with the type *P. papilionaceus* (Gerhardt 1996). However, *P. papilionaceus* had already been designated as the type of Panaeolus subg. Panaeolus by Quélet (1872). According to the Shenzhen Code, Art. 52.1, Panaeolus subg. Copelandia is illegitimate. Subgenus Bresadolomyces is proposed mainly based on phylogenetic evidence to accommodate Copelandia-like species and species formerly placed in *Crucispora*. Copelandia-like species cluster together with *Cr. rhombisperma* in clade C, which is well supported statistically (SH-aLRT/PP = 96/1.0). Copelandia-like species are mainly characterized by grayish, relatively fragile basidiomes that turn blue when injured or bruised. They represent the main psilocybin-producing group within *Panaeolus*, with the most well-known species being *P. cyanescens*.

#### Species included.

*P. axfordii* Y.W. Hu, Karun., P.E. Mortimer & J.C. Xu, *P. bisporus*, *P. cyanescens* (Guzmán) Voto & Angelini, *P. mexicanus*, *P. rhombispermus* (Hongo) Birkebak, Voto & Ostuni.

### ﻿ Panaeolus cyanescens

Taxon classificationAnimaliaAgaricalesGaleropsidaceae

Sacc., Syll. Fung. 5: 1123 (1887).

8E66B8DD-ACB0-51E2-9BE0-4E7A33FB56E9

#### Notes.

Description, see Gerhardt (1996). *Panaeolus cyanescens* is a well-known species recognized for its psilocybin-producing trait. It is a relatively easy-to-recognize species in the wild because its white-to-gray basidiomes turn blue when wounded or touched. In the phylogenetic analyses, this species is placed in subclade C1, where it shows a sister relationship to the clade composed of *P. axfordii*, *P. tropicalis*, *P. bisporus*, and several unnamed samples.

#### Specimens examined.

China. Guizhou Province: Qiannan Buyi and Miao Autonomous Prefecture, Libo County, 4 August 1988, Jian-Zhe Ying, Chen-Liu Zong, and Ning Li, HMAS57723.

### ﻿ Panaeolus subg. Panaeolina

Taxon classificationAnimaliaAgaricalesGaleropsidaceae

(Maire) Bon & Courtec., Doc. Mycol. 32 (127–128): 77 (2003)

E5ADCC04-C5DC-5BF9-B219-B2A1E87DFFDD

#### Type species.

*Panaeolus foenisecii* (Pers.) J. Schröt.

#### Description.

The same as *Galeropsidaceae*.

#### Notes.

Species of subg. Panaeolina are clustered in clade A, which is a well-supported clade with nearly full statistical support (SH-aLRT/UFBoot/PP = 100/91/1.0). Based on the phylogenetic results, a broader circumscription of subg. Panaeolina is proposed. It includes three subclades, namely subclade A1, comprising most species from China; subclade A2, with the type species *P. foenisecii*, which represents the core clade of subg. Panaeolina; and subclade A3, including Anellaria-like species. In the sense of *Panaeolus* s.s., *Panaeolina* has been treated as a distinct genus. The off-black spore print has been used to separate *Panaeolina* from other genera (Singer 1986; Gerhardt 1996). However, determining a paler spore print is relatively difficult and subjective, especially in the field. Verrucose basidiospores have been considered a key character of *Panaeolina*; however, at present, only the type species, *P. foenisecii*, has been observed to possess verrucose basidiospores, whereas all other species form smooth basidiospores.

#### Species included.

*P. acuminatus* (P. Kumm.) Quél., *P. antillarum* (Fr.) Dennis, Kew Bull., *P. cinctulus* (Bolton) Sacc., *P. fimicola* (Pers.) Gillet, *P. foenisecii*, *P. fraxinophilus* A.H. Sm., *P. grandis* M.Q. He & R.L. Zhao, *P. limoniformisporus* M.Q. He & R.L. Zhao, *P. medogensis* M.Q. He & R.L. Zhao, *P. nigrescens* M.Q. He & R.L. Zhao, *P. nirimbii* (Watling & A.M. Young) Voto, *P. pallidus* M.Q. He & R.L. Zhao, *P. paludosus* Cleland, *P. plantaginiformis* (Lebedeva) E.F. Malysheva, *P. semiovatus*, *P. subfoenisecii* M.Q. He & R.L. Zhao, *P. uliginosus* Jul. Schäff., *P. variabilicolor* M.Q. He & R.L. Zhao.

### ﻿ Panaeolus antillarum

Taxon classificationAnimaliaAgaricalesGaleropsidaceae

(Fr.) Dennis, Kew Bull. 15(1): 124 (1961)

9929ED48-2D0A-5BD8-A547-50C329C0FFA1

Fig. 4

#### Basionym.

*Agaricus antillarum* Fr.

#### Description.

*fide* Desjardin and Perry (2017).

#### Psilocybin-producing.

Nonproducing (HMAS52750, see Suppl. material 1: fig. S1).

#### Notes.

*Panaeolus antillarum* was first described from St. Croix in the Greater Antilles (U.S. Virgin Islands). Later, it was found to be a worldwide species distributed in the New World (Caribbean islands, continental United States, Central and South America) and the Old World (Africa, Australia, China, Europe, India, and Southeast Asia) (Desjardin and Perry 2017).

**Figure 4.**
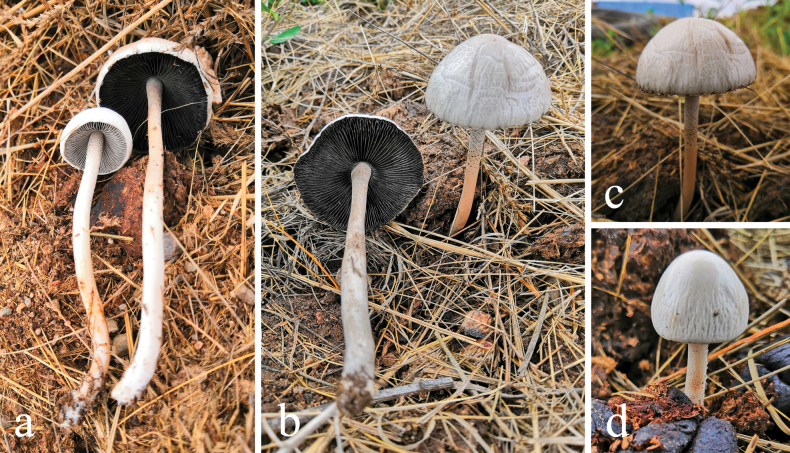
Basidiomes of *Panaeolus antillarum* in the field.

#### Specimens examined.

China. Xizang Autonomous Region: Nyingchi Municipality, Mêdog County, 27 July 1983, Xiao-Lan Mao, HMAS52750; 3 August 1983, Xiao-Lan Mao, HMAS52751. Hebei Province: Zhangjiakou, August 1994, Xiao-Lan Mao and You-Zhi Wang, HMAS69911; Bejing: Zhongguancun, 27 September 1976, Shu-Xiao Sun, HMAS37291; Inner Mongolia Autonomous Region: Xilin Gol League, 11 August 2019, Jian-Yu Zhang, *ZRL20191951*.

### ﻿ Panaeolus cinctulus

Taxon classificationAnimaliaAgaricalesGaleropsidaceae

(Bolton) Sacc., Syll. fung. (Abellini) 5: 1124 (1887)

5726125B-FBF7-58B2-9723-E8EA8D4B558E

Fig. 5

#### Basionym.

*Agaricus cinctulus* Bolton 1792

#### Description.

Pileus 7.0–26.5 mm in diam., parabolic, obtusely conic, gray (pantone warm gray 1 c), light brown (pantone 4665 c), glabrous, surface dry, smooth, disc could be darker as light brown, margin straight or slightly decurved. Lamellae adnate, close, broad, mottled grayish to blackish, entire, white edge not distinctive. Stipe equal, hollow, 42.1–75.3 mm long, 1.4–2.6 mm thick, the same color as pileus, smooth, surface silk-like, longitudinally striate, especially on the upper side, base with whitish mycelium. Basidiomes getting brown when handling, bruising, and cutting.

Basidiospores 11.3–12.5 × 7.8–9.2 μm, [x = 11.8 ± 0.3 × 8.7 ± 0.3, Q = 1.3–1.5, Q_m_ = 1.4 ± 0.1, n = 20], ellipsoid, blackish brown when mature, smooth, thick wall with germ pore. Basidia 20.7–25.8 × 9.7–12.8 μm, 4-spored, smooth, hyaline. Cheilocystidia 23.7–41.5 × 7.2–10.5 μm, narrowly clavate, with a slightly inflated head and base, thin wall, hyaline, smooth. Pleurocystidia absent. Pileocystidia not observed. Caulocystidia 14.0–46.3 × 4.0–9.3 μm, cylindrical, hyaline, slightly flexuose.

**Figure 5.**
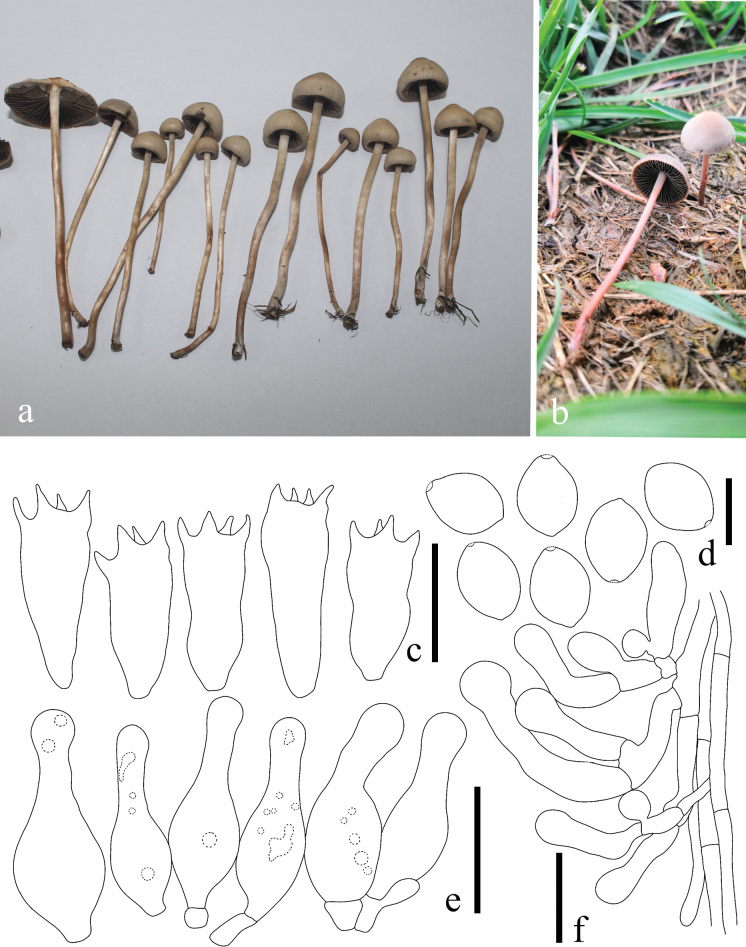
*Panaeolus cinctulus*. **a, b** basidiomes in the field; **c** basidia; **d** basidiospores; **e** cheilocystidia; **f** caulocystidia. Scale bars: 20 μm (**c**, **e**, **f**); 10 μm (**d**).

#### Habitat.

Grows in grassland.

#### Psilocybin-producing.

Producing (*ZRL20200005*, see Suppl. material 1: fig. S7).

#### Notes.

According to the results of the phylogenetic analyses, *P. cinctulus* is clustered in subclade A2 and is sister to the sequestrate species *P. nirimbii*. Morphologically, our samples exhibit smaller basidiomes (pileus 7.0–26.5 mm in diam.) and a paler pileus compared with the original description of *P. cinctulus* (Saccardo 1887) and the description provided by Stamets (1996).

#### Specimens examined.

China. Inner Mongolia Autonomous Region: Baotou, Olympics Park, 13 June 2019, Jian-Yu Zhang, *ZRL20191912*; Beijing: Chaoyang District, Olympics Park, 19 July 2020, Rui-Lin Zhao, *ZRL20200005*; Ningxia Hui Autonomous Region: Yinchuan, 11 September 1995, Xiaolan Mao, Cangkuan Wang, HMAS63178.

### ﻿ Panaeolus foenisecii

Taxon classificationAnimaliaAgaricalesGaleropsidaceae

(Pers.) J. Schröt., Botaniste 17(1–4): 187 (1926)

FA0155BC-5249-5E52-919A-571FF7956DBB

Fig. 6

#### Basionym.

*Agaricus foenisecii* Pers. 1800

#### Description.

Pileus 7.6–17.6 mm in diam., parabolic, light brown (pantone 4685 c), brown (pantone 4645 c), glabrous, surface dry, hygrophanous especially at the margin, smooth, margin straight or slightly decurved. Lamellae adnate, subdistant, broad, mottled grayish to blackish, entire, edge white. Stipe equal, hollow, 24.1–47.3 mm long, 1.6–2.6 mm thick, brown, the same color as pileus, smooth, longitudinally striate, occasionally pruinose on the upper side, base with whitish mycelium. Basidiomes getting brown when handling, bruising, and cutting.

Basidiospores 11.3–12.5 × 7.8–9.2 μm, [x = 11.8 ± 0.3 × 8.7 ± 0.3, Q = 1.3–1.5, Q_m_ = 1.4 ± 0.1, n = 20], ellipsoid, punctate, verrucose, brown, reddish brown, thick wall with germ pore. Basidia 25.8–39.6 × 8.9–12.5 μm, 4-spored, smooth, hyaline. Cheilocystidia 28.3–61.4 × 5.1–11.4 μm, narrowly clavate, with an inflated head and base, hyaline, smooth. Pleurocystidia absent. Cuticle composed of cellular or hymeniform cell, pileocystidia 22.1–79.2 × 12.3–50.5 μm, spheropedunculate, hyaline. Caulocystidia not observed.

**Figure 6.**
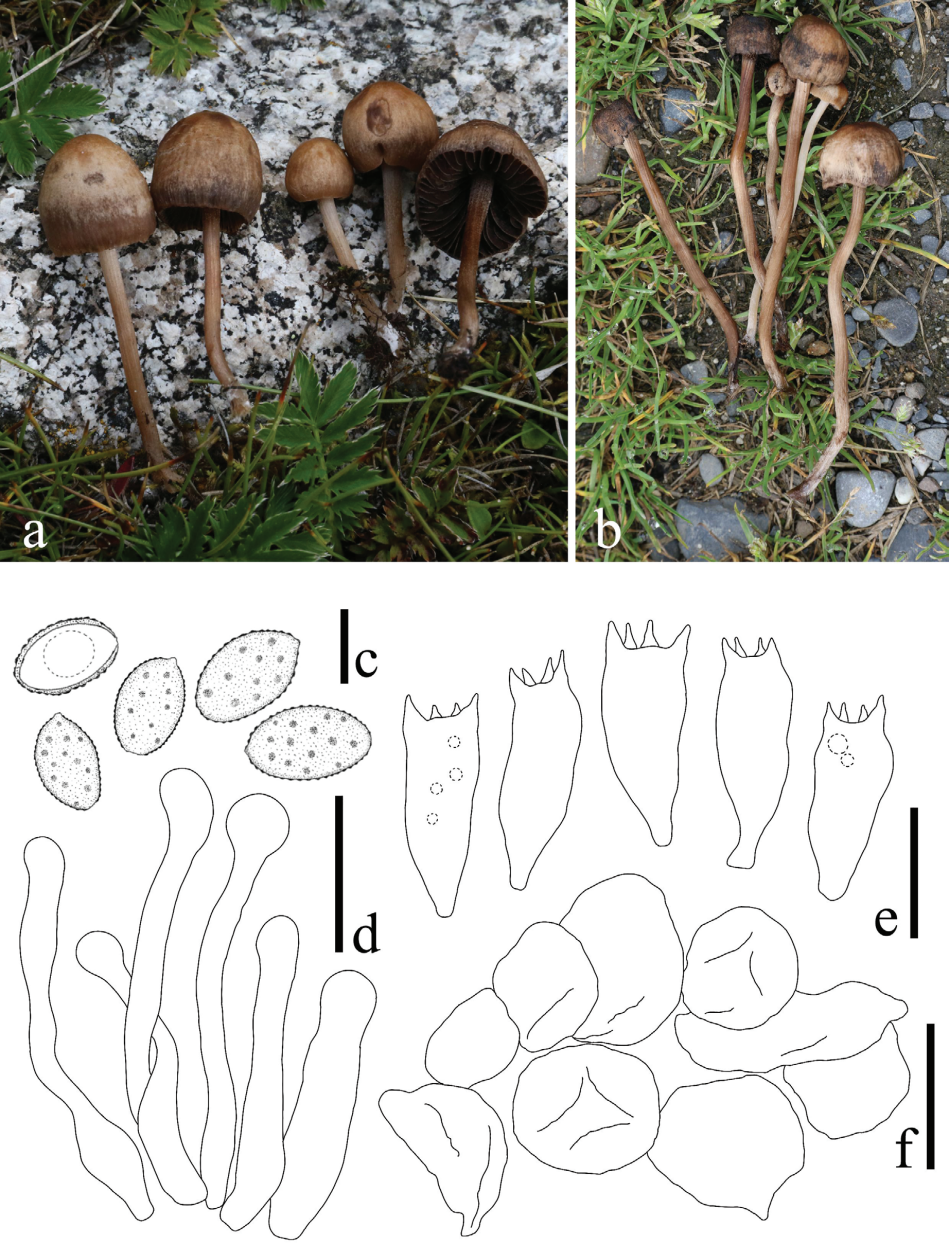
*Panaeolus foenisecii*. **a, b** basidiomes in the field; **c** basidiospores; **d** cheilocystidia; **e** basidia; **f** wilted pileocystidia. Scale bars: 10 μm (**c**); 20 μm (**d–f**).

#### Habitat.

Grows on fertile soil as pastures, grasslands, and forests.

#### Psilocybin-producing.

Nonproducing (*ZRL20210662*, see Suppl. material 1: fig. S6).

#### Notes.

*Panaeolus foenisecii* is characterized by its punctate to verrucose basidiospores.

#### Specimens examined.

China. Xizang Autonomous Region: Nyingchi Municipality, Zayü County, 29.20.1°N, 97.5.16°E, alt. 4230 m, 23 July 2021, Mao-Qiang He, *ZRL20210661*, *ZRL20210662*; Shigatse Municipality: Gyirong County, Gyirong Town, 28.22.39°N, 85.19.40°E, alt. 2780 m, 02 August 2022, Mao-Qiang He, *ZRL20220802*.

### ﻿ Panaeolus grandis

Taxon classificationAnimaliaAgaricalesGaleropsidaceae

M.Q. He & R.L. Zhao sp. nov.

05443C1A-ACFE-5DDF-AC0D-9BC23D0AC269

860267

Fig. 7

#### Etymology.

*grandis* (Lat.) refers to the relatively large basidiomes of this species in *Panaeolus*.

#### Diagnosis.

*Panaeolus grandis* has relatively large basidiome with a campanulate, brown pileus and long stipe; cheilocystidia tibiiform with an inflated base.

#### Holotype.

CHINA. Xizang Autonomous Region: Shigatse Municipality, Yadong County, 27.25.17°N, 88.56.32°E, alt. 3024 m, 26 July 2022, Mao-Qiang He, HMAS287495 (*ZRL20220208*).

#### Description.

Pileus 13.9–75.6 mm in diam., campanulate, disc light brown (pantone 3596 c), brown (pantone 2441 c), paler elsewhere (pantone 4755 c), surface dry, hygrophanous, sometimes rugulose around disc, margin straight, sometimes slightly uplifted. Lamellae adnate, subdistant, broad, mottled grayish to blackish, entire, edge white. Stipe equal, hollow, 63.8–156.8 mm long, 1.7–5.0 mm thick, light brown, the same color as pileus (pantone 4755 c), smooth, longitudinally striate, pruinose, base with whitish mycelium.

Basidiospores 12.0–13.9 × 7.1–9.6 μm, [x = 12.9 ± 0.6 × 8.1 ± 0.6, Q = 1.3–1.8, Q_m_ = 1.6 ± 0.1, n = 20], ellipsoid, broadly ellipsoid, smooth, blackish brown, thick wall with germ pore. Basidia 22.3–29.8 × 8.9–10.6 μm, 4-spored, smooth, hyaline. Cheilocystidia 25.7–41.9 × 5.3–9.3 μm, tibiiform with an inflated base, hyaline, smooth. Pleurocystidia absent. Cuticle composed of large vesicles, pileocystidia not observed. Caulocystidia 43.9–102.2 × 6.0–10.5 μm, cylindrical, hyaline, some with brown pigment inside.

**Figure 7.**
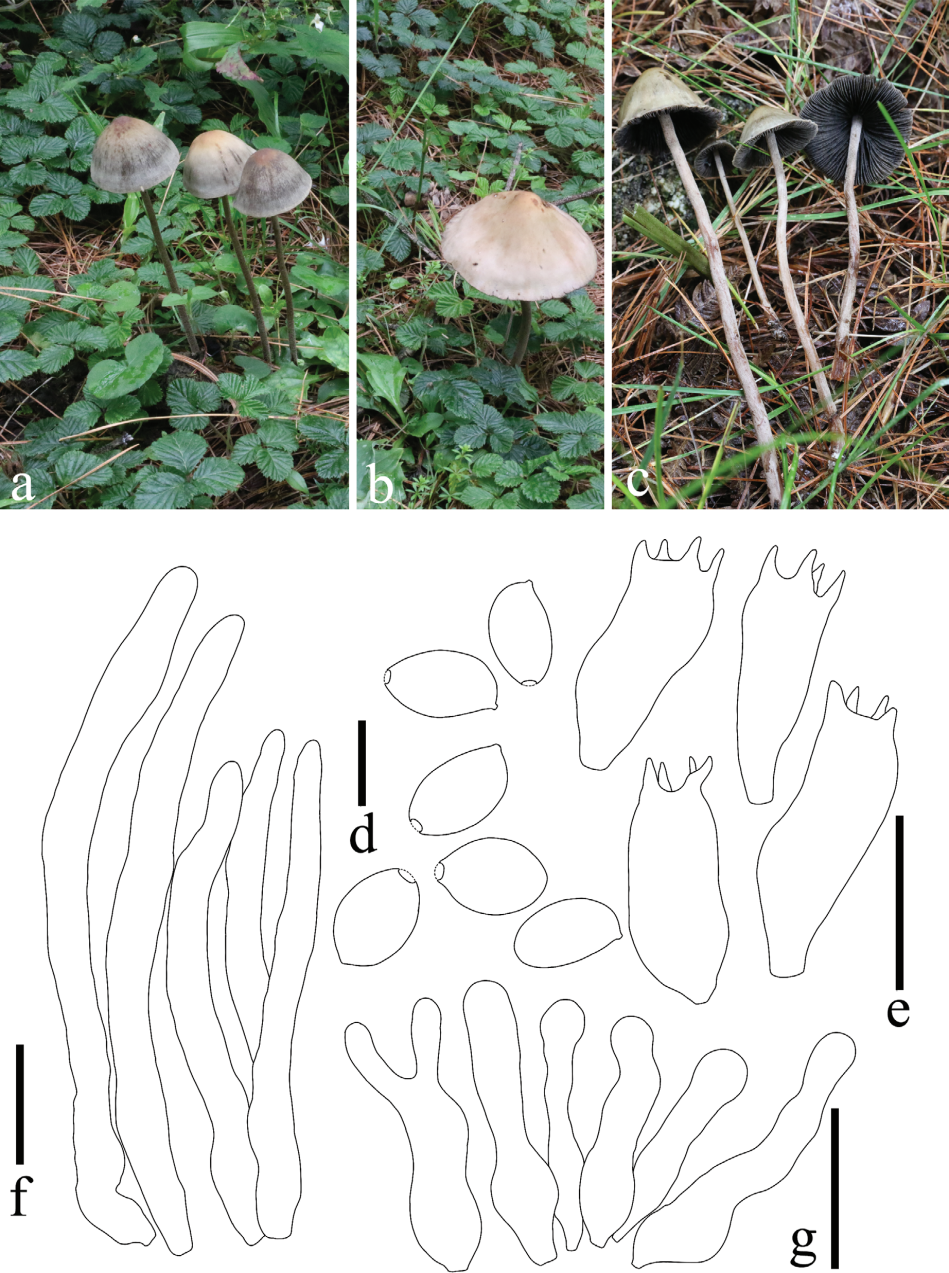
*Panaeolus grandis*. **a–c** basidiomes in the field; **d** basidiospores; **e** basidia; **f** caulocystidia; **g** cheilocystidia. Scale bars: 10 μm (**d**); 20 μm (**e–g**).

#### Habitat.

Grows on soil in grasslands, pine forests.

#### Psilocybin-producing.

Nonproducing (*ZRL20220208*, see Suppl. material 1: fig. S14).

#### Notes.

According to the results of the phylogenetic analyses, *P. grandis* is clustered in subclade A1. Morphologically, *P. grandis* differs from other species in *Panaeolus* by its relatively large-sized basidiome with a campanulate, brown pileus and a long stipe. Although *P. pallidus* also has a basidiome of similar size, it forms larger basidiospores (17.2 ± 0.6 × 11.8 ± 0.4 μm) compared with *P. grandis*.

#### Specimen examined.

China. Xizang Autonomous Region: Shigatse Municipality, Yadong County, 27.25.20°N, 88.55.6°E, alt. 3254 m, 27 July 2022, Mao-Qiang He, *ZRL20220352*.

### ﻿ Panaeolus limoniformisporus

Taxon classificationAnimaliaAgaricalesGaleropsidaceae

M.Q. He & R.L. Zhao sp. nov.

192DA34D-2025-593B-9CC4-0DD45BE91E2D

860268

Fig. 8

#### Etymology.

*limoniformisporus* (Lat.) refers to the limoniform basidiospores.

#### Diagnosis.

*Panaeolus limoniformisporus* has gray basidiomes with conic, hygrophanous pileus and limoniform basidiospores.

#### Holotype.

CHINA. Gansu Province: Wuwei, Tianzhu County, Kela, 19 August 2018, Zhi-Lin Ling, HMAS287497 (*ZRL20181122*).

#### Description.

Pileus 13.1–39.9 mm in diam., conic, parabolic, background gray (pantone cool gray 1 c) to light brown (pantone 4685 c), surface dry, smooth, mottled, and radially streaked by blackish brown (pantone 7533 c) hygrophanous stripes, also could be totally white or light brown without radial stripes, margin slightly exceeding gills. Lamellae adnate, sub-close, broad, mottled grayish to blackish, entire, with a white edge. Stipe equal, hollow, 74.9–108.6 mm long, 1.9–5.0 mm thick, white, longitudinally striate, especially on the upper side, base with whitish mycelium, getting darker when handling.

Basidiospores 13.7–15.1 × 10.0–11.3 μm, [x = 14.3 ± 0.4 × 10.7 ± 0.4, Q = 1.3–1.4, Q_m_ = 1.3 ± 0.0, n = 20], limoniform, blackish brown, smooth, thick wall with germ pore. Basidia 24.1–28.9 × 12.6–14.9 μm, 4-spored, smooth, hyaline. Cheilocystidia 23.6–40.5 × 4.2–9.9 μm, narrowly cylindrical, flexuose, with a slightly inflated base, hyaline, smooth. Pleurocystidia 21.4–27.5 × 10.7–13.8 μm, spheropedunculate, hyaline. Pileocystidia not observed. Caulocystidia not observed.

**Figure 8.**
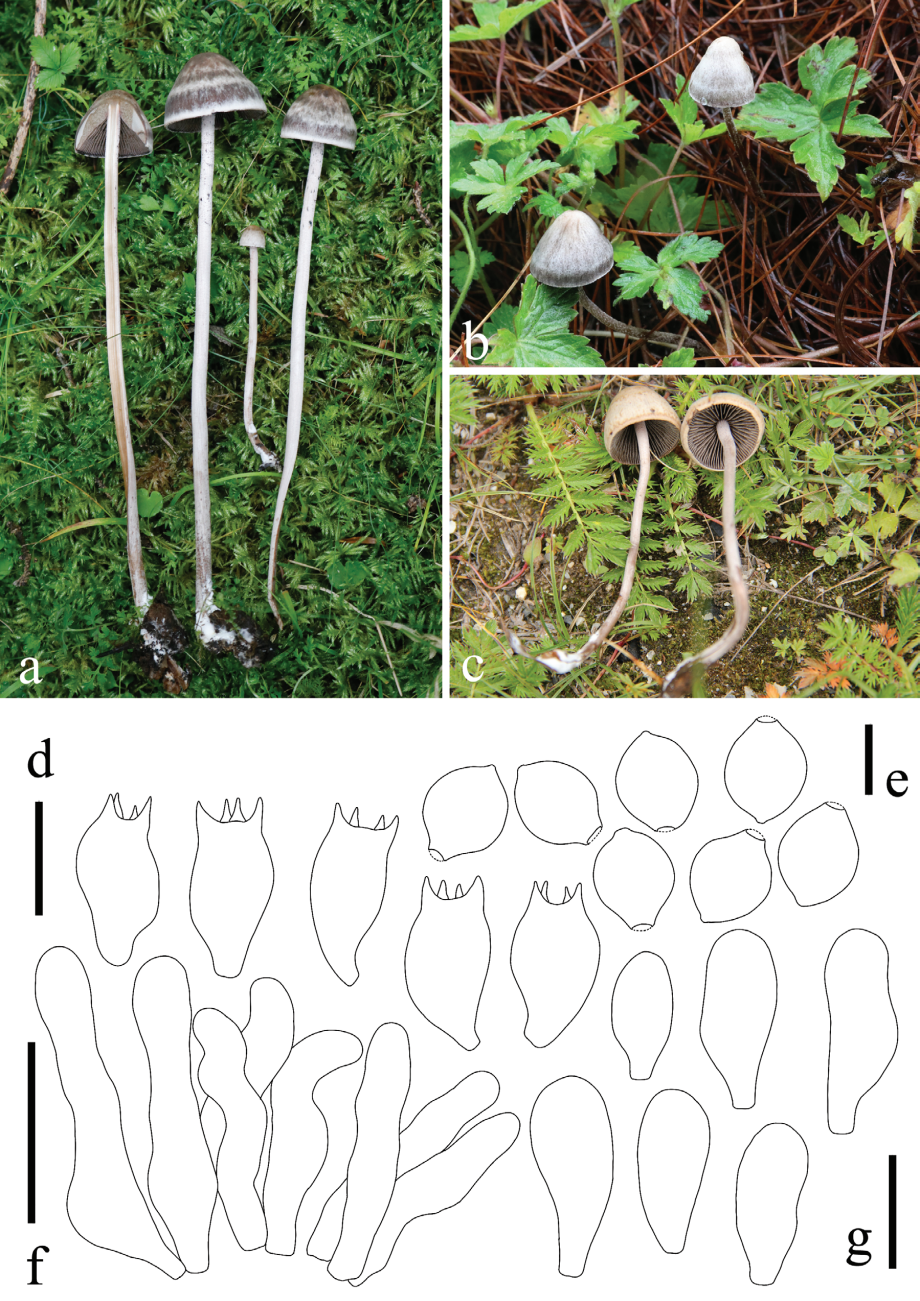
*Panaeolus limoniformisporus*. **a–c** basidiomes in the field; **d** basidia; **e** basidiospores; **f** cheilocystidia; **g** pleurocystidia. Scale bars: 20 μm (**d**, **f–g**); 10 μm (**e**).

#### Habitat.

Grows in pastures and on soil.

#### Psilocybin-producing.

Nonproducing (*ZRL20181122*, see Suppl. material 1: fig. S12).

#### Notes.

*Panaeolus limoniformisporus* is revealed to be the sister species of *P. medogensis* (Figs 2, 3). Morphologically, both species form small-sized basidiomes, but the pileus differs between them: the pileus of *P. limoniformisporus* is gray, black, or white, slightly fibrillose, and hygrophanous, whereas the pileus of *P. medogensis* is brown and glabrous.

#### Specimens examined.

China. Sichuan Province: Tibetan Autonomous Prefecture of Garzê, Batang County, 20 July 2015, Mao-Qiang He, *ZRL2015390*; Yajiang County, Gexi Natural Reserve, 15 August 2020, Mao-Qiang He, *ZRL20200165*; Xizang Autonomous Region: Nyingchi Municipality, Nang County, 19 September 2015, Xu-Ming Bai, *ZRL20152331*; Gansu Province: Wuwei, Tianzhu County, Kela, 19 August 2018, Zhi-Lin Ling, *ZRL20180975*; Shigatse Municipality, Gyirong County, Gyirong Town, 28.22.39°N, 85.19.40°E, alt. 2780 m, 02 August 2022, Mao-Qiang He, *ZRL20220678*.

### ﻿ Panaeolus medogensis

Taxon classificationAnimaliaAgaricalesGaleropsidaceae

M.Q. He & R.L. Zhao sp. nov.

5155D71B-AF1D-53EE-B7AA-1EA035BBEEFA

860269

Fig. 9

#### Etymology.

*medogensis* (Lat.) refers to the type locality, Mêdog County, in the Xizang Autonomous Region of China.

#### Diagnosis.

*Panaeolus medogensis* has small basidiomes with glabrous and rugulose pileus and narrowly clavate cheilocystidia with inflated base.

#### Holotype.

CHINA. Xizang Autonomous Region: Nyingchi Municipality, Mêdog County, 29.47.30°N, 95.41.50°E, alt. 3670 m, 24 July 2021, Mao-Qiang He, HMAS287498 (*ZRL20210733*).

#### Description.

Pileus 7.6–17.6 mm in diam., parabolic, light gray (pantone 427 c), light brown (pantone 4685 c), brown (pantone 4645 c), color radially getting paler from disc to margin, glabrous, surface dry, smooth, rugulose especially in/around the disc, margin straight or slightly decurved. Lamellae adnate, subdistant, broad, mottled grayish to blackish, entire, edge white. Stipe equal, hollow, 24.1–47.3 mm long, 1.6–2.6 mm thick, brown, the same color as pileus, longitudinally striate, some pruinose, base with whitish mycelium. Basidiomes getting brown when handling, bruising, and cutting.

Basidiospores 13.2–15.0 × 9.2–11.4 μm, [x = 13.9 ± 0.5 × 10.3 ± 0.6, Q = 1.2–1.5, Q_m_ = 1.4 ± 0.1, n = 20], ellipsoid, limoniform, brown, thick wall with germ pore. Basidia 29.7–40.0 × 11.4–14.5 μm, 4-spored, smooth, hyaline. Cheilocystidia 23.1–35.9 × 4.2–8.7 μm, narrowly clavate, with an inflated base, slightly flexuose, hyaline, smooth. Pleurocystidia 22.4–37.7 × 10.7–14.1 μm, clavate, hyaline. Pileocystidia not observed. Caulocystidia 32.0–72.2 × 3.7–11.9 μm, long clavate, hyaline, some slightly flexuose.

**Figure 9.**
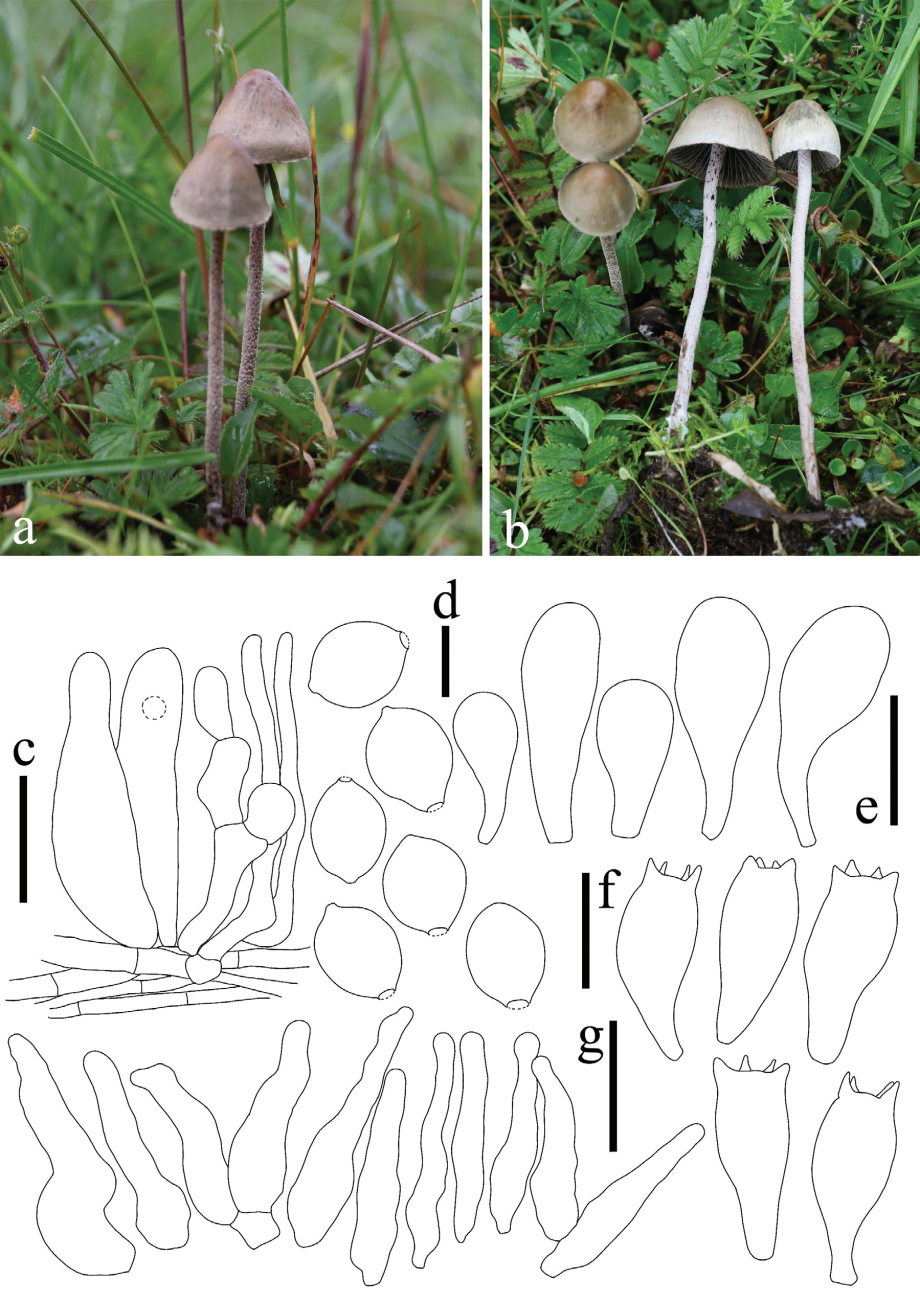
*Panaeolus medogensis*. **a, b** basidiomes in the field; **c** caulocystidia; **d** basidiospores; **e** pleurocystidia; **f** basidia; **g** cheilocystidia. Scale bars: 20 μm (**c**, **e–g**); 10 μm (**d**).

#### Habitat.

Grows on fertile soil as pastures, grasslands, and forests.

#### Psilocybin-producing.

Nonproducing (*ZRL20210733*, see Suppl. material 1: fig. S13).

#### Notes.

*Panaeolus medogensis* was found in a grazing area that is also a forest edge with lush grasses. According to the results of the phylogenetic analyses, *P. medogensis* is sister to *P. limoniformisporus*. Morphologically, the two species can be distinguished by the pileus. Compared with other species in *Panaeolus*, *P. medogensis* is characterized by its small-sized basidiomes with a brown, glabrous, and rugulose pileus.

### ﻿ Panaeolus nigrescens

Taxon classificationAnimaliaAgaricalesGaleropsidaceae

M.Q. He & R.L. Zhao sp. nov.

008786BE-1DD7-5CE1-9EA0-BF05E4A6F7C0

860270

Fig. 10

#### Etymology.

*nigrescens* (Lat.) refers to the black, grayish-black color of the pileus.

#### Diagnosis.

*Panaeolus nigrescens* has small, gray basidiomes with black hygrophanous stripes on the pileus and ellipsoid basidiospores.

#### Holotype.

CHINA. Gansu Province: Zhangye, 17 August 2018, Zhi-Ling Lin, HMAS287499 (*ZRL20180732*)

#### Description.

Pileus 6.9–28.8 mm in diam., conic, occasionally campanulate when getting mature, disc slightly umbonate or rarely flattened, background gray (pantone 421 c), disc black, hygrophanous stripes black (pantone 426c), mottled and radially streaked, surface dry, margin slightly crenate. Lamellae adnate, close, broad, mottled grayish, entire, with a white edge. Stipe equal, flexuous, hollow, 52.6–126.7 mm long, 1.8–4.1 mm thick, white, pruinose, longitudinally striate, base with whitish mycelium, getting brown when touched, cut, or bruised.

Basidiospores 10.7–14.0 × 6.5–8.7 μm, [x = 12.0 ± 0.7 × 7.6 ± 0.5, Q = 1.5–1.8, Q_m_ = 1.6 ± 0.1, n = 20], ellipsoid, blackish brown when mature, smooth, thick wall, germ pore distinctive. Basidia 19.6–26.0 × 8.8–11.1 μm, 4-spored, smooth, hyaline. Cheilocystidia 26.2–46.4 × 3.3–8.8 μm, narrowly cylindrical or flexuose, with a slightly inflated base, hyaline, smooth. Pleurocystidia absent. Cuticle composed of large vesicles, pileocystidia 12.9–46.9 × 9.4–35.5 μm, spheropedunculate, hyaline. Caulocystidia 12.1–47.3 × 5.0–10.1 μm, cylindrical or narrowly clavate, flexuose, or narrowly obovoid, hyaline, smooth.

**Figure 10.**
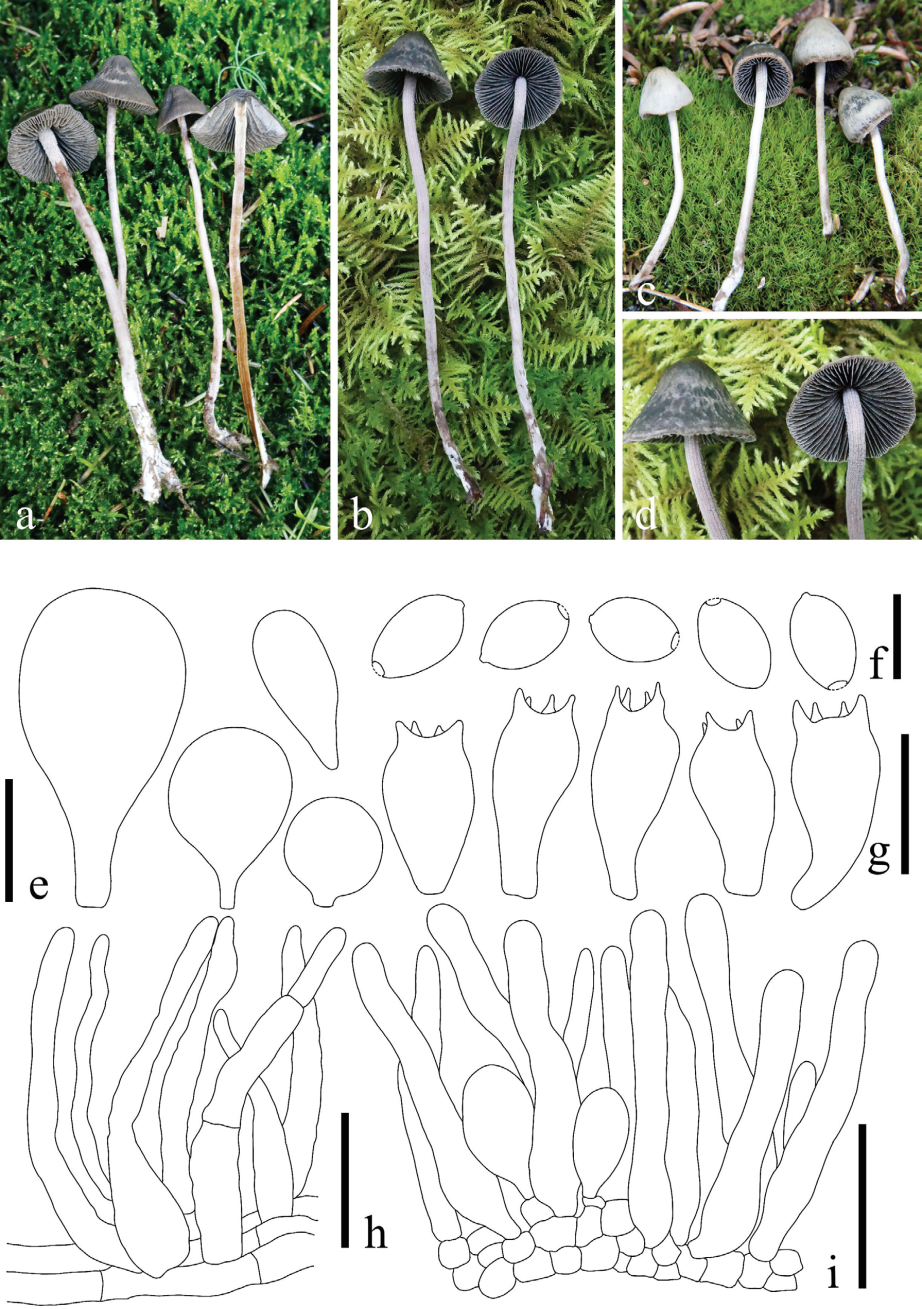
*Panaeolus nigrescens*. **a–d** basidiomes in the field; **e** pileocystidia; **f** basidiospores; **g** basidia; **h** caulocystidia; **i** cheilocystidia. Scale bars: 20 μm (**e**, **g–i**); 10 μm (**f**).

#### Habitat.

Grows on pastures and soil.

#### Psilocybin-producing.

Nonproducing (*ZRL20180732*, see Suppl. material 1: fig. S10).

#### Notes.

*Panaeolus nigrescens* is a distinctive species in subclade A1, characterized by its black or grayish black basidiomes, a pruinose stipe with longitudinal striations, and ellipsoid basidiospores. Compared with closely related species, namely *P. limoniformisporus*, *P. medogensis*, and *P. pallidus*, *P. limoniformisporus* most closely resembles *P. nigrescens* in the field. However, under the microscope, the two species can be separated by differences in basidiospore shape, with *P. nigrescens* having ellipsoid basidiospores and *P. limoniformisporus* having limoniform basidiospores.

#### Specimens examined.

China. Gansu Province: Zhangye, Qilian Mountain National Nature Reserve, Kangle Grassland, 38.384457°N, 100.653658°E, alt. 2800 m, 30 August 2016, Rui-Lin Zhao, Jean-Marc Moncalvo, *ZRL20161807*, *ZRL20161828*, *ZRL20161875*; Haichaoba Forestry Park, 26 August 2018, Ming-Zhe Zhang, *ZRL20181924*.

### ﻿ Panaeolus pallidus

Taxon classificationAnimaliaAgaricalesGaleropsidaceae

M.Q. He & R.L. Zhao sp. nov.

656C2547-15E7-594E-AE88-760F74289D8D

860266

Fig. 11

#### Etymology.

*pallidus* (Lat.) refers to the white and light color of the basidiome.

#### Diagnosis.

*Panaeolus pallidus* has relatively large basidiomes with limoniform basidiospores and narrowly cylindrical, flexuose cheilocystidia.

#### Holotype.

CHINA. Gansu Province: Wuwei, Qilian Mountain National Nature Reserve, Haxi, 22 August 2018, Mao-Qiang He, HMAS287494 (*ZRL20180988*).

#### Description.

Pileus 17.3–73.6 mm in diam., hemispheric most, also could be plane when totally mature, disc slightly umbonate or flattened, gray (pantone warm gray 1 c), light brown (pantone 467 c), sometimes hygrophanous, surface dry, smooth, occasionally rugulose, margin slightly exceeding gills. Lamellae adnate, close, broad, mottled grayish to blackish, entire, with a white edge. Stipe equal, hollow, 91.8–147.4 mm long, 3.0–5.4 mm thick, with the same color as pileus, smooth, pruinose, longitudinally striate, especially on the upper side, base with whitish mycelium.

Basidiospores 16.2–18.2 × 11.1–12.6 μm, [x = 17.2 ± 0.6 × 11.8 ± 0.4, Q = 1.4–1.5, Q_m_ = 1.5 ± 0.0, n = 20], limoniform, blackish brown when mature, smooth, thick wall, germ pore distinctive. Basidia 24.6–40.6 × 10.7–15.3 μm, 4-spored, smooth, hyaline. Cheilocystidia 36.0–61.3 × 4.6–11.4 μm, narrowly cylindrical, flexuose, with an inflated or globose head, hyaline, smooth. Pleurocystidia 29.0–33.7 × 11.1–13.6 μm, clavate to narrowly clavate, hyaline, smooth. Cuticle composed of large vesicles, pileocystidia 20.3–32.6 × 15.3–28.9 μm, spheropedunculate, hyaline. Caulocystidia 33.2–66.4 × 4.3–5.8 μm, cylindrical, rarely flexuose, hyaline, smooth.

**Figure 11.**
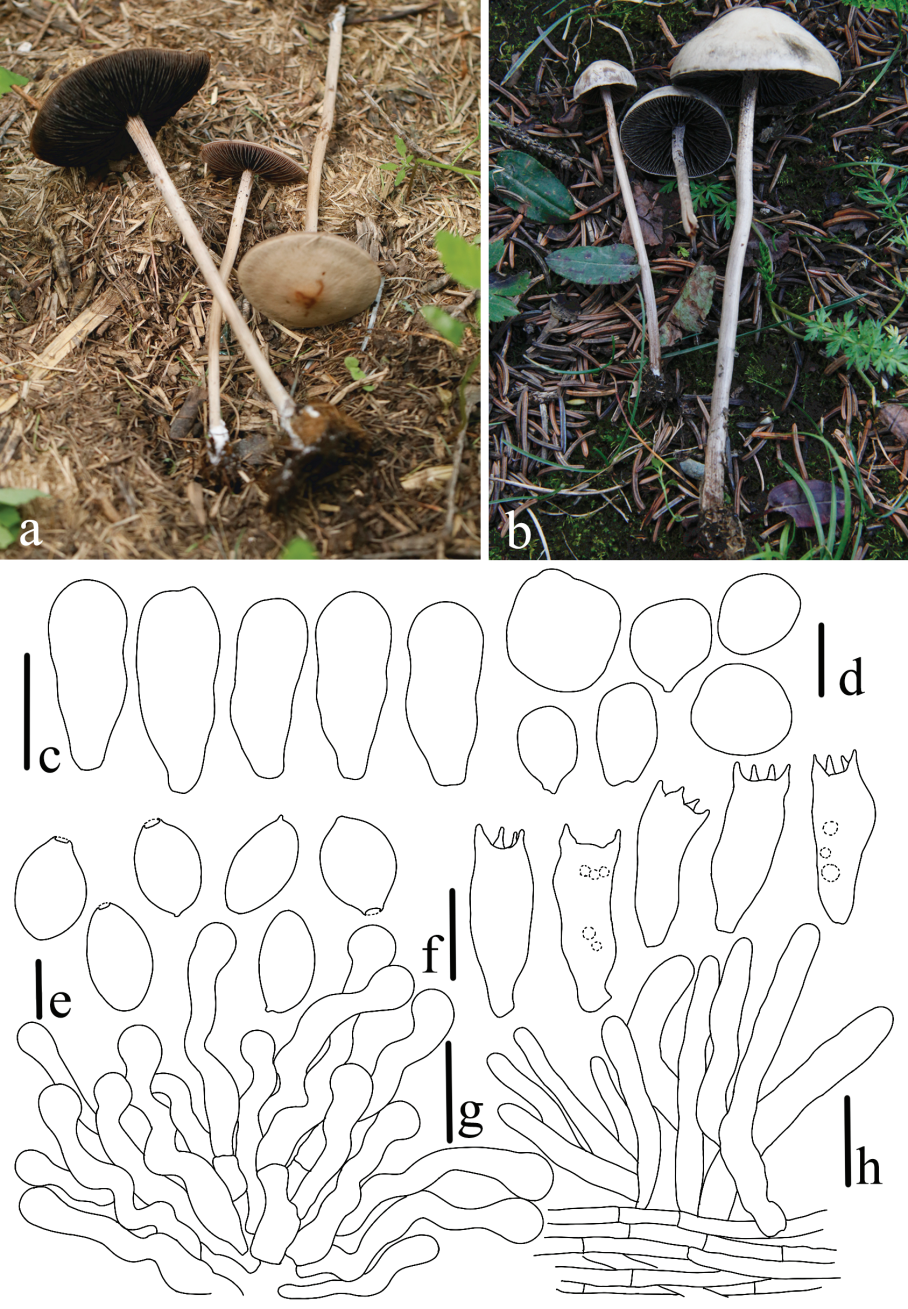
*Panaeolus pallidus*. **a, b** basidiomes in the field; **c** pleurocystidia; **d** pileocystidia; **e** basidiospores; **f** basidia; **g** cheilocystidia; **h** caulocystidia. Scale bars: 20 μm (**c, d**, **f–h**); 10 μm (**e**).

#### Habitat.

Grows in pastures and on soil.

#### Psilocybin-producing property.

nonproducing (*ZRL20180988*, see Suppl. material 1: fig. S11).

#### Notes.

In the phylogenetic tree (Fig. 3), *P. pallidus* is placed in subclade A1. The phylogenetically closest species are *P. limoniformisporus* and *P. medogensis*. *Panaeolus pallidus* was observed to have a plane pileus (observed in *ZRL20190137*), which is rarely observed in *Panaeolus*. In the field, *P. grandis* resembles *P. pallidus* in having light-colored basidiomes, but the two species differ in cheilocystidia, with *P. pallidus* having narrowly cylindrical and flexuose cheilocystidia, whereas *P. grandis* has tibiiform cheilocystidia with an inflated base.

#### Specimen examined.

China. Beijing, Fangshan District, Baicaopan Nature Park, 01 August 2019, Rui-Lin Zhao, *ZRL20190137*.

### ﻿ Panaeolus semiovatus

Taxon classificationAnimaliaAgaricalesGaleropsidaceae

(Sowerby) S. Lundell & Nannf., Fungi Exsiccati Suecici 11–12(Sched.): 14 (no. 537) (1938)

376F462B-60BF-57BD-A7C6-27411BCB0116

Fig. 12

#### Basionym.

*Agaricus semiovatus* Sowerby

#### Description.

Pileus 7–41 mm in diam., ovoid when young, then parabolic (half-egg), surface dry, smooth (could be cracked where habitat is dry), glabrous, with light color from white to bright brown (pantone 4655 c), margin straight. Lamellae adnate, close, entire, broad, mottled grayish first then becoming blackish when mature, with a white edge. Stipe equal, hollow, 17–110 mm long, 1–7 mm thick, with some color as pileus, smooth, base occasionally could be brown, always with whitish mycelium. Annulus always remaining still mature, membranous, white, medium.

Basidiospores 18.9–22.3 × 10.9–12.6 μm, [x = 20.8 ± 0.9 × 11.7 ± 0.5, Q = 1.7–2.0, Q_m_ = 1.8 ± 0.1, n = 20], ellipsoid, elongate, blackish brown when mature, smooth, thick wall, germ pore distinctive. Basidia 30.7–41.0 × 11.5–21.3 μm, 4-spored, smooth, hyaline. Cheilocystidia 26.6–39.4 × 8.1–20.8 μm, with virous shapes, clavate with a slightly narrow apex, Y-shaped, pyriform, hyaline, smooth. Pleurocystidia absent. Cuticle composed of large vesicles, pileocystidia not observed. Caulocystidia not observed.

**Figure 12.**
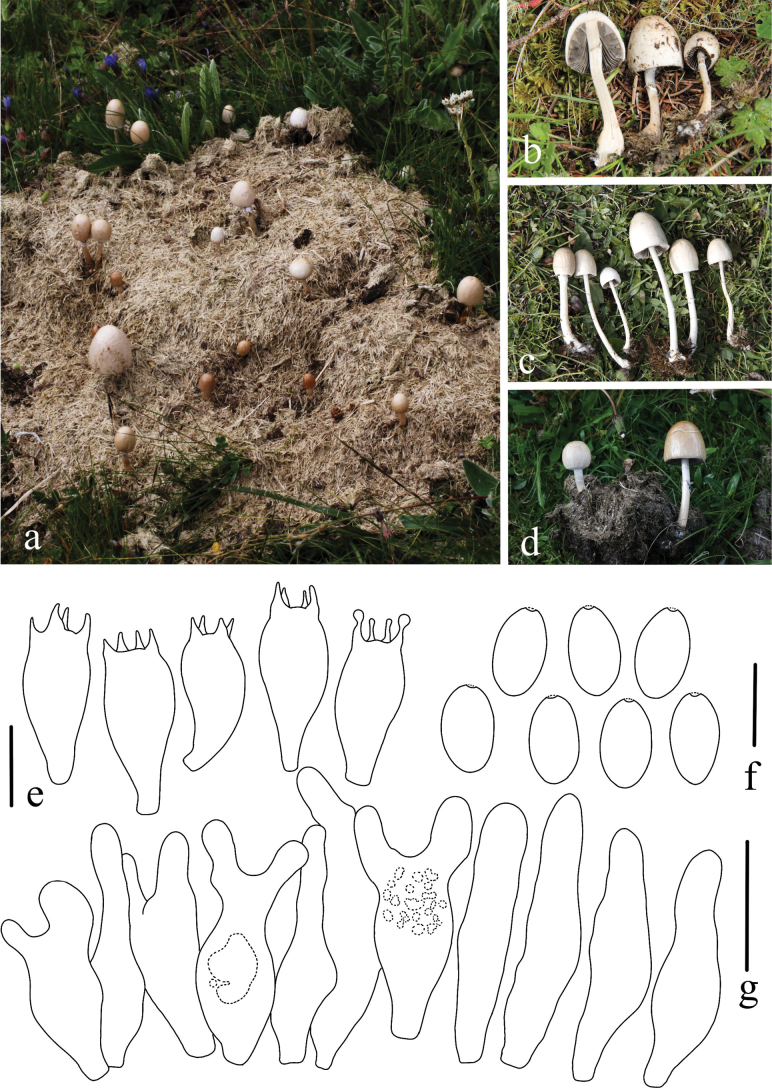
*Panaeolus semiovatus*. **a–d** basidiomes in the field; **e** basidia; **f** basidiospores; **g** cheilocystidia. Scale bars: 20 μm (**e**, **g**); 10 μm (**f**).

#### Habitat.

Grows in pastures, on dung of horses and cows.

#### Psilocybin-producing.

Nonproducing (*ZRL20201278*, see Suppl. material 1: fig. S2).

#### Notes.

*Panaeolus semiovatus* is characterized by its medium- to large-sized basidiomes, a parabolic and glabrous pileus of light color, and a permanent annulus that persists at maturity. These characteristics make it a morphologically distinctive species in the wild, especially because of its large-sized basidiomes.

#### Specimens examined.

China. Sichuan Province: Tibetan Autonomous Prefecture of Garzê, Batang County, Cuopugou, 20 July 2015, Mao-Qiang He, *ZRL2015429*; Zhubalong Natural Reserve, 29°39'39.48"N, 99°5'37.68"E, alt. 4311 m, 19 August 2020, by Xin-Yu Zhu, Xi-Xi Han, *ZRL20201190*, *ZRL20201375*; Litang County, Zhaga Mountain, 20 August 2020, Xi-Xi Han, Bin Cao, *ZRL20201260*, *ZRL20201261*, *ZRL20201278*; Nuoergai County, 13 September 1992, Xiaolan Mao, HMAS61642; Gansu Province: Zhangye, Sunan County, Kangle, 27 August 2018, Ming-Zhe Zhang, *ZRL20181933*; Beijing: Fangshan District, Baicaopan Nature Park, 1 August 2019, Rui-Lin Zhao, *ZRL20190162*; Xizang Autonomous Region: Nyingchi Municipality, Mêdog County, Xironggou, 29.42.33°N, 95.35.8°E, alt. 2800 m, 25 July 2021, Bin Cao, *ZRL20210938*, *ZRL20210939*; 28°36'26"N, 98°6'11"E, alt. 3848 m, *ZRL20231351*; Shigatse Municipality, Yadong County, 27.25.20°N, 88.55.6°E, alt. 3254, 27 July 2022, Mao-Qiang He, *ZRL20220286*; Jilin Province: 2 September 1992, Zongliu Chen, Suxiao Sun, HMAS59847; Xinjiang Uygur Autonomous Region: 10 August 1985, Li Fan, Yumei Li, HMAS86009; Qinghai Province: Beishan National Forestry Park, 36°55'41"N, 102°26'7"E, alt. 2427 m, 9 July 2023, Mao-Qiang He, *ZRL20230570*, *ZRL20235586*, *ZRL20235598*, *ZRL20235619*; 38°3'13"N, 100°23'54"E, alt. 3297 m, 29 August 2023, Mao-Qiang He, *ZRL20235824*.

### ﻿ Panaeolus subfoenisecii

Taxon classificationAnimaliaAgaricalesGaleropsidaceae

M.Q. He & R.L. Zhao sp. nov.

13D385C5-D634-5F47-95BE-7E95FE0CCBBD

860271

Fig. 13

#### Etymology.

sub refers to it being morphologically the same and phylogenetically close to *P. foenisecii*.

#### Diagnosis.

*Panaeolus subfoenisecii* is phylogenetically sister to *P. foenisecii* but has smooth basidiospores.

#### Holotype.

CHINA. Xizang Autonomous Region: Shigatse Municipality, Gyirong County, Gyirong Town, 28.22.39°N, 85.19.40°E, alt. 2780 m, 02 August 2022, Mao-Qiang He, HMAS287492 (*ZRL20220850*).

#### Description.

Pileus 10.6–15.5 mm in diam., obtusely conic, brown (pantone 2470 c), disc darker (pantone 2469 c), hygrophanous, sometimes paler at margin, surface dry, radially rugulose, margin straight or decurved. Lamellae adnate, subdistant, broad, mottled grayish to blackish, entire, edge white. Stipe equal, hollow, 43.0–52.1 mm long, 1.0–2.0 mm thick, brown, the same color as pileus (pantone 2469 c), smooth, longitudinally striate, pruinose on the side close to the cap, base with whitish mycelium.

Basidiospores 11.1–12.8 × 6.5–8.3 μm, [x = 12.2 ± 0.5 × 7.5 ± 0.1, Q = 1.4–1.8, Q_m_ = 1.6 ± 0.1, n = 20], ellipsoid, smooth, brown, blackish brown, thick wall with germ pore. Basidia 17.1–23.9 × 8.5–11.7 μm, 4-spored, smooth, hyaline. Cheilocystidia 14.4–43.6 × 3.1–7.7 μm, cylindro-clavate, with lightly inflated head and base, hyaline, smooth. Pleurocystidia absent. Cuticle composed of large vesicles, pileocystidia not observed. Caulocystidia absent.

**Figure 13.**
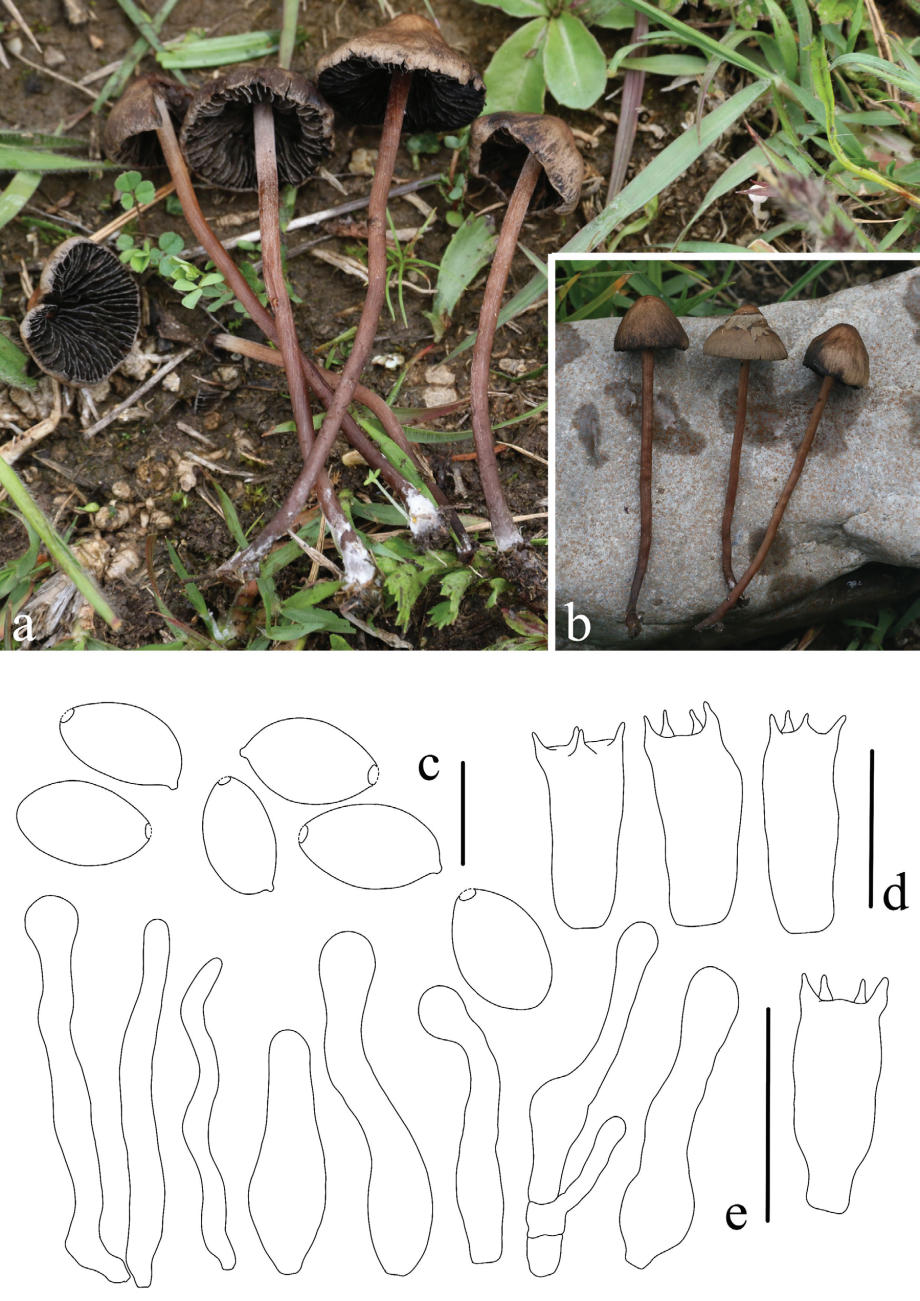
*Panaeolus subfoenisecii*. **a, b** basidiomes in the field; **c** basidiospores; **d** basidia; **e** cheilocystidia. Scale bars: 10 μm (**c**); 20 μm (**d**, **e**).

#### Habitat.

Grows on fertile soil as pastures and grasslands.

#### Psilocybin-producing.

Producing (*ZRL20220850*, Suppl. material 1: fig. S8).

#### Notes.

*Panaeolus subfoenisecii* is phylogenetically close to and macromorphologically resembles *P. foenisecii*. The two species can be separated under the microscope in that *P. subfoenisecii* has smooth basidiospores, whereas *P. foenisecii* has verrucose basidiospores.

#### Specimen examined.

China. Xizang Autonomous Region: Shigatse Municipality, Gyirong County, Gyirong Town, 28.22.39°N, 85.19.40°E, alt. 2780 m, 02^nd^ August 2022, Mao-Qiang He, *ZRL20220801*.

### ﻿ Panaeolus variabilicolor

Taxon classificationAnimaliaAgaricalesGaleropsidaceae

M.Q. He & R.L. Zhao sp. nov.

C91575F3-B4CE-5729-A4A7-CBED63705985

860272

Fig. 14

#### Etymology.

*variabilicolor* (Lat.) refers to several colors of basidiome are observed.

#### Diagnosis.

*Panaeolus variabilicolor* has hygrophanous or non-hygrophanous pileus, limoniform basidiospores, and cylindrical, clavate caulocystidia.

#### Holotype.

CHINA. Xizang Autonomous Region: Shigatse Municipality, Yadong County, 27.25.17°N, 88.56.32°E, alt. 3024 m, 26 July 2022, Mao-Qiang He, HMAS287496 (*ZRL20220096*).

#### Description.

Pileus 7.8–32.5 mm in diam., campanulate, conic, surface dry, hygrophanous, non-hygrophanous, color variable, gray (pantone 427c), grayish black (pantone 412c), light brown (pantone 166c), reddish brown (pantone 7583c), sometimes longitudinally striate near the margin, margin straight, uplifted, or decurved, occasionally with veil remnants. Lamellae adnate, close, entire, mottled grayish first then becoming blackish when mature, with a white edge. Stipe equal, hollow, 37.4–135.9 mm long, 1–2.7 mm thick, with some but darker color as pileus, smooth, slightly pruinose when basidiomes are fresh or young, base with whitish mycelium.

Basidiospores 9.9–12.2 × 7.5–8.5 μm, [x = 10.7 ± 0.6 × 8.0 ± 0.3, Q = 1.2–1.4, Q_m_ = 1.3 ± 0.1, n = 20], lager basidiospores were observed in *ZRL20220096* as 12.3–15.1 × 9.1–11.5 μm, [x = 14.1 ± 0.7 × 10.3 ± 0.7, Q = 1.3–1.5, Q_m_ = 1.4 ± 0.1, n = 20], limoniform, blackish brown when mature, smooth, thick wall, germ pore distinctive. Basidia 18.2–27.2 × 7.0–10.1 μm, 4-spored, smooth, hyaline. Cheilocystidia 15.4–34.8 × 3.8–8.2 μm, cylindrical with a slightly inflated apex and base, flexuose, hyaline, smooth. Pleurocystidia absent. Cuticle composed of large vesicles, pileocystidia not observed. Caulocystidia 12.9–53.8 × 4.2–7.8 μm, cylindrical, clavate.

**Figure 14.**
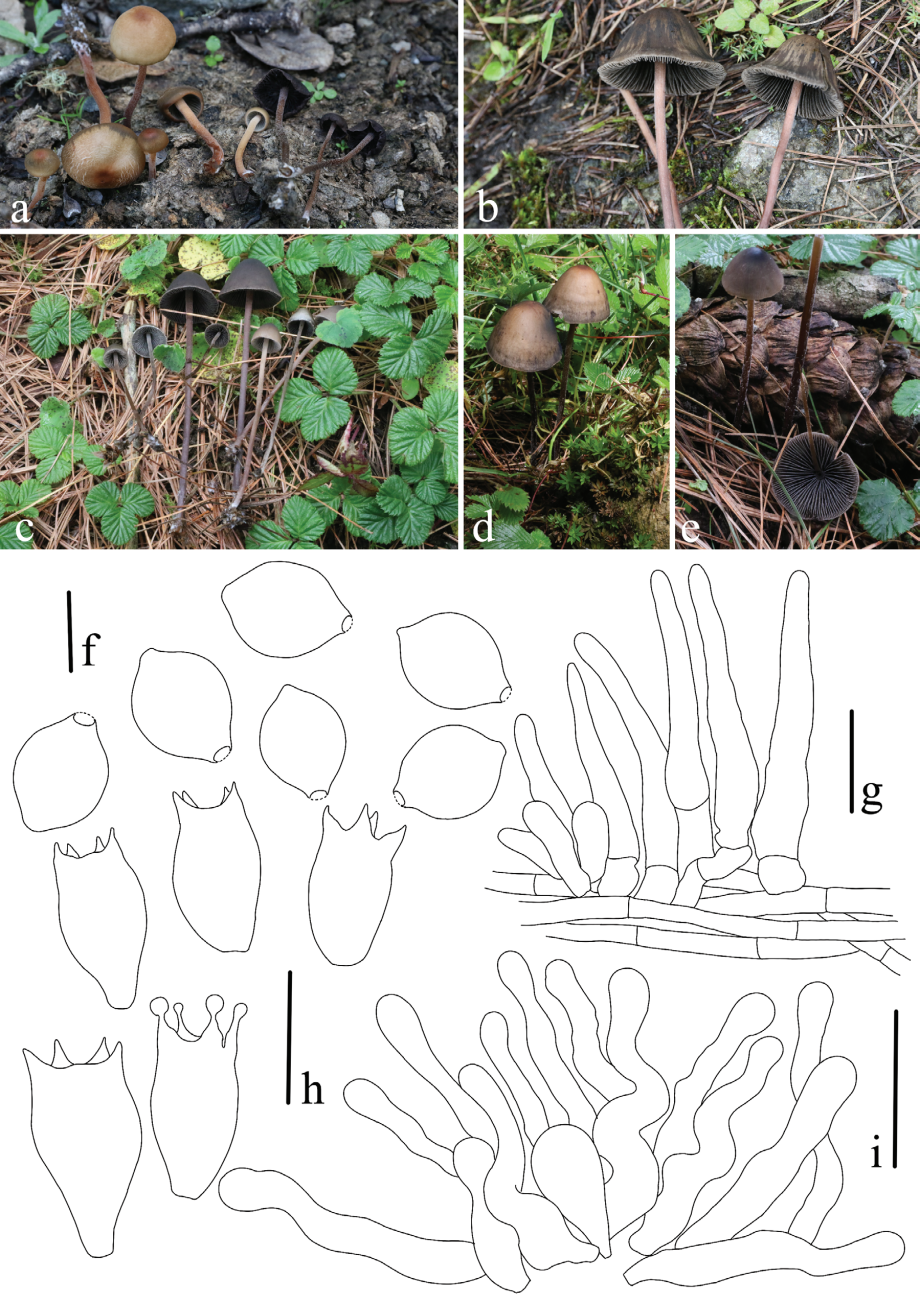
*Panaeolus variabilicolor*. **a–f** basidiomes in the field; **g** basidiospores; **h** caulocystidia; **i** basidia; **j** cheilocystidia. Scale bars: 10 μm (**g**); 20 μm (**h–j**).

#### Habitat.

Grows on fertile soil as pastures, grasslands, and forests, and on dungs.

#### Psilocybin-producing.

Nonproducing (*ZRL20220096*, see Suppl. material 1: fig. S9).

#### Notes.

Different basidiome colors were observed in *P. variabilicolor*. The variable basidiome color makes it difficult to distinguish this species in the field. For example, the basidiomes of ZRL20210525 are orange brown and were found on dung in grassland. However, the basidiomes of *ZRL20220075* and *ZRL2022096* are grayish black and were found on soil in forest. Thus far, this species has only been found in the Xizang Autonomous Region. In the phylogenetic tree (Fig. 3), *P. variabilicolor* has a sister relationship with the clade composed of *P. acuminatus* and *P. paludosus*. Compared with these two species, *P. variabilicolor* can be easily distinguished by its relatively large basidiomes (7.8–32.5 mm in diam.).

#### Specimens examined.

China. Xizang Autonomous Region: Nyingchi Municipality, Chawalong, 28.33.44°N, 98.15.27°E, alt. 2670 m, 21 July 2021, Mao-Qiang He, *ZRL20210525*; Shigatse Municipality, Yadong County, 27.25.17°N, 88.56.32°E, alt. 3024 m, 26 July 2022, Mao-Qiang He, *ZRL20220075*, *ZRL20220205*; Dinggyê County, 27.55.15°N, 87.21.37°E, alt. 3060 m, 29 July 2022, Dorji Phurbu, *ZRL20220144*; Gyirong County, Gyirong Twon, 28.23.45°N, 85.23.34°E, alt. 3441 m, 2 August 2022, Jia-Xin Li, *ZRL20220735*.

### ﻿ Panaeolus subg. Panaeolus

Taxon classificationAnimaliaAgaricalesGaleropsidaceae

(Fr.) Quél.

3D79680A-C856-5647-9760-B51F463C8718

#### Basionym.

Agaricus subgen. Panaeolus Fr., Summa vegetabilium Scandinaviae 2: 297 (1849)

#### Type species.

*Panaeolus papilionaceus*

#### Notes.

Traditionally, subgenus Panaeolus is roughly equivalent to *Panaeolus* s.s. According to the results of the phylogenetic analyses, the type species *P. papilionaceus* is clustered in clade B1 together with other species referred to as the *P. papilionaceus* species complex in some studies (Voto and Angelini 2024). Clade B is phylogenetically supported with statistical values of 97/96/1.0 (SH-aLRT/UFBoot/PP). Two subclades are supported: B1 is a widely distributed lineage with samples from the Americas, Asia, Europe, and Oceania, whereas B2, represented by a single species, *P. punjabensis* Asif, Firdous, Izhar, Niazi & Khalid, is known only from western Asia.

#### Species included.

*P. alcis* M.M. Moser, *P. desertorum* (Velen. & Dvořák) E.F. Malysheva, G. Moreno, Svetash. & M. Villarreal, *P. detriticola* Voto & Bougher, *P. pantropicalis* Voto, Angelini & Barrett, *P. papilionaceus*, *P. parvisporus* (Ew. Gerhardt) Voto & Angelini, *P. punjabensis*, *P. ranwuensis* M.Q. He, R.L. Zhao & B. Cao, *P. xiaolanii* M.Q. He & R.L. Zhao.

### ﻿ Panaeolus papilionaceus

Taxon classificationAnimaliaAgaricalesGaleropsidaceae

(Bull.) Quél., Mém. Soc. Émul. Montbéliard, Sér. 2 5: 152 [122 repr.] (1872)

B692FA70-6561-5607-B4F1-5CCB469990C1

Fig. 15

#### Basionym.

*Agaricus papilionaceus* Bull., Herb. Fr. (Paris) 1: 561 (1781).

#### Description.

Pileus 4–19 (–49) mm in diam., conical to broadly conical, surface dry, smooth, occasionally hygrophanous, color variable, from gray (pantone 427 c), grayish olive (pantone 7536 c) to black (pantone 412 c), brown (pantone 7587 c) is also observed (in *ZRL20220358*), usually paler at edge, margin entire, covered by white, triangular veil remnants. Lamellae adnate, close, broad, mottled grayish first then becoming blackish when mature, entire, with a white edge. Stipe equal, hollow, 13–140 mm long, up to 2 mm thick, always the same color as pileus, gray to black, dark purple, paler above, smooth, pruinose especially on the side close to the cap, occasionally with whitish mycelium.

Basidiospores 15.7–18.3 × 9.6–12.4 μm, [x = 16.9 ± 0.6 × 11.5 ± 0.7, Q = 1.4–1.7, Q_m_ = 1.3 ± 0.1, n = 20], limoniform, ellipsoid, blackish brown when mature, smooth, thick wall, protruding germ pore. Basidia 22.1–27.0 × 13.9–16.3 μm, 4-spored, smooth, hyaline. Cheilocystidia 23.2–33.4 × 2.9–6.6 μm, cylindrical or narrowly clavate, flexuose, hyaline, smooth. Pleurocystidia absent. Cuticle composed of large vesicles, pileocystidia spheropedunculate, hyaline, some light brown, darker at the base. Caulocystidia 43.0–60.9 × 4.1–7.7 μm, cylindrical or narrowly clavate, flexuose, hyaline and light brown, smooth.

**Figure 15.**
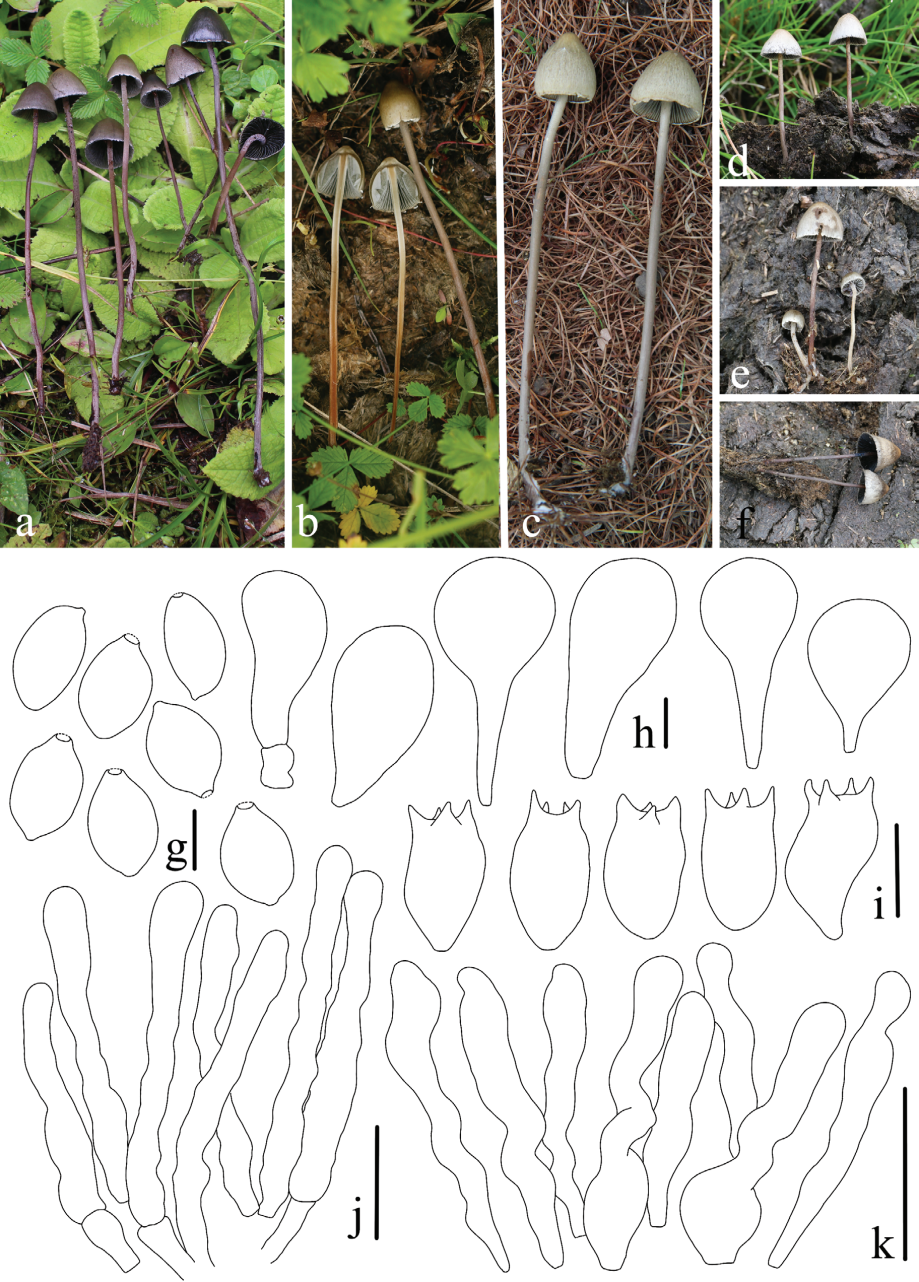
*Panaeolus papilionaceus*. **a–f** basidiomes in the field; **g** basidiospores; **h** pileocystidia; **i** basidia; **j** caulocystidia; **k** cheilocystidia. Scale bars: 10 μm (**g**, **h**); 20 μm (**i**, **j**, **k**).

#### Habitat.

Grows in pastures, on dung of horses and cows.

#### Psilocybin-producing.

Nonproducing (*ZRL20210652*, see Suppl. material 1: fig. S4).

#### Notes.

*Panaeolus papilionaceus* is characterized by its small-sized basidiomes, a conical to broadly conical pileus, and limoniform basidiospores. An extremely small mature basidiome was observed in ZRL20220202, in which the pileus is 4 mm in diam. and the stipe is 13 mm long.

#### Specimens examined.

China. Yunan Province: Dêqên Tibetan Autonomous Prefecture, Shangri-la City, 23 July 2012, collected by Rui-Lin Zhao, *ZRL2012412*; Inner Mongolia Autonomous Region: Hulunbeier City, Lalantun, Chaihe Wildlife Natural Reserve, 47°32'44"N, 121°13'22"E, alt. 810 m, 27 August 2017, Zhilin Ling, *ZRL20170629*; Xinbaerhuzuo County, 49°45'20"N, 120°12'9"E, alt. 510 m, 26 August 2017, Zhilin Ling, *ZRL20170653*; Gansu Province: Wuwei, Tianzhu County, 19 August 2018, Bin Cao, *ZRL20181046*; Beijing: Fangshan District, Baicaopan Nature Park, 1 August 2019, Rui-Lin Zhao, *ZRL20190135*; Sichuan Province: Garze Tibetan Autonomous Prefecture, Litang County, 21 August 2019, Bin Cao, *ZRL20191490*; Sichuan Province: Li County, 1 August 1958, Qionglin Hu, HMAS23784; Jiuzhaigou, 9 June 1983, Huaan Wen, Jingjun Su, HMAS51233; Xizang Autonomous Region: Nyingchi Municipality, Chawalong, 28.36.46°N, 98.5.22°E, alt. 4110 m, 21 July 2021, Mao-Qiang He, *ZRL20210652*; Qamdo Municipality, Baxoi County, 29.47.37°N, 95.53.20°E, alt. 2870 m, 23 July 2021, Rui-Lin Zhao, *ZRL20210692*, *ZRL20210750*; Xizang Autonomous Region: Shigatse Municipality, Yadong County, 27.25.17°N, 88.56.32°E, alt. 3024 m, 26 July 2022, Mao-Qiang He, *ZRL20220092*, *ZRL20220153*, *ZRL20220201*, *ZRL20220202*, *ZRL20220203*, *ZRL20220358*; Gyirong County, 28.22.39°N, 85.19.40°E, alt. 2780 m, 2^nd^ August 2022, Jiaxin Li, *ZRL20220703*; Ningxia Hui Autonomous Region, 24 August 1997, Huaan Wen, Suxiao Sun, HMAS72699; Hubei Province: Shennongjia forestry district, Dajiuhu, 24 June 2023, Mao-Qiang He, *ZRL20230343*.

### ﻿ Panaeolus parvisporus

Taxon classificationAnimaliaAgaricalesGaleropsidaceae

(Ew. Gerhardt) Voto & Angelini, Mycological Observations 9: 21 (2024)

C6C22B80-5A18-5499-8D35-CCF489051C94

Fig. 16

#### Description.

Pileus 16–38 mm in diam., ovoid when young, then parabolic or occasionally convex when getting mature, light brown (pantone 4685 c), brown (pantone 4645 c), surface dry, surface could be cracked with erect darker scales, margin slightly exceeding gills. Lamellae adnate, close, broad, mottled grayish first then becoming blackish-brown when mature, entire, with a white edge. Stipe equal, hollow, 19–54 mm long, 2–4 mm thick, brown, usually con-color with the pileus, smooth, pruinose, base with whitish mycelium, base getting darker when touched or bruised.

Basidiospores 14.4–16.9 × 9.6–11.1 μm, [x = 15.7 ± 0.6 × 10.4 ± 0.5, Q = 1.4–1.6, Q_m_ = 1.5 ± 0.1, n = 20], ellipsoid, elongate, blackish brown when mature, smooth, thick wall, germ pore distinctive. Basidia 26.4–33.8 × 9.6–12.1 μm, 4-spored, smooth, hyaline. Cheilocystidia 18.6–30.4 × 3.5–8.2 μm, cylindrical or narrowly clavate, flexuose, occasionally with an inflated apex, hyaline and yellowish-brown, smooth. Pleurocystidia absent. Cuticle composed of large vesicles, pileocystidia 23.3–46.5 × 11.5–28.1 μm, spheropedunculate, hyaline, occasionally yellowish-brown. Caulocystidia 17.7–60.2 × 3.4–7.4 μm, cylindrical or narrowly clavate, flexuose, hyaline, occasionally light brown, smooth.

**Figure 16.**
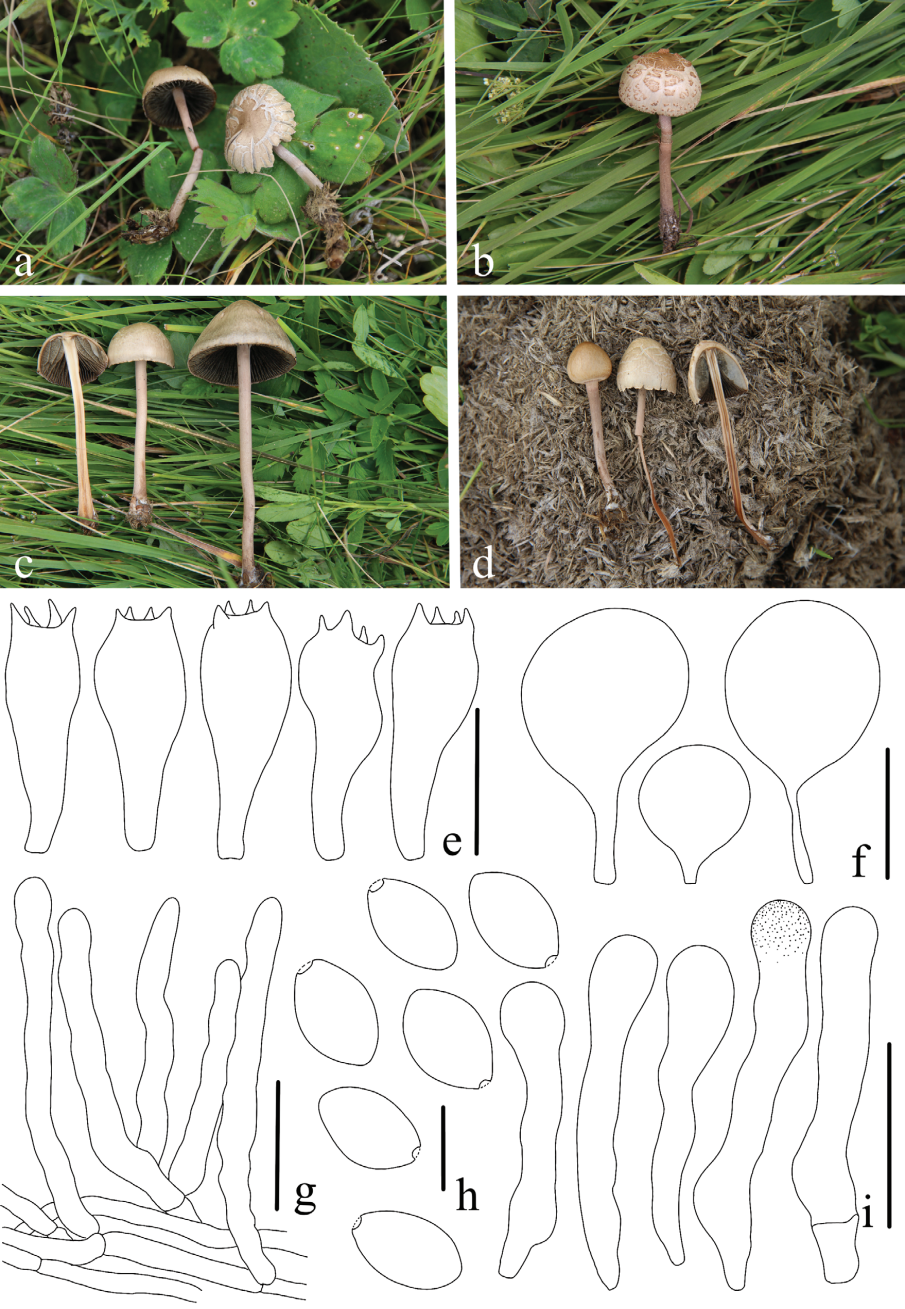
*Panaeolus parvisporus*. **a–d** basidiomes in the field; **e** basidia; **f** pileocystidia; **g** caulocystidia; **h** basidiospores; **i** cheilocystidia. Scale bars: 10 μm (**h**); 20 μm (**e**, **g**, **i**).

#### Habitat.

Grows on fertile soil as pastures, grasslands, and forests.

#### Psilocybin-producing.

Nonproducing (*ZRL20170604*, see Suppl. material 1: fig. S3).

#### Notes.

*Panaeolus parvisporus* is a recently proposed species based on P. papilionaceus var. parvisporus from the *P. papilionaceus* complex (Voto and Angelini 2024). Five samples from China share identical ITS sequences with two European samples of *P. parvisporus*. However, they differ in basidiospores, with our samples having larger basidiospores.

#### Specimens examined.

China. Inner Mongolia Autonomous Region: Hulunbeier, Xinbaerhuzuo County, 49°45'20"N, 120°12'9"E, alt. 510 m, 26 August 2017, Zhilin Ling, *ZRL20170602*, *ZRL20170603*, *ZRL20170604*, *ZRL20170654*; Inner Mongolia Autonomous Region, Chaihe Wildlife Nature Reserve, Zhalantun City, 25 August 2017, Zhilin Ling, *ZRL20170634*; Ningxia Hui Autonomous Region: 11 September 1995, Xiaolan Mao, Cangkuan Wang, HMAS69762.

### ﻿ Panaeolus xiaolanii

Taxon classificationAnimaliaAgaricalesGaleropsidaceae

M.Q. He & R.L. Zhao sp. nov.

B9A82FC5-C57B-512A-B722-D0C2E2039F4B

852930

Fig. 17

#### Etymology.

*xiaolanii* is in honor of the Chinese mycologist Xiao-Lan Mao, who made a great contribution to the macrofungal research in China.

#### Diagnosis.

*Panaeolus xiaolanii* has small basidiomes with gray, hygrophanous pileus, limoniform basidiospores, and cylindrical, flexuose cheilocystidia.

#### Holotype.

CHINA. Xizang Autonomous Region: Shigatse Municipality, Yadong County, Xiayadong Village, Boluoka grassland, 27.22.8°N, 88.58.25°E, alt. 2872 m, 22 July 2022, Mao-Qiang He, HMAS287493 (*ZRL20220031*).

#### Description.

Pileus 11–38 mm in diam., conical to broadly conical, surface dry, smooth, hygrophanous, color variable, from gray (pantone 427 c), grayish yellow (pantone 7536 c) to grayish black (pantone 412 c), usually paler at edge, some with crenulate margin, margin usually covered by white veil remnants. Lamellae adnate, close, broad, mottled grayish first then becoming blackish when mature, entire, with a white edge. Stipe equal, hollow, 34–130 mm long, 1–3 mm thick, always the same color as pileus, gray to black, dark purple, paler above, smooth, pruinose especially on the side close to the cap, base getting darker when touched or bruised and occasionally with whitish mycelium.

**Figure 17.**
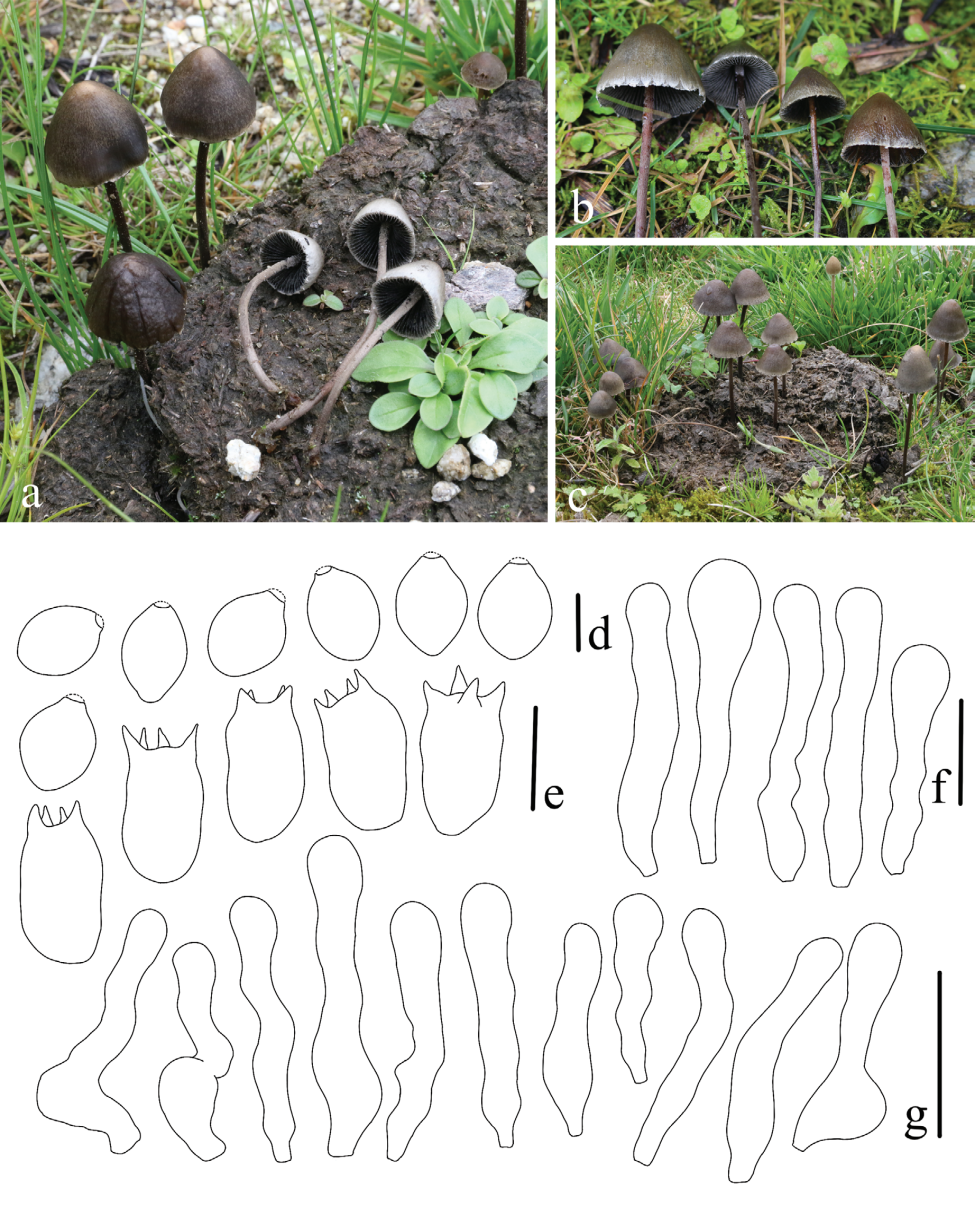
*Panaeolus xiaolanii*. **a–c** basidiomes in the field; **d** basidiospores; **e** basidia; **f** cheilocystidia; **g** caulocystidia. Scale bars: 10 μm (**d**); 20 μm (**e**, **f**, **g**).

Basidiospores 13.8–16.3 × 10.7–12.0 μm, [x = 15.3 ± 0.7 × 11.4 ± 0.3, Q = 1.2–1.5, Q_m_ = 1.3 ± 0.1, n = 20], limoniform, blackish brown when mature, smooth, thick wall, protruding germ pore. Basidia 19.1–26.9 × 12.9–15.8 μm, 4-spored, smooth, hyaline. Cheilocystidia 23.3–32.4 × 3.9–7.4 μm, cylindrical or narrowly clavate, flexuose, hyaline, smooth. Pleurocystidia absent. Cuticle composed of large vesicles, pileocystidia not observed. Caulocystidia 31.4–55.7 × 5.5–9.6 μm, cylindrical or narrowly clavate, flexuose, hyaline and light brown, smooth.

#### Habitat.

Grows in pastures, on dung of horses and cows.

#### Psilocybin-producing.

Nonproducing (*ZRL20220031*, see Suppl. material 1: fig. S5).

#### Notes.

Compared with other phylogenetically closely related species, *P. xiaolanii* can be distinguished from *P. papilionaceus* in the field by its more conical and darker pileus. Additionally, the two species differ in basidiospores, with *P. papilionaceus* having ellipsoid basidiospores, whereas *P. xiaolanii* has limoniform basidiospores. It is difficult to separate *P. xiaolanii* from *P. parvisporus* in the field. A relatively distinguishable difference is the larger basidiospores of *P. xiaolanii*.

#### Other specimen examined.

China. Jilin Province: Yanbian Korean Autonomous Prefecture, Longjin City, Xianfeng National Forestry Park, 23 July 2016, *ZRL20160575*; Xizang Autonomous Region: Shigatse Municipality, Yadong County, Xiayadong village, Boluoka grassland, 27.22.8°N, 88.58.25°E, alt. 2872 m, 22 July 2022, collected by Mao-Qiang He, *ZRL20220039*, *ZRL20220044*, *ZRL20220154*, *ZRL20220155*; Renqinggang, 27.25.20°N, 88.55.6°E, alt. 3254, 23 July 2022, Mao-Qiang He, *ZRL20220360*, *ZRL20220381*; Dinggyê County, Chentang Town, 27.52.17°N, 87.25.18°E, alt. 2600 m, 30 July 2022, Mao-Qiang He, *ZRL20220451*, *ZRL20220452*; Gyirong County, Jilonggou, 28.24.35°N, 85.18.54°E, alt. 2935 m, 1^st^ August 2022, Rui-Lin Zhao, *ZRL20220560*; Chongse, 28.22.39°N, 85.19.40°E, alt. 2780 m, 2^nd^ August 2022, Bin Cao, *ZRL20220697*.

## ﻿Discussion

### ﻿Taxonomic system and species diversity of *Galeropsidaceae*

In this study, based on phylogenomic and multigene phylogenetic analyses, divergence time estimation, and morphological characteristics, the panaeo-clade is further demonstrated to be a distinct family separate from *Bolbitiaceae*, as assumed in previous studies (Kalichman et al. 2020). Additional evidence from this study includes the following: first, multigene phylogenetic analyses with expanded taxon sampling confirm the monophyly of the panaeo-clade and its sister relationship to *Bolbitiaceae* within *Agaricineae*; second, the divergence time of the panaeo-clade (87 Myr) closely aligns with that of other families in *Agaricineae* (59–130 Myr); third, the combination of morphological characteristics, including a black spore print and variegated lamellae, distinguishes the panaeo-clade from *Bolbitiaceae* and other families in *Agaricineae*. The type species of *Galeropsidaceae*, *Galeropsis desertorum* Velen. & Dvořák, is found to be a member of the panaeo-clade (Malysheva et al. 2019). Consequently, this clade should be named *Galeropsidaceae*, which has nomenclatural priority (Kalichman et al. 2020).

Five main clades are revealed within *Galeropsidaceae* (clades A–E; see Fig. 3). Four main clades are found within *Panaeolus*, three of which correspond to the three subgenera, namely clade A (subg. Panaeolina), clade B (subg. Panaeolus), and clade C (subg. Bresadolomyces). Clade D is represented by a single specimen from the Dominican Republic (ANGE1393: *P. sylvaticus*), which does not cluster with any of the other clades in *Panaeolus*. Clade E represents *Staktophyllus*, a genus separated from *Panaeolus*, currently represented only by *S. guttulatus*. *Panaeolopsis* has previously been considered a genus within *Galeropsidaceae* (Kalichman et al. 2020). In this study, *Panaeolopsis nirimbii* (PERTH7680368) is clustered in subclade A2 within subg. Panaeolina (see Fig. 3), which agrees with the proposal to synonymize *Panaeolopsis* under *Panaeolus* (Angelini and Voto 2023). *Crucispora* was established to accommodate species with *Panaeolus*-like basidiomes but distinctive cruciform-rhomboid basidiospores. *Panaeolina rhombisperma* was therefore transferred to *Crucispora* based on its cruciform-rhomboid basidiospores (Horak 1980). According to the phylogenetic results, *C. rhombisperma* is grouped together with members of subg. Bresadolomyces and is thus proposed as a synonym of *Panaeolus*.

The circumscription of subgenera in *Panaeolus* is mainly based on phylogenetic evidence. There is no known single morphological synapomorphy that unites the subgenera of *Panaeolus*. For example, basidiospore morphology (shape and ornamentation) has long been treated as a diagnostic character between genera in the taxonomic system of *Panaeolus* (Singer 1986; Gerhardt 1996). Accordingly, *Panaeolina* was proposed to include species with verrucose basidiospores, and *Crucispora* was proposed for species with cruciform-rhomboid basidiospores. However, at present, only *P. foenisecii* has been observed to possess verrucose basidiospores and is nested in subclade A2, with a sister relationship to *P. subfoenisecii*, which has smooth basidiospores. Conversely, species with cruciform-rhomboid basidiospores are nested within subg. Bresadolomyces. Most species of *Panaeolus* have smooth basidiospores. Furthermore, the Anellaria-like morphotype, characterized by robust, pale-colored basidiomes with glabrous pilei, occurs in phylogenetically distinct lineages, such as *P. semiovatus* and *P. antillarum* in subclade A3 and *P. medogensis* in subclade A1.

*Galeropsidaceae* species are characterized by small- to medium-sized basidiomes, a black spore print, and variegated lamellae, with many coprophilous species. These morphological characters generally distinguish them from other families in *Agaricineae*. However, field identification at the species level remains challenging because of high morphological plasticity. For example, six samples of *P. variabilicolor* collected from the Xizang Autonomous Region exhibited four different pileus morphotypes: reddish brown and convex (Fig. 12a), grayish brown and campanulate (Fig. 12b), grayish brown to black and conic (Fig. 12c, e), and reddish brown and conic (Fig. 12d). These variations in pileus morphology may be related to different habitats. ITS is widely used as a DNA barcode for *Galeropsidaceae*; however, in this study, ITS was found to be unsuitable for reliable species identification within *Galeropsidaceae*. In particular, for subg. Panaeolus, ITS shows much lower polymorphism than commonly used protein-coding genes (*rpb*1, *rpb*2, and *tef*1). For example, *P. xiaolanii* and *P. parvisporus* share identical ITS sequences but differ by more than 10 nucleotide positions in *tef*1.

Based on records from previous studies and the new species identified in this study from China, there are currently 88 accepted species of *Galeropsidaceae* worldwide (Voto and Angelini 2022; Asif et al. 2023; Strauss et al. 2023). Over the past 10 years, six new species have been described worldwide, but only three of these were introduced with molecular data. Our phylogenetic analyses revealed many unnamed samples occupying distinct positions within *Galeropsidaceae*, particularly in subclades B1 and C1. This suggests that species diversity in *Galeropsidaceae* remains underexplored and that further sampling combined with molecular phylogenetic analyses is needed. It can be speculated that more than 100 species of *Galeropsidaceae* may exist worldwide.

### ﻿The evolution of coprophilous and psilocybin-producing traits of *Galeropsidaceae*

*Galeropsidaceae* diverged approximately 87 Myr during the Cretaceous period, which aligns with the divergence times of most families in *Agaricales* (59–130 Myr; see Fig. 1). Both genera within *Galeropsidaceae* originated during the Cretaceous. *Staktophyllus* occupies the basal position within *Galeropsidaceae*, suggesting that *Galeropsidaceae* may have originated from a non-coprophilous ancestor, as *S. guttulatus* is mainly found on sandy soil (Bresadola 1883; Seidmohammadi et al. 2019). It can therefore be inferred that the coprophilous lifestyle is not homologous across the subgenera of *Panaeolus*. Each subgenus appears to have followed an independent evolutionary trajectory with respect to substrate preference. Ruminants and horses are thought to have played key roles in the evolution of coprophilous fungi (Halbwachs and Bässler 2020; Zhu and Bau 2024). The diversification of ruminants and horses occurred during a period similar to that of speciation in most *Galeropsidaceae* species. For example, most subfamilies of ruminants and genera of horses diverged during the Miocene, which coincides with the divergence of many *Galeropsidaceae* species (MacFadden 2005; Chen et al. 2019; Fig. 4 in this study). Additionally, at least two gasteromycetation events were inferred within *Galeropsidaceae*. One event occurred at least 2.6 Myr ago in subg. Panaeolina, represented by *P. plantaginiformis*. Another event occurred at least 7.4 Myr ago in subg. Panaeolus, represented by *P. desertorum*.

Two species, *P. subfoenisecii* and *P. cinctulus*, were confirmed to produce psilocybin in this study. Another species, *P. cyanescens*, is well known for psilocybin production, but psilocybin was not detected in this study, possibly due to the age of the samples examined (HMAS63187, collected 29 years ago), although psilocybin has been detected in 50-year-old *Psilocybe* samples (Bradshaw et al. 2022). Previous genomic and chemical studies have confirmed the psilocybin-producing properties of *P. cyanescens* (Stijve 1992; Reynolds et al. 2018). These three species are distributed across two lineages within *Galeropsidaceae*, namely subclades A2 and C1, both of which diverged around 30 Myr ago. Notably, within subclade A2, *P. subfoenisecii* and *P. foenisecii* are sister species, yet *P. foenisecii* lacks the psilocybin-producing trait. Previous studies have suggested that horizontal gene transfer between genera is an important mechanism driving the diversity of hallucinogenic mushrooms in *Agaricales* (Reynolds et al. 2018; Bradshaw et al. 2023). Whether horizontal gene transfer occurred between these sister species remains an intriguing question for future study.

## Supplementary Material

XML Treatment for Galeropsidaceae

XML Treatment for Panaeolus

XML Treatment for Panaeolus subg. Bresadolomyces

XML Treatment for Panaeolus cyanescens

XML Treatment for Panaeolus subg. Panaeolina

XML Treatment for Panaeolus antillarum

XML Treatment for Panaeolus cinctulus

XML Treatment for Panaeolus foenisecii

XML Treatment for Panaeolus grandis

XML Treatment for Panaeolus limoniformisporus

XML Treatment for Panaeolus medogensis

XML Treatment for Panaeolus nigrescens

XML Treatment for Panaeolus pallidus

XML Treatment for Panaeolus semiovatus

XML Treatment for Panaeolus subfoenisecii

XML Treatment for Panaeolus variabilicolor

XML Treatment for Panaeolus subg. Panaeolus

XML Treatment for Panaeolus papilionaceus

XML Treatment for Panaeolus parvisporus

XML Treatment for Panaeolus xiaolanii

## ﻿Additional information

### Use of AI

No use of AI was reported.

### Adherence to national and international regulations

All the fungal strains used in this study have been legally obtained, respecting the Convention on Biological Diversity (Rio Convention).

### Author ORCIDs

Mao-Qiang He https://orcid.org/0000-0002-9300-7484

Fei Liu https://orcid.org/0000-0003-1175-4070

Jia-Xin Li https://orcid.org/0000-0002-6434-3729

Rui-Lin Zhao https://orcid.org/0000-0001-8129-9339

### Data availability

All of the data that support the findings of this study are available in the main text or Supplementary Information.

## Supplementary materials

Supplementary material 1 HPLC-MS chromatograms Data type zip https://binary.pensoft.net/file/1515057This dataset is made available under the Open Database License (http://opendatacommons.org/licenses/odbl/1.0/). The Open Database License (ODbL) is a license agreement intended to allow users to freely share, modify, and use this Dataset while maintaining this same freedom for others, provided that the original source and author(s) are credited.Mao-Qiang He, Wen-Qiang Yang, Dorji Phurbu, Fei Liu, Jia-Xin Li, Bin Cao, Rui-Lin Zhao

Supplementary material 2 *Agaricineae* sixgene Data type phy https://binary.pensoft.net/file/1515058This dataset is made available under the Open Database License (http://opendatacommons.org/licenses/odbl/1.0/). The Open Database License (ODbL) is a license agreement intended to allow users to freely share, modify, and use this Dataset while maintaining this same freedom for others, provided that the original source and author(s) are credited.Mao-Qiang He, Wen-Qiang Yang, Dorji Phurbu, Fei Liu, Jia-Xin Li, Bin Cao, Rui-Lin Zhao

Supplementary material 3 *Galeropsidaceae* ITS Data type fas https://binary.pensoft.net/file/1515059This dataset is made available under the Open Database License (http://opendatacommons.org/licenses/odbl/1.0/). The Open Database License (ODbL) is a license agreement intended to allow users to freely share, modify, and use this Dataset while maintaining this same freedom for others, provided that the original source and author(s) are credited.Mao-Qiang He, Wen-Qiang Yang, Dorji Phurbu, Fei Liu, Jia-Xin Li, Bin Cao, Rui-Lin Zhao

Supplementary material 4 *Galeropsidaceae* LSU Data type fas https://binary.pensoft.net/file/1515060This dataset is made available under the Open Database License (http://opendatacommons.org/licenses/odbl/1.0/). The Open Database License (ODbL) is a license agreement intended to allow users to freely share, modify, and use this Dataset while maintaining this same freedom for others, provided that the original source and author(s) are credited.Mao-Qiang He, Wen-Qiang Yang, Dorji Phurbu, Fei Liu, Jia-Xin Li, Bin Cao, Rui-Lin Zhao

Supplementary material 5 *Galeropsidaceae* rpb1 Data type fas https://binary.pensoft.net/file/1515061This dataset is made available under the Open Database License (http://opendatacommons.org/licenses/odbl/1.0/). The Open Database License (ODbL) is a license agreement intended to allow users to freely share, modify, and use this Dataset while maintaining this same freedom for others, provided that the original source and author(s) are credited.Mao-Qiang He, Wen-Qiang Yang, Dorji Phurbu, Fei Liu, Jia-Xin Li, Bin Cao, Rui-Lin Zhao

Supplementary material 6 *Galeropsidaceae* rpb2 Data type fas https://binary.pensoft.net/file/1515062This dataset is made available under the Open Database License (http://opendatacommons.org/licenses/odbl/1.0/). The Open Database License (ODbL) is a license agreement intended to allow users to freely share, modify, and use this Dataset while maintaining this same freedom for others, provided that the original source and author(s) are credited.Mao-Qiang He, Wen-Qiang Yang, Dorji Phurbu, Fei Liu, Jia-Xin Li, Bin Cao, Rui-Lin Zhao

Supplementary material 7 *Galeropsidaceae* SSU Data type fas https://binary.pensoft.net/file/1515063This dataset is made available under the Open Database License (http://opendatacommons.org/licenses/odbl/1.0/). The Open Database License (ODbL) is a license agreement intended to allow users to freely share, modify, and use this Dataset while maintaining this same freedom for others, provided that the original source and author(s) are credited.Mao-Qiang He, Wen-Qiang Yang, Dorji Phurbu, Fei Liu, Jia-Xin Li, Bin Cao, Rui-Lin Zhao

Supplementary material 8 *Galeropsidaceae* tef1 Data type fas https://binary.pensoft.net/file/1515064This dataset is made available under the Open Database License (http://opendatacommons.org/licenses/odbl/1.0/). The Open Database License (ODbL) is a license agreement intended to allow users to freely share, modify, and use this Dataset while maintaining this same freedom for others, provided that the original source and author(s) are credited.Mao-Qiang He, Wen-Qiang Yang, Dorji Phurbu, Fei Liu, Jia-Xin Li, Bin Cao, Rui-Lin Zhao

Supplementary material 9 *Agaricineae* genomic data Data type zip https://binary.pensoft.net/file/1515065This dataset is made available under the Open Database License (http://opendatacommons.org/licenses/odbl/1.0/). The Open Database License (ODbL) is a license agreement intended to allow users to freely share, modify, and use this Dataset while maintaining this same freedom for others, provided that the original source and author(s) are credited.Mao-Qiang He, Wen-Qiang Yang, Dorji Phurbu, Fei Liu, Jia-Xin Li, Bin Cao, Rui-Lin Zhao
