# Supplementary figures and images for: ﻿Systematic study of Panaeolus (Agaricales, Galeropsidaceae) sensu lato and psilocybin-producing traits of species from China
Source: IMA Fungus. 2026 Jan 19;17:e167329. doi: 10.3897/imafungus.17.167329 (PMC12835877; doi:10.3897/imafungus.17.167329)

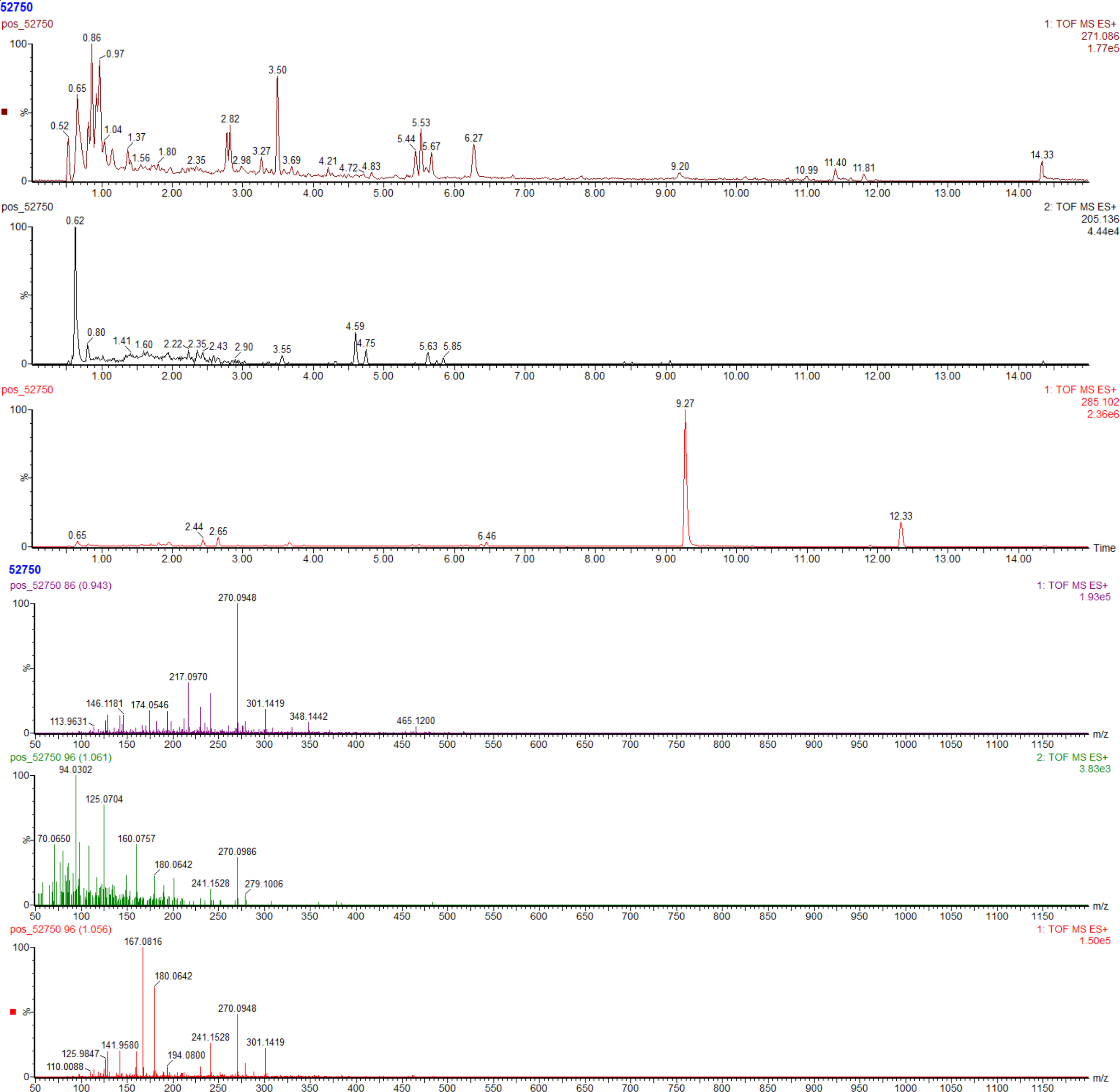

Supplement: Supplementary material 1 — HPLC-MS chromatograms [file imafungus-17-e167329-s001.zip › Supplementary Fig. S1 HMAS52750.png]

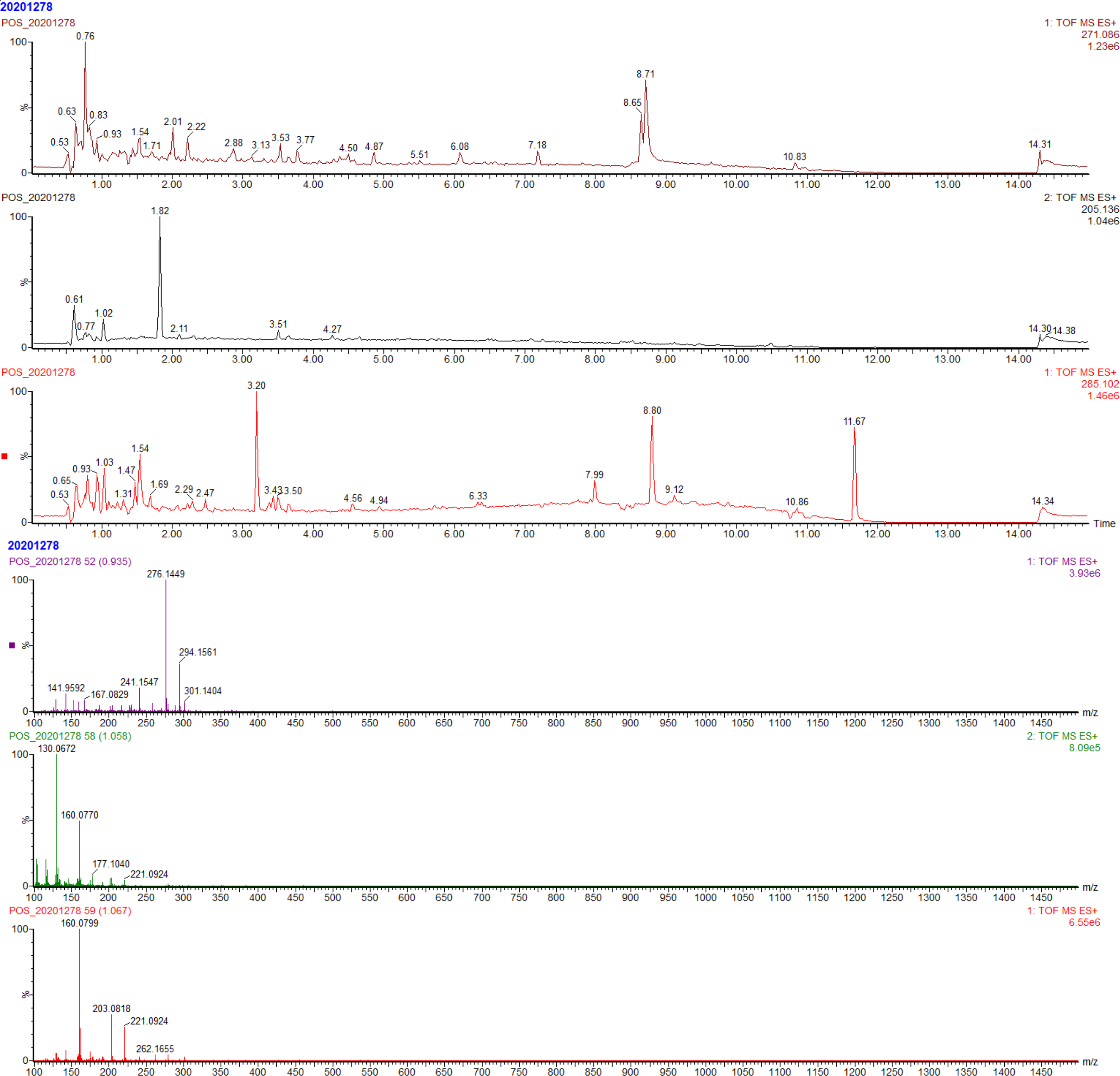

Supplement: Supplementary material 1 — HPLC-MS chromatograms [file imafungus-17-e167329-s001.zip › Supplementary Fig. S2 ZRL20201278.png]

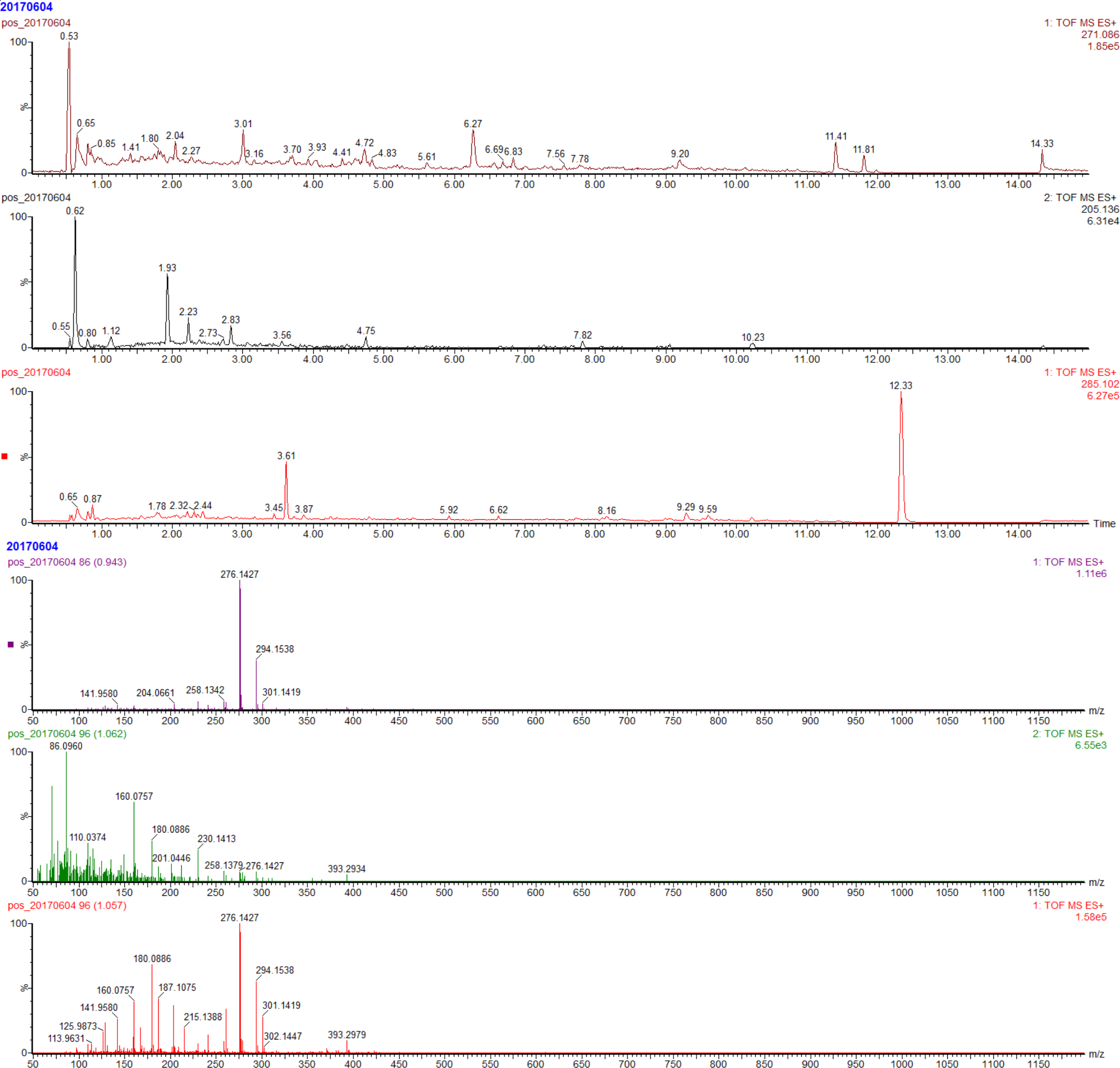

Supplement: Supplementary material 1 — HPLC-MS chromatograms [file imafungus-17-e167329-s001.zip › Supplementary Fig. S3 ZRL20170604.png]

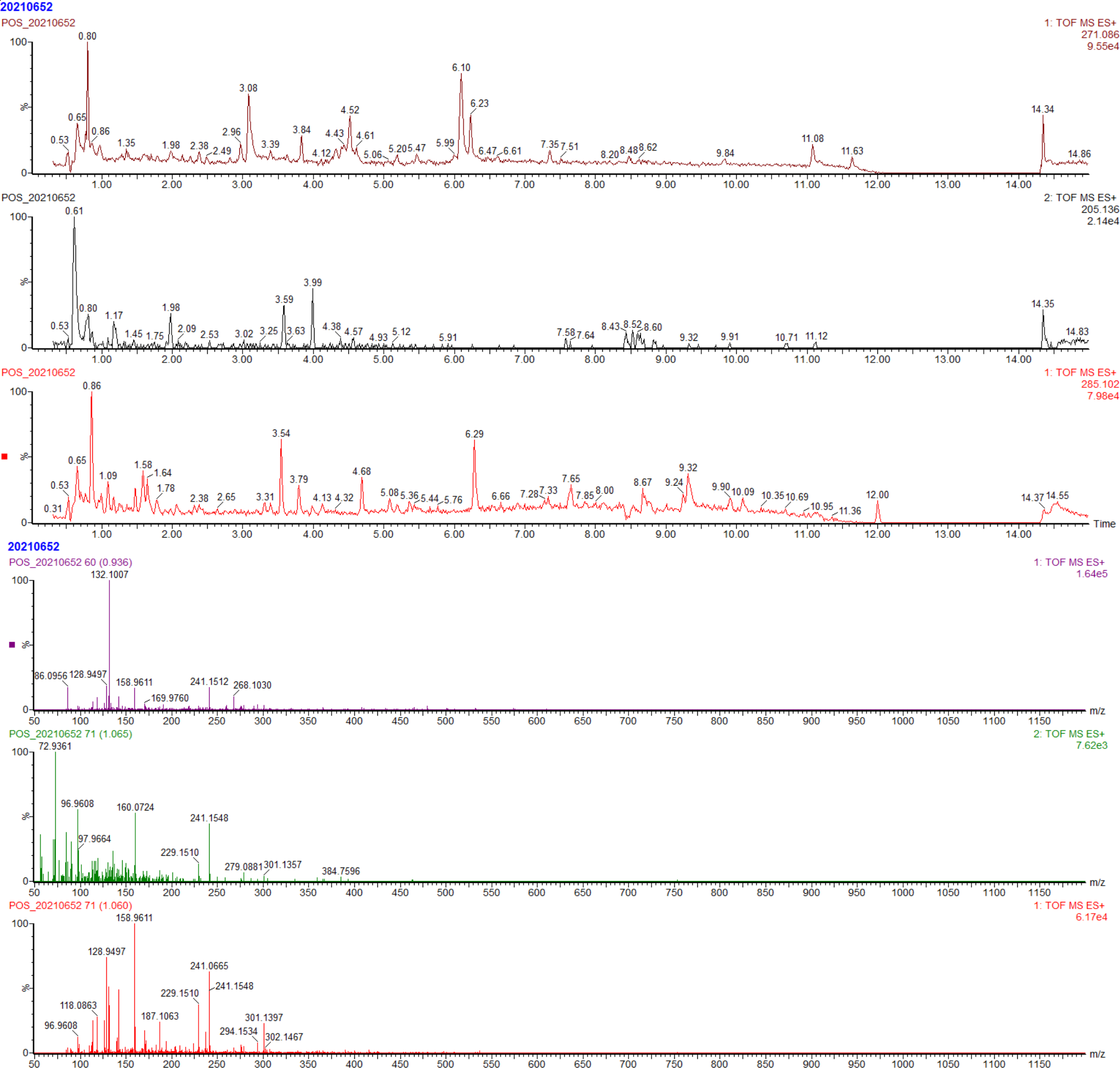

Supplement: Supplementary material 1 — HPLC-MS chromatograms [file imafungus-17-e167329-s001.zip › Supplementary Fig. S4 ZRL20210652.png]

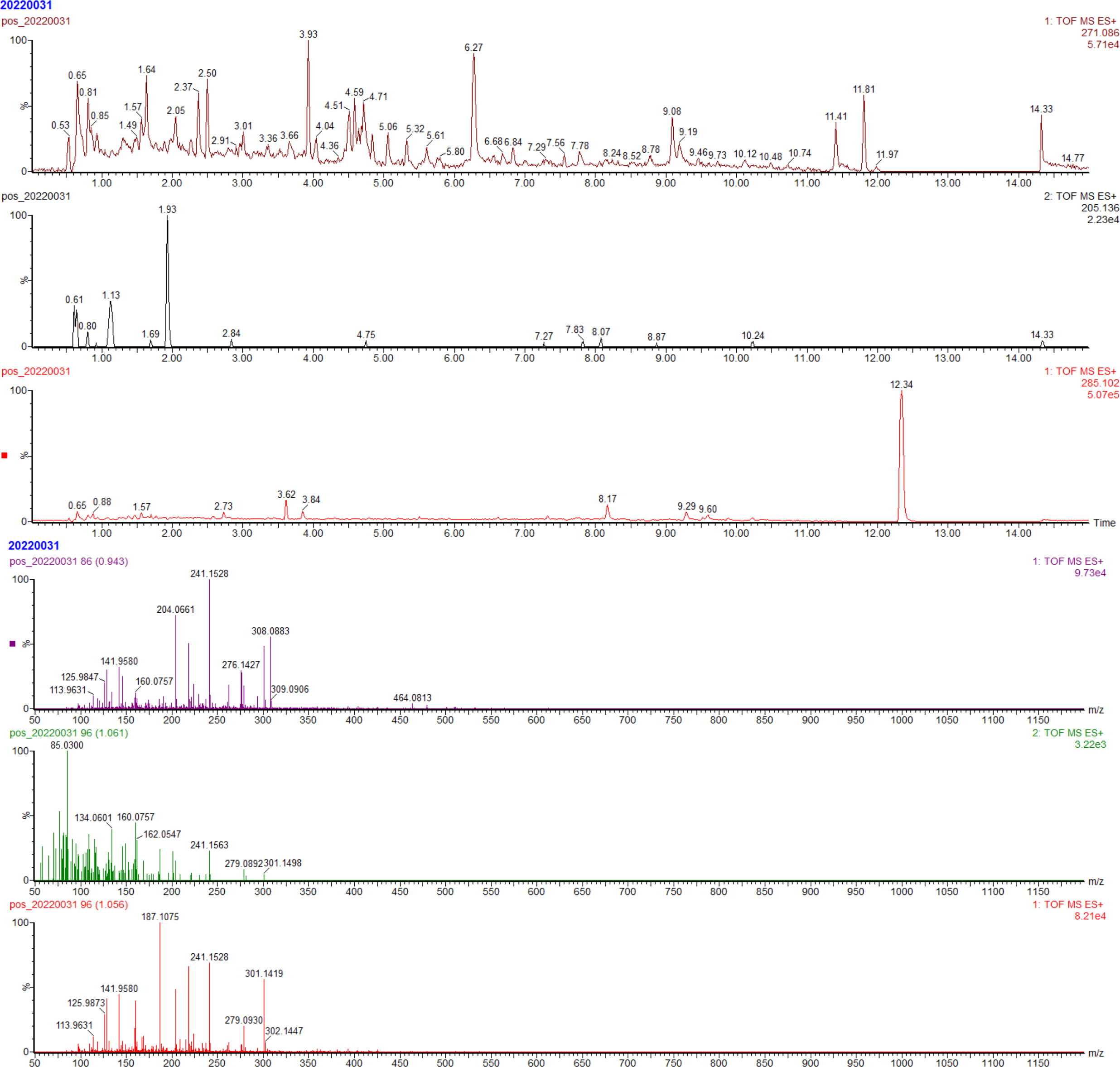

Supplement: Supplementary material 1 — HPLC-MS chromatograms [file imafungus-17-e167329-s001.zip › Supplementary Fig. S5 ZRL20220031.png]

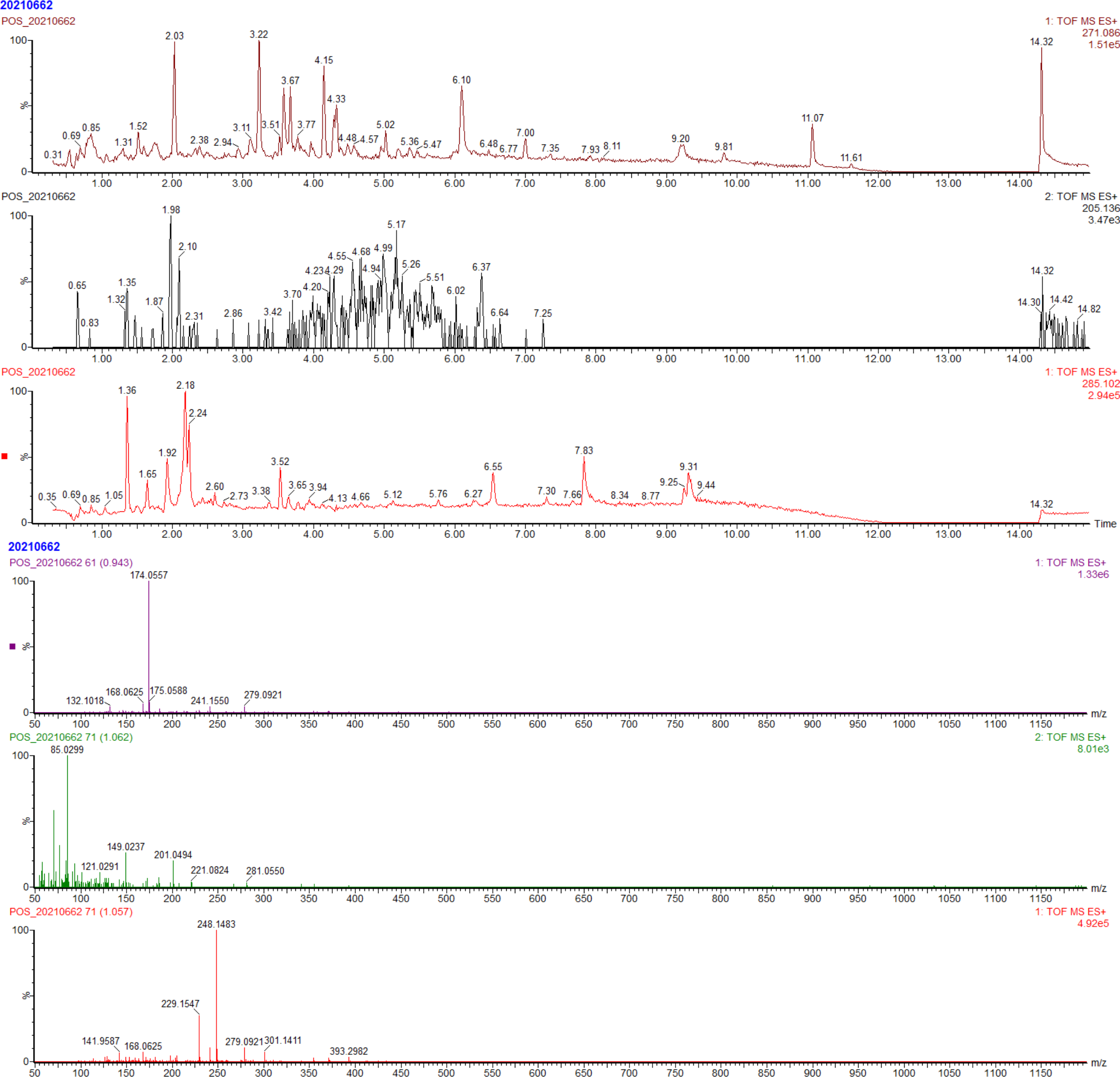

Supplement: Supplementary material 1 — HPLC-MS chromatograms [file imafungus-17-e167329-s001.zip › Supplementary Fig. S6 ZRL20210662.png]

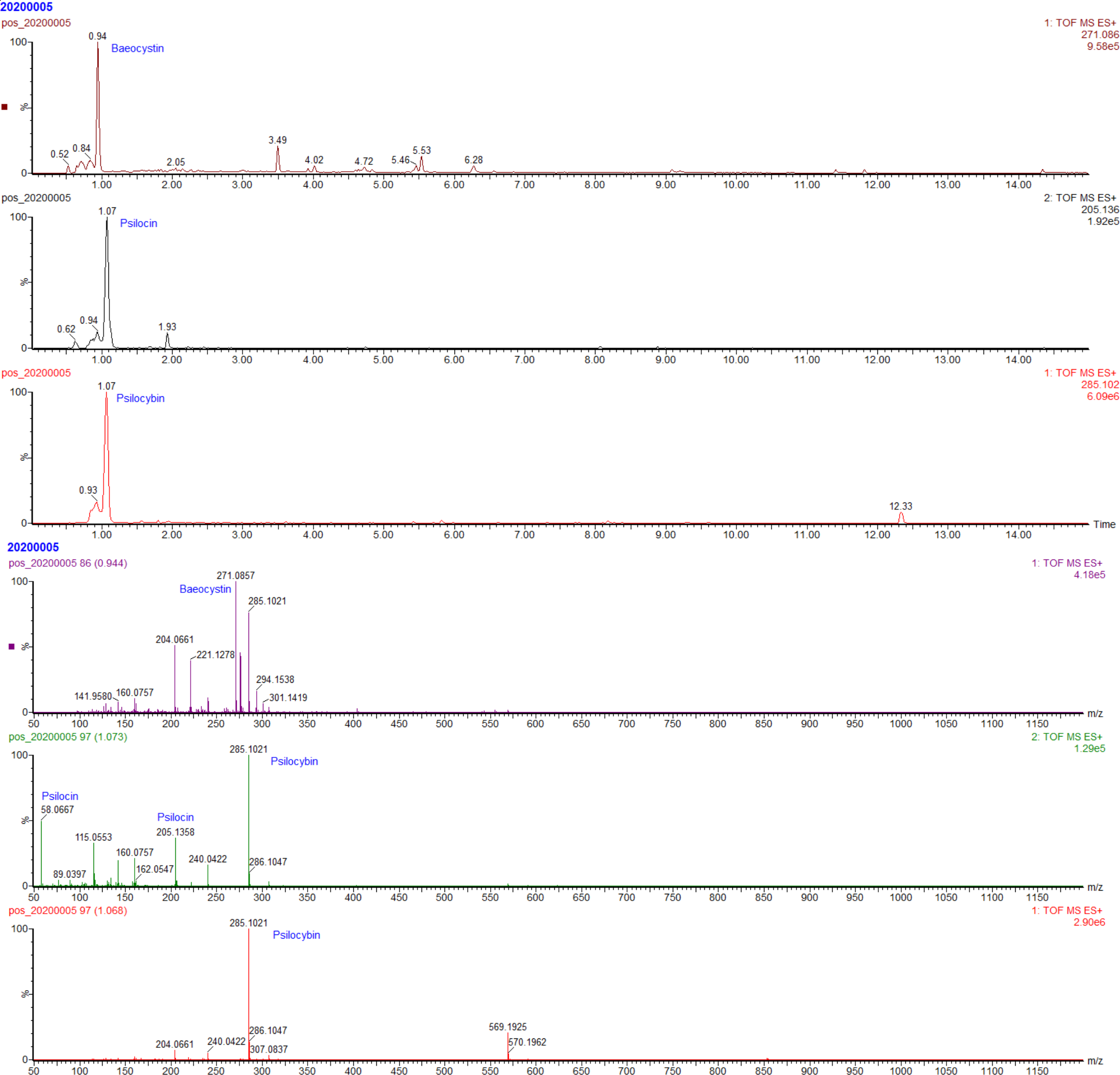

Supplement: Supplementary material 1 — HPLC-MS chromatograms [file imafungus-17-e167329-s001.zip › Supplementary Fig. S7 ZRL20200005.png]

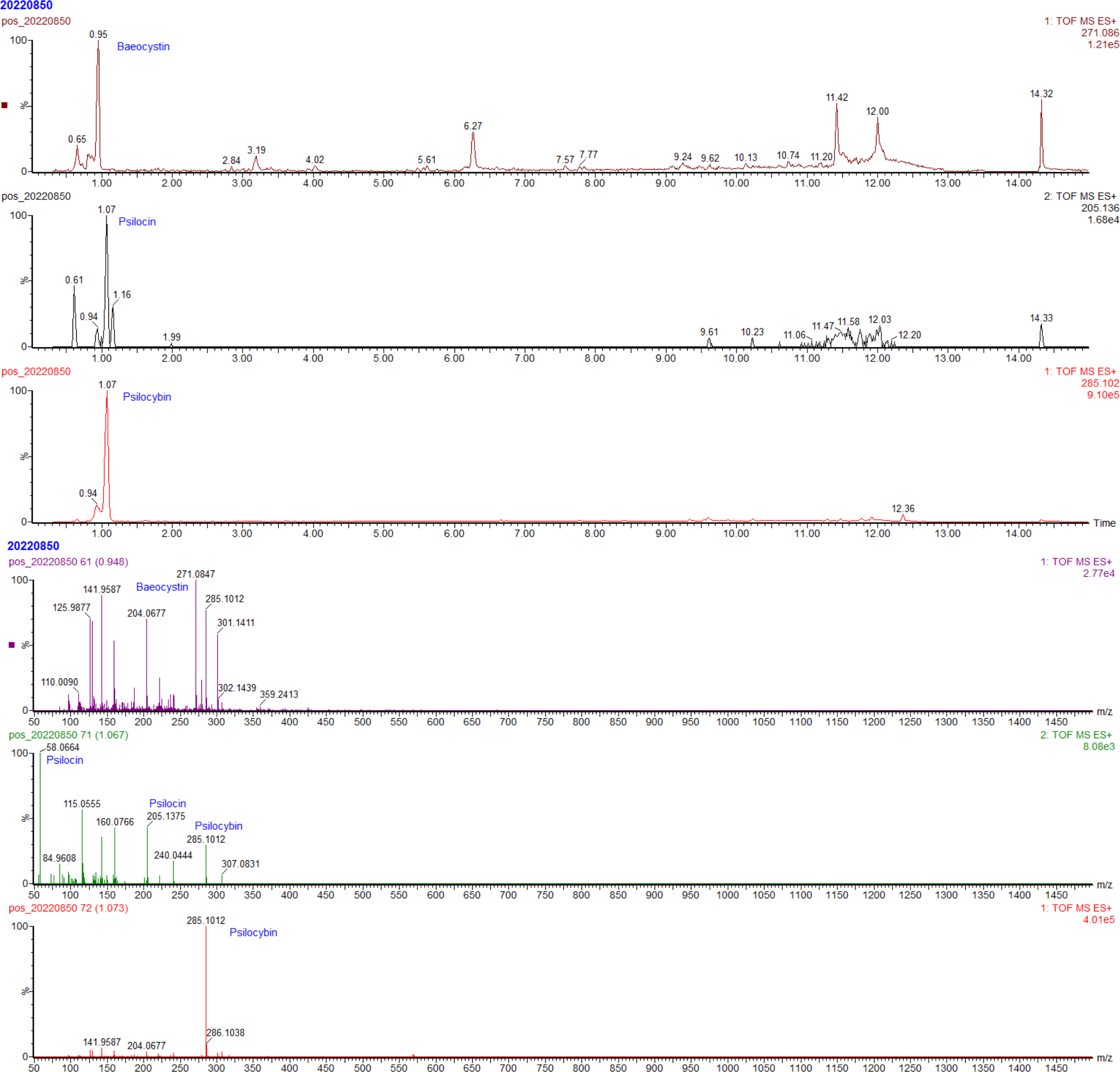

Supplement: Supplementary material 1 — HPLC-MS chromatograms [file imafungus-17-e167329-s001.zip › Supplementary Fig. S8 ZRL20220850.png]

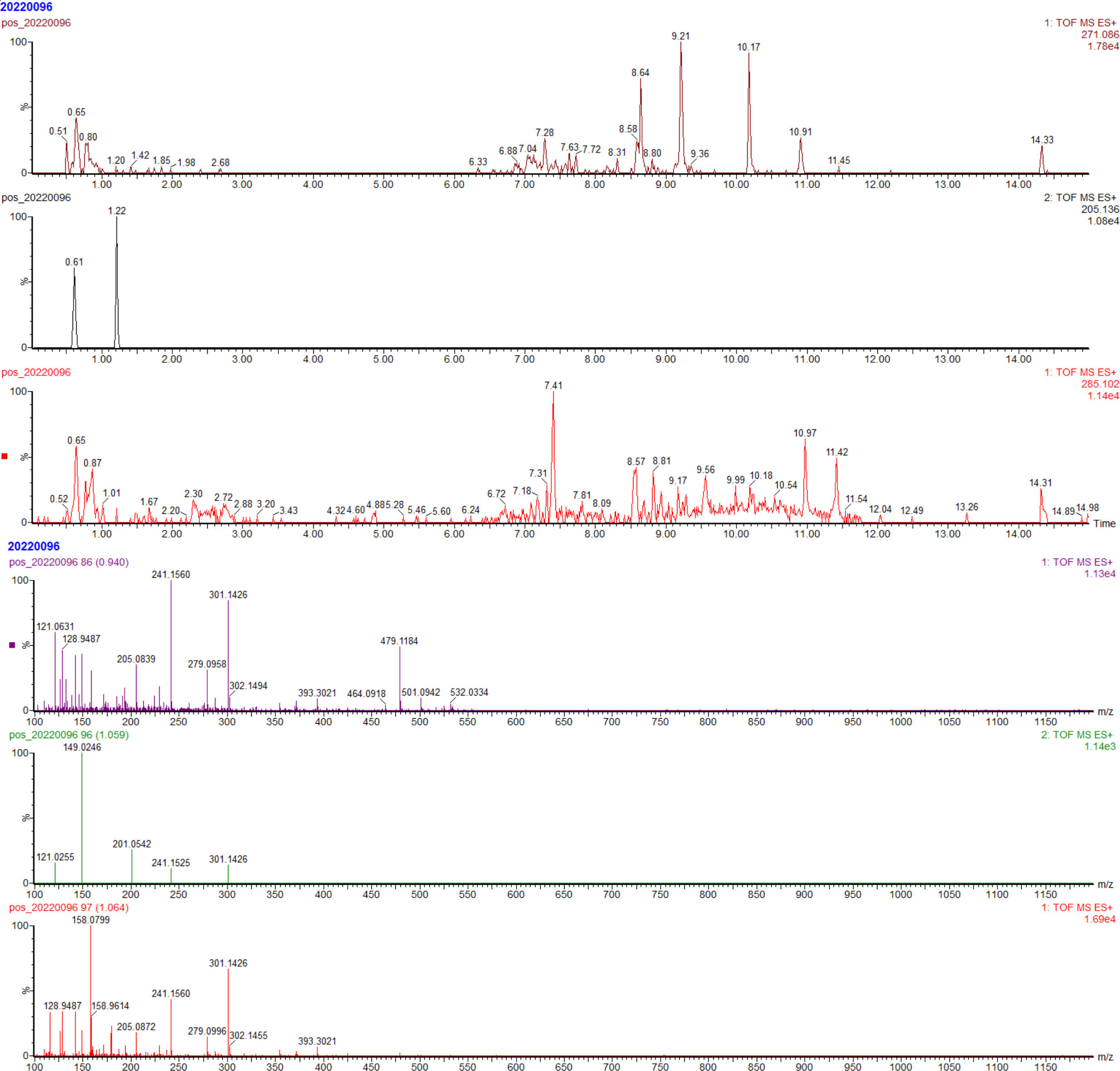

Supplement: Supplementary material 1 — HPLC-MS chromatograms [file imafungus-17-e167329-s001.zip › Supplementary Fig. S9 ZRL20220096.png]

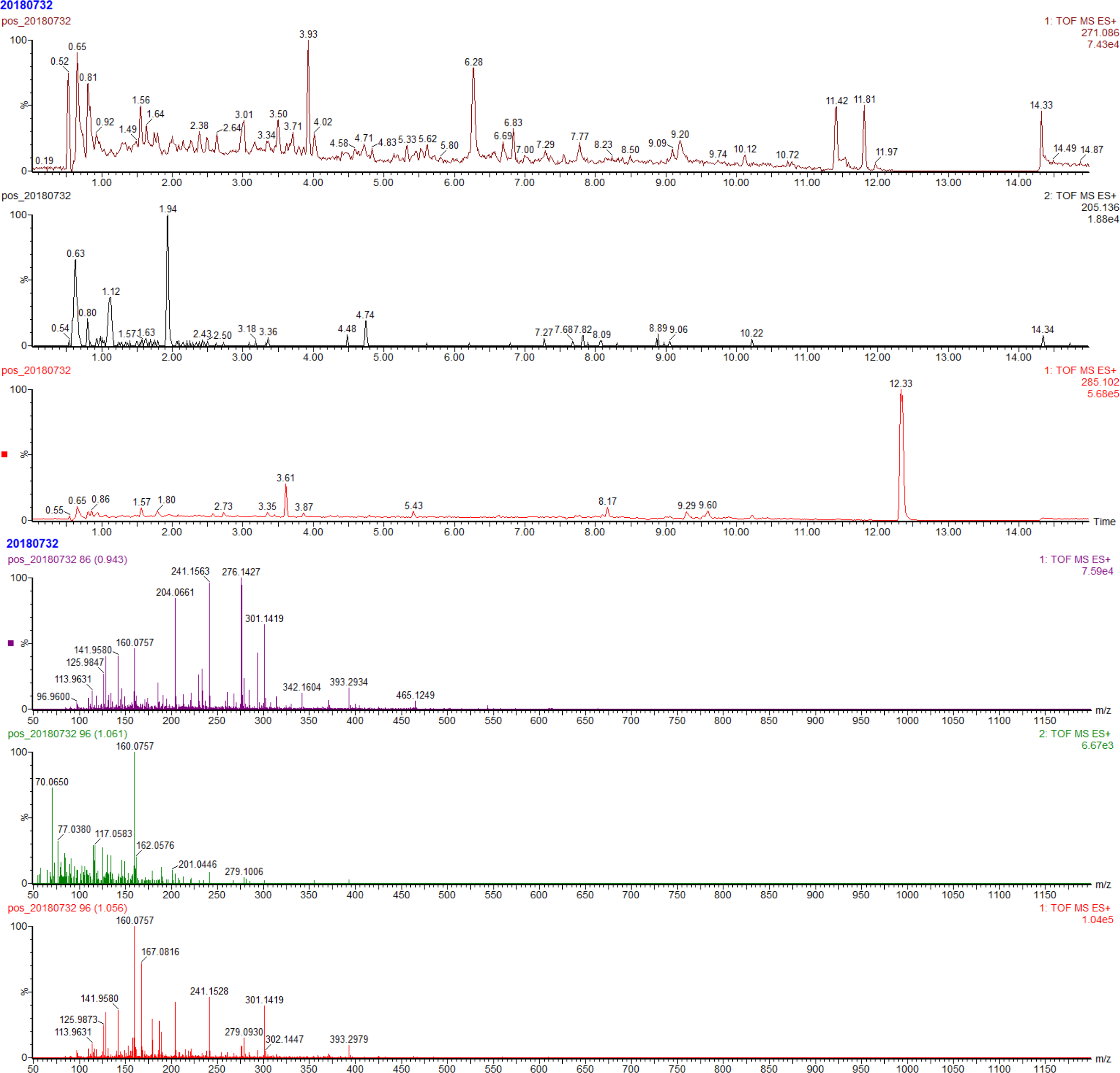

Supplement: Supplementary material 1 — HPLC-MS chromatograms [file imafungus-17-e167329-s001.zip › Supplementary Fig. S10 ZRL20180732.png]

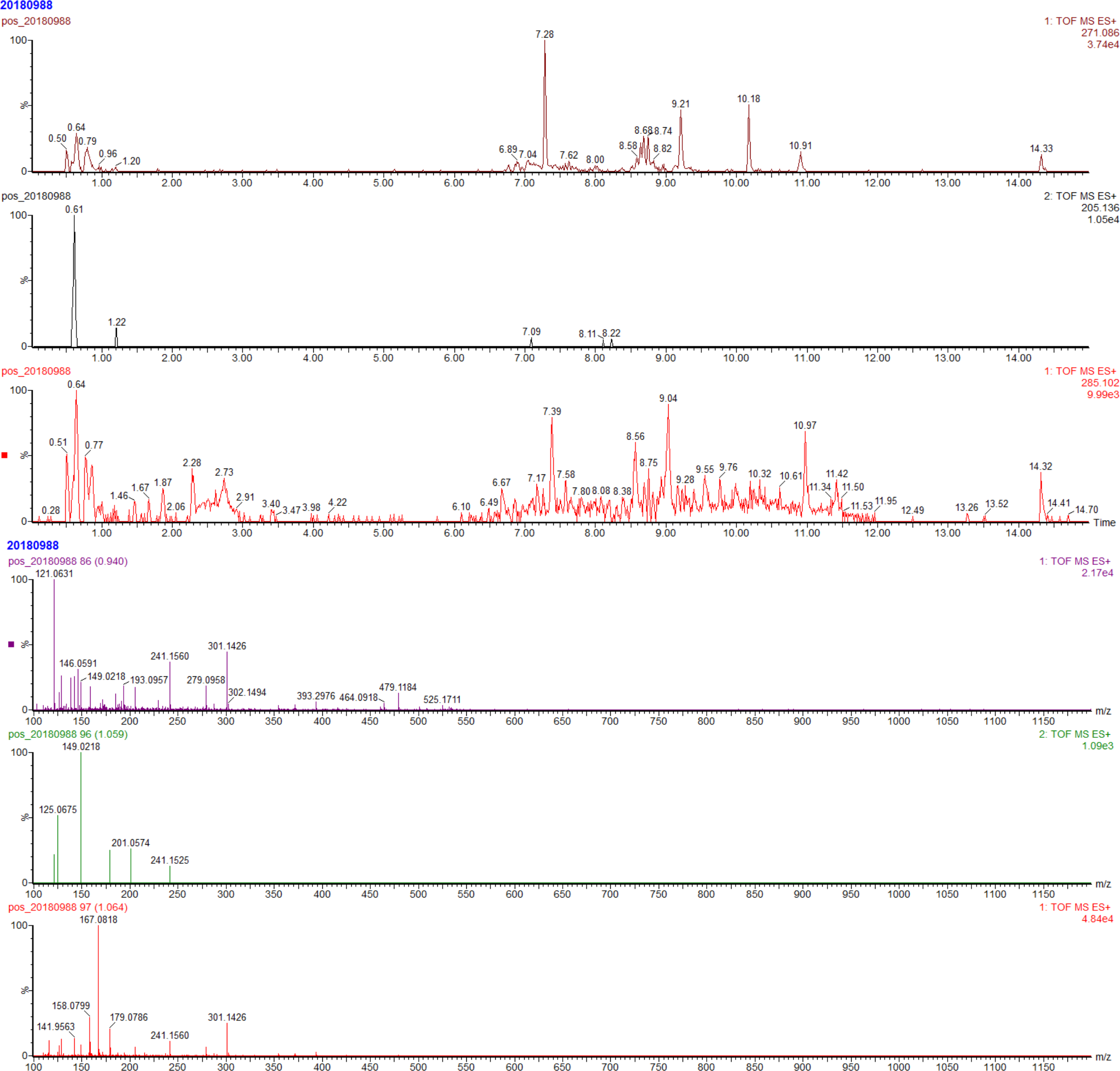

Supplement: Supplementary material 1 — HPLC-MS chromatograms [file imafungus-17-e167329-s001.zip › Supplementary Fig. S11 ZRL20180988.png]

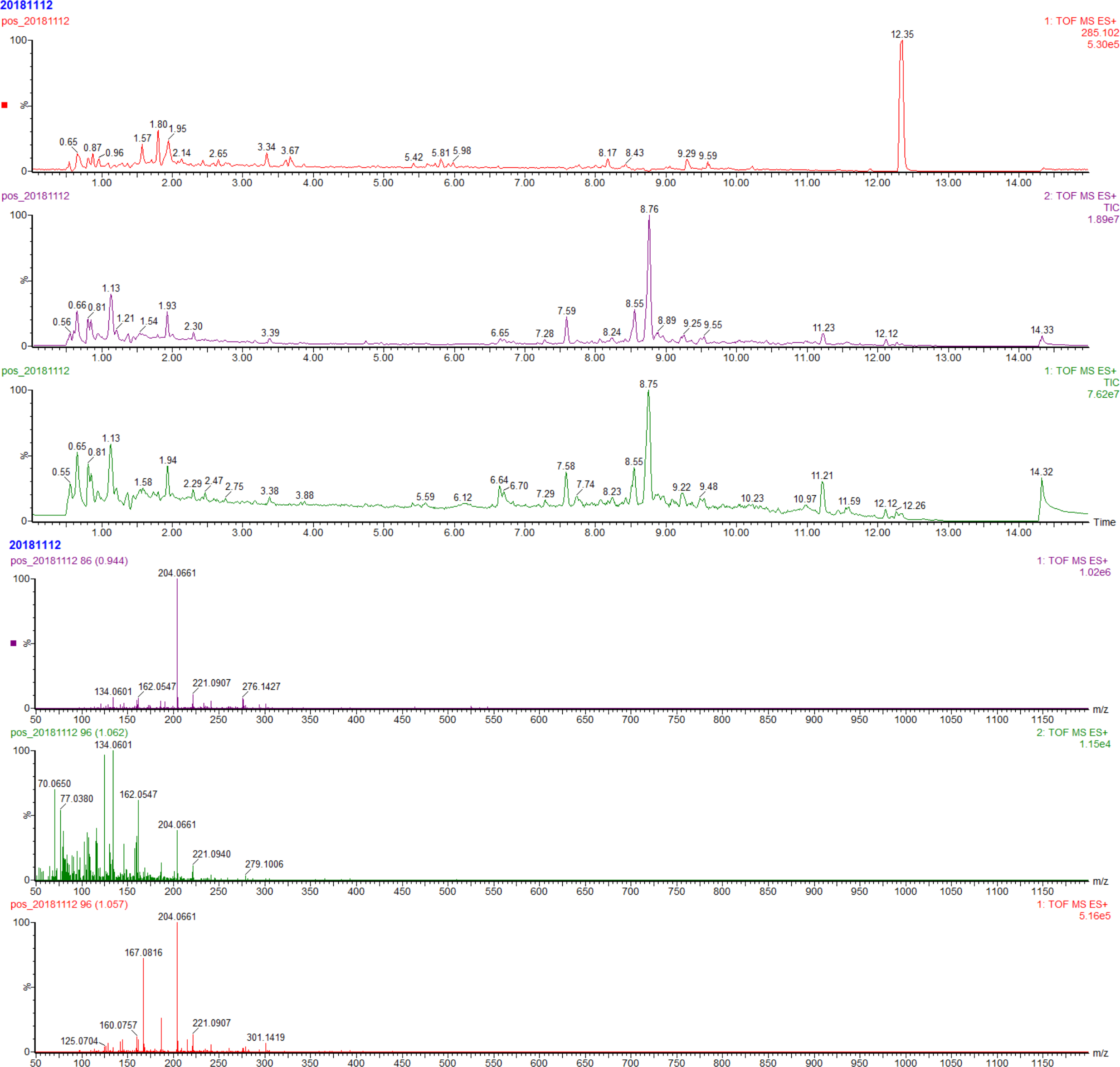

Supplement: Supplementary material 1 — HPLC-MS chromatograms [file imafungus-17-e167329-s001.zip › Supplementary Fig. S12 ZRL20181122.png]

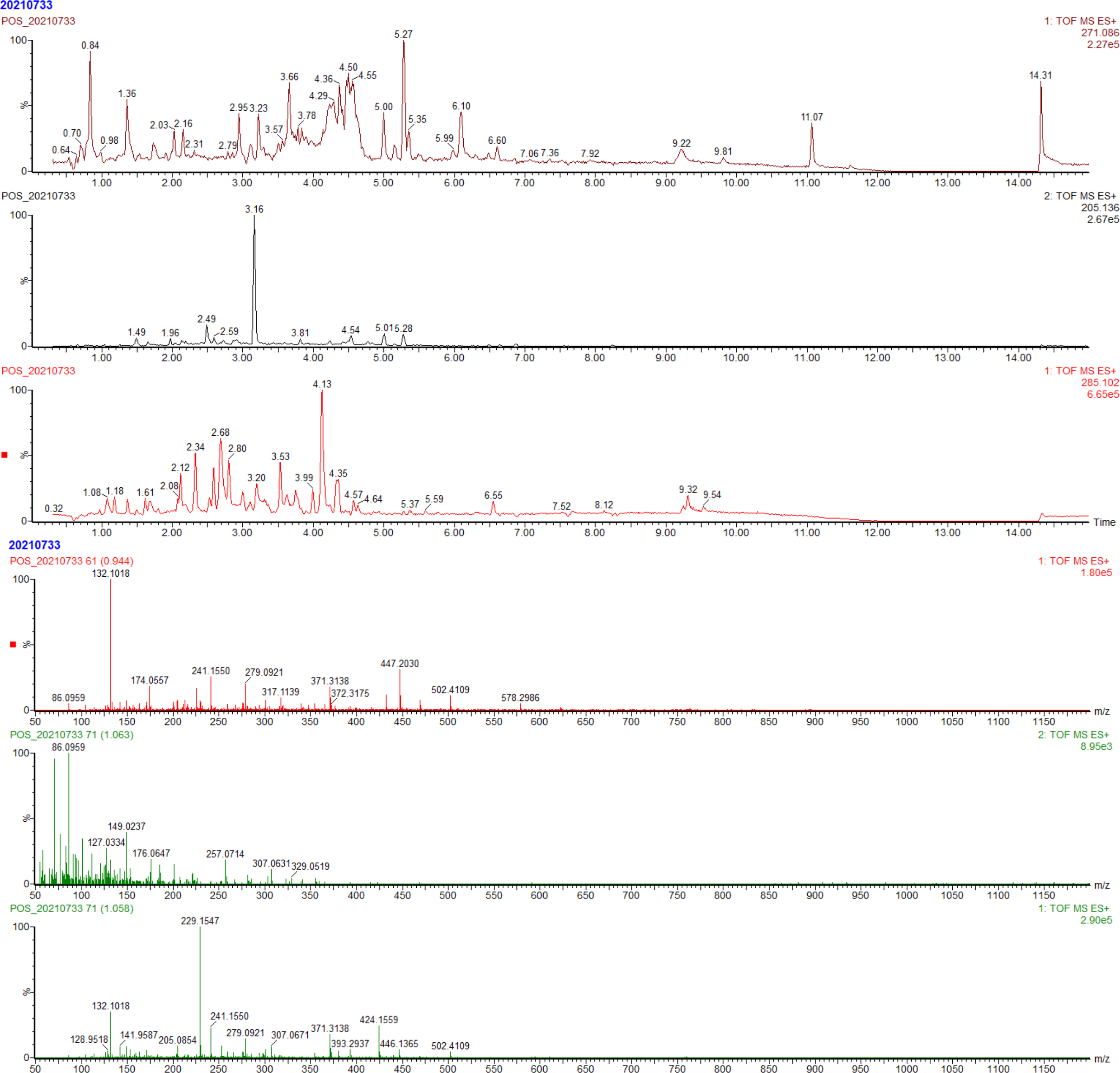

Supplement: Supplementary material 1 — HPLC-MS chromatograms [file imafungus-17-e167329-s001.zip › Supplementary Fig. S13 ZRL20210733.png]

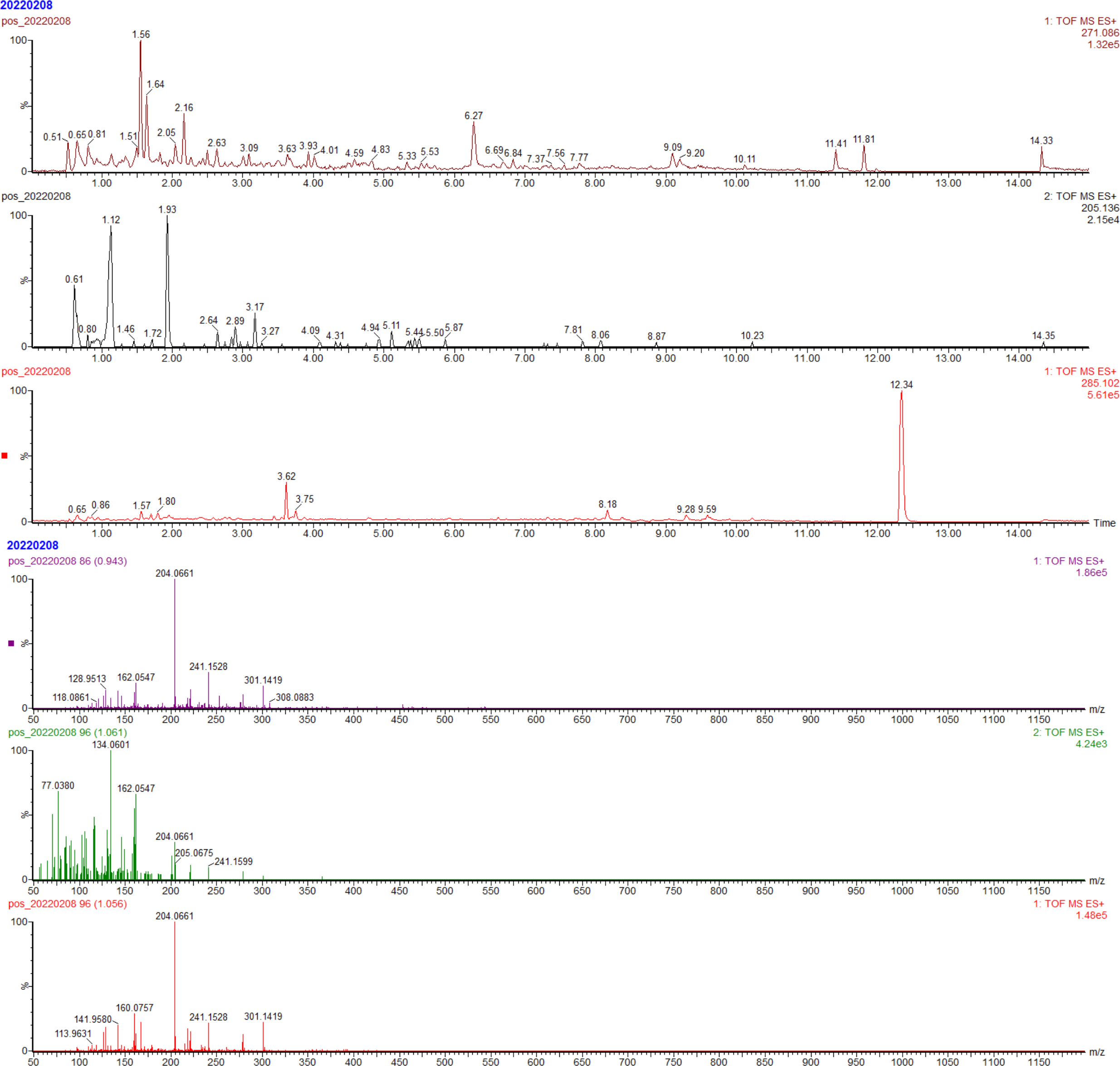

Supplement: Supplementary material 1 — HPLC-MS chromatograms [file imafungus-17-e167329-s001.zip › Supplementary Fig. S14 ZRL20220208.png]
